# Childhood-onset type 1 diabetes and subsequent adult psychiatric disorders: a nationwide cohort and genome-wide Mendelian randomization study

**DOI:** 10.1038/s44220-024-00280-8

**Published:** 2024-07-17

**Authors:** Tomáš Formánek, Danni Chen, Zdeněk Šumník, Karolína Mladá, James Hughes, Stephen Burgess, Nicholas J. Wareham, Graham K. Murray, Peter B. Jones, Benjamin I. Perry

**Affiliations:** 1 Department of Psychiatry, University of Cambridge, Cambridge, UK; 2 Department of Public Mental Health, National Institute of Mental Health, Klecany, Czechia; 3 Department of Clinical Epidemiology, Aarhus University, Aarhus, Denmark; 4 Department of Pediatrics, Motol University Hospital and 2nd Faculty of Medicine, Charles University, Prague, Czechia; 5 Department of Psychiatry, Faculty of Medicine in Pilsen, Charles University, Pilsen, Czechia; 6 Cambridgeshire and Peterborough NHS Foundation Trust, Cambridge, UK; 7 MRC Biostatistics Unit, University of Cambridge, Cambridge, UK; 8 MRC Epidemiology Unit, University of Cambridge, Cambridge, UK

**Keywords:** Type 1 diabetes, Psychiatric disorders, Epidemiology

## Abstract

Childhood-onset type 1 diabetes (T1D) is associated with substantial psychiatric morbidity in later life, but it remains unknown whether these associations are due to common underlying biological mechanisms or the impacts of living with the condition and its treatment. Here, using Czech national register data, we identified children with T1D aged ≤14 years between 1994 and 2007 and estimated the risk of psychiatric disorders up to 24 years later. We found that children diagnosed with T1D had an elevated risk of developing substance use, mood, anxiety and personality disorders, and behavioral syndromes. Conversely, we found that children with T1D had a lower risk of developing psychotic disorders. In Mendelian randomization analysis, we found an association with schizophrenia, which, however, did not persist following multiple testing adjustment. The combined observational and Mendelian randomization evidence suggests that T1D diagnosis in childhood predisposes to far-reaching, extensive psychiatric morbidity, which is unlikely to be explicable by common underlying biological mechanisms. The findings of this study highlight that monitoring and addressing the mental health needs of children with T1D is imperative, whereas glucose dysregulation and/or inflammation implicated in schizophrenia pathogenesis warrants future research.

## Abstract (web summary)

In this study, analyzing Czech national register-based data and using genome-wide Mendelian randomization, the authors report elevated risk for developing substance use, mood, anxiety and personality disorders in individuals with childhood-onset type 1 diabetes, and show that these associations are unlikely to be explicable by common underlying biological mechanisms.

## Main

Type 1 diabetes (T1D) is an autoimmune disease that leads to destruction of pancreatic β-cells and lifelong insulin deficiency^1^. It is thought to be multifactorial in origin, but its causes remain incompletely understood^1^. As T1D affects around 8.4 million individuals worldwide and its prevalence is expected to double in the next two decades^2^, it carries a considerable personal and societal burden, with adherence to a lifelong insulin requirement, a 10-year shortened life expectancy^3^ and a per-patient cumulative economic burden close to US$500,000 (ref. ^4^).

Evidence from large-scale population-based studies has consistently shown that substantial comorbidity exists between childhood-onset T1D and subsequent adult psychiatric disorders^5–7^. However, existing research has focused mostly on broadly defined psychiatric disorders and had only limited ambitions to ascertain whether the observed associations are best explained by the impacts of living with the condition and its treatment, or whether underlying common biological mechanisms may be implicated.

It is widely accepted that psychological and behavioral reactions may arise from the requirement for regular and frequent insulin injections, constant glucose monitoring, restrictions on diet and freedom of lifestyle, the prospect of short-term consequences of hypo- or hyperglycemia, and the long-term physical and potentially life-shortening complications of systemic diabetes manifestations. Children with T1D report higher levels of distress^8,9^, show more problem behaviors^10^ and report a lower quality of life than their peers^11^, and parents of children diagnosed with T1D report high levels of distress and family disruption^12^. Homo- or heterotypic continuity of childhood behavioral syndromes and the tracking of psychosocial putative causal factors into adult life might sufficiently explain the association between childhood T1D and adult psychiatric morbidity.

However, a biological link between childhood-onset T1D and psychiatric disorders is also possible. The brain undergoes dynamic changes during childhood and adolescence, requiring continuous glucose delivery for healthy development^13^. Childhood-onset T1D is longitudinally associated with persistent changes in global and regional brain volumes and cognition, partly explained by hypo- and hyperglycemic episodes^14^. Given that most psychiatric disorders peak in incidence by late adolescence^15^, unstable glycemic control during a critical neurodevelopmental period could predispose to wide-ranging consequences on psychiatric risk.

We aimed to address the limitations of existing research and take steps toward assessing potential causal pathways between childhood-onset T1D and psychiatric disorders using a two-step analytical approach designed to minimize bias and/or confounding. First, we used national register data from Czechia to investigate the association between T1D and subsequent psychiatric disorders. Then, we used independent bidirectional two-sample Mendelian randomization (MR) analyses of data from large-scale genome-wide association studies (GWAS) of European participants to further interrogate the associations.

## Results

### Observational analysis

Using data from the Czech nationwide register of all-cause hospitalizations, we identified 4,556 children with T1D aged ≤14 years between 1 January 1994 and 31 December 2007 (Supplementary Fig. 1). We subsequently matched each child with T1D with ten unique counterparts who had no T1D up to that point on sex, exact age, discharge year and discharge month (Supplementary Fig. 2). The mean age in both groups was 8.66 years (s.d. of 3.89 years), and the proportion of males was 52.81% (Table 1). Then, we used stratified Cox proportional hazards models to assess the risk of developing 6 psychiatric diagnostic groups and 21 specific or closely related psychiatric disorders during the follow-up period ranging from 10 to 24 years while also accounting for the competing risk of mortality (see also the proposed directed acyclic graph in Supplementary Fig. 3).

**Table 1:** Description of cohorts

	Matched counterparts	T1D cohort
Total, *n*	45,560	4,556
Male participants, *n* (%)	24,060 (52.81)	2,406 (52.81)
Age, mean (s.d.)	8.66 (3.89)	8.66 (3.89)
Discharge year on index hospitalization, median (IQR)	2000 (1996–2004)	2000 (1996–2004)
Discharge month on index hospitalization, median (IQR)	6 (3–10)	6 (3–10)

The results are presented as absolute numbers (*n*) with proportions (%), means with s.d. and medians with interquartile ranges (IQR). The distribution on sex, age, month and year at discharge is the same owing to exact matching on these characteristics.

### Risk of subsequent psychiatric disorders

Individuals with childhood-onset T1D were more likely to develop substance use disorders (hazard ratio (HR) = 1.39; 95% confidence interval (CI) = 1.23–1.58), mood disorders (HR = 2.32; 95% CI = 1.82–2.96), anxiety disorders (HR = 1.61; 95% CI = 1.40–1.85), behavioral syndromes associated with physiological disturbances and physical factors (HR = 4.18; 95% CI = 3.24–5.39), and personality and behavioral disorders (HR = 1.39; 95% CI = 1.09–1.77) during the follow-up period than their matched counterparts without T1D. Conversely, individuals with childhood-onset T1D were less likely to develop psychotic disorders (HR = 0.55; 95% CI = 0.33–0.91).

**Fig. 1:**
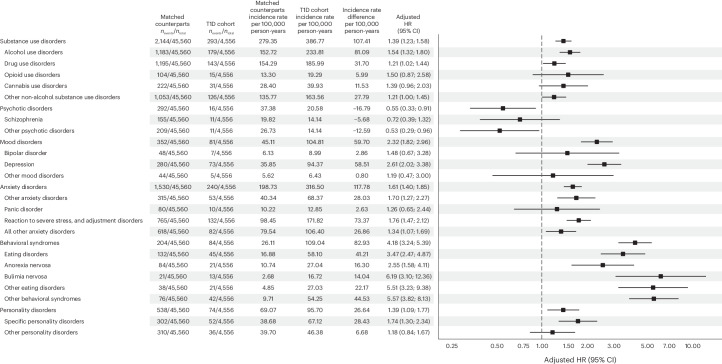
Risk of occurrence of psychiatric disorders in individuals with childhood-onset T1D. Incidence rates were calculated per 100,000 person-years. Incidence rate differences denote the difference between the incidence rate in individuals with T1D and their matched counterparts, and are expressed per 100,000 person-years. The associations between T1D and psychiatric disorders are expressed as adjusted HRs, accompanied by error bars expressed as 95% CIs.

Considering specific or closely related psychiatric disorders, individuals with childhood-onset T1D had an elevated risk of developing 12 out of the 21 of these during the follow-up period: alcohol use disorders (HR = 1.54; 95% CI = 1.32–1.80), drug use disorders (HR = 1.21; 95% CI = 1.02–1.44), depression (HR = 2.61; 95% CI = 2.02–3.38), other anxiety disorders (HR = 1.70; 95% CI = 1.27–2.27), reaction to severe stress, and adjustment disorders (HR = 1.76; 95% CI = 1.47–2.12), all other anxiety disorders (HR = 1.34; 95% CI = 1.07–1.69), eating disorders (HR = 3.47; 95% CI = 2.47–4.87), anorexia nervosa (HR = 2.55; 95% CI = 1.58–4.11), bulimia nervosa (HR = 6.19; 95% CI = 3.10–12.36), other eating disorders (HR = 5.51; 95% CI = 3.23–9.38), other behavioral syndromes (HR = 5.57; 95% CI = 3.82–8.13) and specific personality disorders (HR = 1.74; 95% CI = 1.30–2.34). Conversely, individuals with childhood-onset T1D were less likely to develop other psychotic disorders (HR = 0.53; 95% CI = 0.29–0.96). For the other outcomes, the 95% CIs were consistent with a null effect. See Fig. 1 for detailed results, Supplementary Figs. 4–30 for cumulative events plots and Supplementary Table 1 for the sex-stratified results.

### Sensitivity analyses of observational data

We investigated the robustness of our findings by exploring the potential impact of different sources of bias and/or residual confounding on our estimates. The results of analysis of (1) children diagnosed with T1D up to 9 years of age and (2) incident T1D cases, assessing the potential impact of reverse causality and selection bias, respectively, did not diverge from the results of the main analysis for any of the outcomes.

When considering the number of previous hospitalizations to explore potential informative presence bias^16^, our results differed from the main analysis for personality disorders, for which the 95% CI covered a range from a decreased to an increased risk (HR = 1.28; 95% CI = 0.97–1.69). Further exploring potential informative presence bias, the results of comparison with children with asthma diverged from the main analysis for substance use disorders and personality disorders, with both of them being consistent with a null effect (HR = 1.13; 95% CI = 0.99–1.30 and HR = 1.05; 95% CI = 0.80–1.37). Detailed results are provided in Table 2, Supplementary Tables 2–5 and Supplementary Figs. 31–34.

**Table 2:** Risk of occurrence of psychiatric disorders in individuals with childhood-onset T1D across sensitivity analyses

Outcome	Sensitivity analysis			
	T1D diagnosed up to 9 years of age	Incident cases of T1D	Adjustment for the number of previous hospitalizations	Comparison with individuals with asthma
Substance use disorders	1.38 (1.13; 1.68)	1.68 (1.32; 2.13)	1.32 (1.15; 1.52)	1.13 (0.99; 1.30)
Alcohol use disorders	1.57 (1.23; 1.99)	1.87 (1.40; 2.49)	1.70 (1.42; 2.04)	1.31 (1.10; 1.57)
Drug use disorders	1.08 (0.78; 1.48)	1.26 (0.84; 1.87)	1.01 (0.83; 1.23)	0.91 (0.75; 1.10)
Opioid use disorders	NA	NA	1.04 (0.56; 1.93)	1.56 (0.82; 2.97)
Cannabis use disorders	0.88 (0.45; 1.74)	0.96 (0.44; 2.08)	1.09 (0.72; 1.65)	1.09 (0.71; 1.65)
Other non-alcohol substance use disorders	1.08 (0.76; 1.53)	1.26 (0.81; 1.97)	0.98 (0.80; 1.22)	0.90 (0.73; 1.10)
Psychotic disorders	NA	NA	0.52 (0.30; 0.88)	0.47 (0.28; 0.80)
Schizophrenia	NA	NA	0.67 (0.34; 1.30)	0.50 (0.26; 0.98)
Other psychotic disorders	NA	NA	0.43 (0.23; 0.83)	0.48 (0.25; 0.91)
Mood disorders	2.63 (1.76; 3.94)	2.21 (1.28; 3.79)	2.10 (1.59; 2.77)	1.79 (1.35; 2.37)
Bipolar disorder	NA	NA	1.25 (0.55; 2.86)	0.82 (0.35; 1.89)
Depression	2.62 (1.67; 4.11)	2.67 (1.48; 4.81)	2.39 (1.77; 3.22)	2.12 (1.56; 2.88)
Other mood disorders	NA	NA	1.35 (0.49; 3.67)	0.80 (0.30; 2.16)
Anxiety disorders	1.65 (1.35; 2.02)	1.67 (1.33; 2.10)	1.50 (1.29; 1.75)	1.19 (1.03; 1.39)
Other anxiety disorders	1.77 (1.15; 2.74)	1.89 (1.20; 2.99)	1.39 (1.01; 1.91)	1.18 (0.86; 1.63)
Panic disorder	1.38 (0.54; 3.51)	NA	1.40 (0.67; 2.93)	1.08 (0.52; 2.24)
Reaction to severe stress, and adjustment disorders	1.84 (1.38; 2.47)	2.19 (1.59; 3.03)	1.97 (1.60; 2.43)	1.37 (1.11; 1.70)
All other anxiety disorders	1.50 (1.09; 2.05)	1.18 (0.81; 1.72)	1.05 (0.81; 1.36)	0.90 (0.70; 1.16)
Behavioral syndromes	2.87 (1.92; 4.28)	2.38 (1.44; 3.93)	3.20 (2.39; 4.27)	2.33 (1.73; 3.14)
Eating disorders	1.73 (0.93; 3.18)	2.17 (1.09; 4.30)	2.70 (1.83; 3.98)	2.07 (1.38; 3.10)
Anorexia nervosa	NA	NA	1.97 (1.14; 3.40)	1.66 (0.95; 2.89)
Bulimia nervosa	NA	8.25 (2.52; 27.05)	4.54 (2.00; 10.29)	2.37 (1.11; 5.06)
Other eating disorders	2.33 (1.02; 5.30)	4.64 (1.89; 11.39)	4.77 (2.56; 8.87)	2.68 (1.40; 5.11)
Other behavioral syndromes	5.20 (3.07; 8.82)	3.10 (1.57; 6.10)	3.99 (2.61; 6.09)	2.59 (1.69; 3.97)
Personality disorders	1.72 (1.20; 2.46)	2.07 (1.33; 3.21)	1.28 (0.97; 1.69)	1.05 (0.80; 1.37)
Specific personality disorders	2.07 (1.35; 3.19)	2.76 (1.61; 4.73)	1.44 (1.04; 1.99)	1.24 (0.89; 1.72)
Other personality disorders	1.45 (0.84; 2.49)	1.26 (0.60; 2.63)	1.29 (0.86; 1.92)	0.94 (0.64; 1.37)

NA denotes a situation when the number of outcomes in either the individuals with T1D or their matched counterparts was less than 5 (for details, see Supplementary Methods). The associations between T1D and psychiatric disorders are expressed as adjusted HRs, accompanied by 95% CIs.

*E* values, indicating the strength of association that a potential confounder would need to have with both the exposure and the outcome to explain away the associations detected in the main models^17^, ranged from 1.71 for other non-alcohol substance use disorders to 11.86 for bulimia nervosa, with a median of 2.97 (Supplementary Table 6). When considering the effect of unmeasured confounders^18^, the weighted average of percent bias was below 10% for each outcome (Supplementary Table 7), indicating that the potential inclusion of unmeasured confounders would change our results by less than 10%.

### MR analysis

To further explore the potential impact of residual confounding on associations that we identified in the observational analysis, we used univariable two-sample MR. MR is a genetic epidemiological method that uses summary data from GWAS to model genetic variants, fixed at conception, as instrumental variables to explore direction of association free of residual confounding. For T1D, we included several exposure instruments: a *trans* instrument featuring all genome-wide significant T1D variants and six *cis* instruments featuring single-nucleotide polymorphisms (SNPs) in gene regions associated with childhood-onset T1D^19^. Summary statistics for genetic variants were obtained from the most up-to-date and largest publicly available GWAS of European participants (Supplementary Table 8).

For schizophrenia, using our primary analysis method of inverse variance weighted (IVW) regression, we demonstrated that per one-unit increase in genetically predicted log odds of T1D as measured by the *trans* instrument, there was a 4% decrease in odds (odds ratio (OR) = 0.96; 95% CI = 0.93–0.99; *P* = 0.019). These results were consistent across secondary MR analysis methods, which can be more robust to potential bias-inducing pleiotropic effects of the genetic variants under different assumptions (weighted median OR = 0.96; 95% CI = 0.92–0.99; *P* = 0.032 and MR-Egger OR = 0.94; 95% CI = 0.89–0.99; *P* = 0.018), but none of these associations persisted following adjustment for multiple testing (adjusted *P* = 0.133, 0.096 and 0.072, respectively). We detected a similar inverse association when using the *CTSH cis* instrument and our primary IVW method (OR = 0.82; 95% CI = 0.68–0.99; *P* = 0.042), but it did not persist following adjustment for multiple testing (adjusted *P* = 0.252). In addition, we detected an opposing effect direction for the *GLIS3 cis* instrument using the secondary weighted median method (OR = 1.10; 95% CI = 1.03–1.19; *P* = 0.007; adjusted *P* = 0.028).

For anxiety disorders, per one-unit increase in genetically predicted log odds of T1D as measured by the *GLIS3 cis* instrument, there was a 46% increase in odds using our primary IVW method (OR = 1.46; 95% CI = 1.22–1.75; *P* < 0.001; adjusted *P* < 0.001). The results were consistent when using the secondary weighted median method (OR = 1.39; 95% CI = 1.11–1.74; *P* = 0.004; adjusted *P* = 0.016).

For anorexia nervosa, per one-unit increase in genetically predicted log odds of T1D as measured by the *IL2RA cis* instrument, there was a 12% decrease in odds using the secondary weighted median method only (OR = 0.88; 95% CI = 0.81–0.96; *P* = 0.003; adjusted *P* = 0.012).

For major depressive disorders, per one-unit increase in genetically predicted log odds of T1D as measured by the *GLIS3 cis* instrument was associated with a 7% increase in odds using the secondary weighted median method only (OR = 1.07; 95% CI = 1.00–1.15; *P* = 0.032), but this association did not persist following adjustment for multiple testing (adjusted *P* = 0.128).

For alcohol dependence, per one-unit increase in genetically predicted log odds of T1D as measured by the *GLIS3 cis* instrument, there was a 22% decrease in odds using the secondary weighted median method only (OR = 0.78; 95% CI = 0.65–0.78; *P* = 0.017); this association did not persist following adjustment for multiple testing (adjusted *P* = 0.068).

For all other analyses, we did not find any evidence for associations of genetically predicted T1D with psychiatric outcomes (Table 3, Supplementary Table 9 and Supplementary Figs. 35–46). In bidirectional analysis, we did not find evidence for associations of genetically predicted psychiatric disorders with T1D, suggesting no evidence for reverse causality (Supplementary Table 10).

**Table 3:** Main MR results

Exposure	Outcome	OR (95% CI)	*P* value	Adjusted *P* value
T1D *trans* instrument	Alcohol dependence	1.01 (0.96; 1.09)	0.600	1.000
	Schizophrenia	0.96 (0.93; 0.99)	0.019	0.133
	Bipolar disorder	0.98 (0.94; 1.02)	0.374	1.000
	Major depressive disorder	0.99 (0.97; 1.02)	0.523	1.000
	Anxiety disorders	0.94 (0.89; 1.00)	0.064	0.384
	Anorexia nervosa	0.97 (0.94; 1.01)	0.135	0.708
T1D *GLIS3 cis* instrument	Alcohol dependence	0.91 (0.76; 1.34)	0.230	1.000
	Schizophrenia	1.00 (0.98; 1.02)	0.991	1.000
	Bipolar disorder	1.05 (0.96; 1.18)	0.317	1.000
	Major depressive disorder	0.99 (0.98; 1.01)	0.146	1.000
	Anxiety disorders	1.46 (1.22; 1.75)	<0.001	<0.001
	Anorexia nervosa	1.00 (0.98; 1.03)	0.999	1.000
T1D *CTSH cis* instrument	Alcohol dependence	0.74 (0.41; 1.36)	0.337	1.000
	Schizophrenia	0.82 (0.68; 0.99)	0.042	0.252
	Bipolar disorder	0.93 (0.67; 1.28)	0.660	1.000
	Major depressive disorder	1.02 (0.85; 1.23)	0.811	1.000
	Anxiety disorders	0.52 (0.23; 1.20)	0.124	0.620
	Anorexia nervosa	0.97 (0.68; 1.40)	0.890	1.000
T1D *IKZF3 cis* instrument	Alcohol dependence	0.75 (0.36; 1.56)	0.448	1.000
	Schizophrenia	0.83 (0.63; 1.10)	0.205	1.000
	Bipolar disorder	0.68 (0.43; 1.05)	0.084	0.504
	Major depressive disorder	0.91 (0.70; 1.18)	0.482	1.000
	Anxiety disorders	0.69 (0.29; 1.65)	0.405	1.000
	Anorexia nervosa	1.53 (0.98; 2.38)	0.059	1.000
T1D *IL2RA cis* instrument	Alcohol dependence	0.80 (0.55; 1.17)	0.257	1.000
	Schizophrenia	0.93 (0.80; 1.07)	0.292	1.000
	Bipolar disorder	0.95 (0.77; 1.17)	0.620	1.000
	Major depressive disorder	0.95 (0.84; 1.08)	0.436	1.000
	Anxiety disorders	0.83 (0.54; 1.27)	0.394	1.000
	Anorexia nervosa	0.89 (0.72; 1.11)	0.319	1.000
T1D *IL10 cis* instrument	Alcohol dependence	1.18 (0.69; 2.01)	0.541	1.000
	Schizophrenia	1.01 (0.82; 1.25)	0.913	1.000
	Bipolar disorder	1.13 (0.81; 1.57)	0.469	1.000
	Major depressive disorder	0.98 (0.91; 1.18)	0.828	1.000
	Anxiety disorders	0.85 (0.47; 1.64)	0.679	1.000
	Anorexia nervosa	1.10 (0.80; 1.51)	0.554	1.000
T1D *THEMIS cis* instrument	Alcohol dependence	2.29 (0.61; 8.65)	0.218	1.000
	Schizophrenia	0.88 (0.55; 1.40)	0.586	1.000
	Bipolar disorder	0.98 (0.48; 2.01)	0.959	1.000
	Major depressive disorder	0.97 (0.64; 1.49)	0.902	1.000
	Anxiety disorders	1.11 (0.27; 4.59)	0.887	1.000
	Anorexia nervosa	1.79 (0.86; 3.71)	0.118	0.708

We used IVW OR for *trans* instrument, correlation-adjusted IVW OR for *cis* instruments with ≥2 SNPs or Wald ratio for *cis* instruments with <2 SNPs. The estimated effects represent the change in odds of outcome per s.d. increase in genetically predicted T1D risk and are accompanied by 95% CIs. Adjusted *P* values were computed using the Holm–Bonferroni method per T1D instrument.

### MR sensitivity analyses

We conducted a range of sensitivity analyses to explore the robustness of our MR results. Tests of instrument strength indicated no strong evidence for weak instrument bias (Supplementary Tables 11 and 12). Cochran’s *Q* and MR-Egger intercept tests indicated no evidence for horizontal pleiotropy but some evidence of heterogeneity specifically for some analyses of schizophrenia, anxiety disorders and anorexia nervosa (Supplementary Table 13). MR-PRESSO indicated the presence of influential outliers for analyses of schizophrenia and anorexia nervosa for the *trans* instrument only. For schizophrenia, the results of outlier-corrected IVW did not materially change; however, evidence for anorexia nervosa strengthened (Supplementary Table 14). *I*^2^_GX_ statistics (representing an adaptation of the *I*^2^ heterogeneity statistic from meta-analysis, related to the degree of dilution of the causal effect estimate) were all above 0.597 (Supplementary Table 15). Post hoc power analyses (Supplementary Table 16) suggested likely limitations in being able to detect particularly subtle potential causal effects for all psychiatric outcomes.

## Discussion

We used independent observational and genetic epidemiological analyses to investigate the potential causal pathways underlying associations of childhood-onset T1D and subsequent psychiatric disorders. On the basis of Czech national register data, we found that individuals diagnosed with T1D in childhood have a lower risk of developing psychotic disorders but a higher risk of subsequently developing the majority of other studied psychiatric disorders compared with children without T1D. Results for mood and anxiety disorders and behavioral syndromes were robust to reverse causality, selection and informative presence bias, and quantitative bias analyses implied that the results were unlikely to be fully explained by confounding. The results of MR analysis were largely consistent in their support of a potential inverse causal relationship of T1D and psychotic disorders or schizophrenia, although evidence weakened following adjustment for multiple testing. There was at best only a limited consistency in MR evidence for all other studied psychiatric disorders.

Our observational findings are consistent with the results from two other national register studies. First, a Swedish study matched individuals diagnosed with T1D before 18 years of age on sex, year and country of birth with individuals with no recorded history of T1D, and found an increased risk for mood, anxiety, eating and substance use disorders after adjusting for a range of potential confounders^5^. Second, a Danish study matched children diagnosed with T1D before 18 years of age with counterparts without history of T1D on sex and date of birth, and found an elevated risk for mood, anxiety and eating disorders in both boys and girls^6^.

We did not find consistent MR evidence in support of a potential causal relationship between T1D and most of the included psychiatric disorders. It is recognized that rather than being a binary disorder, T1D most likely lies on a continuum, starting with the presence of islet antibodies, progressing to glucose intolerance or dysglycemia before the emergence of clinical symptoms^20^. The progression to symptomatic disease—one that would be captured in our observational analysis—shows substantial variability, ranging from months to decades^20–22^. Because of the relative rarity of T1D, our MR analysis based on data from psychiatric outcome GWAS contained most likely few individuals with symptomatic and therefore diagnosed T1D. Consequently, it is plausible that we captured the contributing polygenic biological mechanisms of T1D in individuals who do not have a symptomatic and therefore diagnosed T1D. Individuals who have genetic propensity for T1D but who do not progress to symptomatic disease are unlikely to face the psychosocial burden associated with being diagnosed and thus treated. If our interpretation is correct, then the discrepant findings between observational and genetic epidemiological analyses in this study may be explained by an indirect pathway between T1D and psychiatric morbidity, through living with the condition and its treatment, that we captured in the observational, but not in MR, analysis.

Children with T1D are indeed forced to make wholesale life adjustments with stringent, unrelenting focus on dietary monitoring and a high burden of daily management tasks^23^. Consequently, children with T1D may feel excluded from social events and singled out by peers, teachers and even family members^24^. ‘Diabetes distress’, including extreme frustration with blood sugars and feelings of isolation, can lead to burnout, hopelessness and a shift toward an external locus of control^25^. Furthermore, the implications of diabetes distress span into adolescence, since childhood-onset T1D may preclude achievement of developmental tasks pertinent to emerging adulthood^26^, leading to uncertainty surrounding identity and an increased risk of mental illness in adult life^27^. This underscores the critical importance of initiatives to proactively screen and monitor children diagnosed with T1D for emergent psychiatric disorders^28^.

Separately, we found more consistent evidence between observational and MR analyses for an inverse association between T1D and psychotic disorders or schizophrenia, although it was weakened following adjustment for multiple testing. This evidence, also consistent with results from a register-based study from Finland^29^, may herald some insights into schizophrenia pathogenesis and warrant specific investigation in future research. T1D is likely to be associated with instabilities in levels of both circulating insulin and glucose levels, particularly in the pre-diagnostic phases, where it is also associated with inflammation. Insulin receptors are widely expressed in the brain, with notable concentrations in regions of the brain known to be associated with schizophrenia^30,31^. Therefore, abnormal glucose–insulin signaling during a critical period of neurodevelopment, for example, in childhood and adolescence, may disrupt biological mechanisms that alter schizophrenia risk, in either a risk-decreasing or -increasing manner. For example, the direction of effect for the *GLIS3 cis* instrument switched, implying a positive association with schizophrenia. Previous work has shown that loci within the *GLIS3* gene region have a shared effect on both T1D and type 2 diabetes risks via effects on pancreatic β-cell function, insulin sensitivity and inflammation^32^. Childhood fasting insulin levels have recently been shown to be longitudinally associated with psychosis risk in adulthood^33^, and previous MR evidence has shown the importance of inflammation as a potential common cause for schizophrenia and insulin resistance^34^.

This study has some limitations. First, our observational outcomes were measured using data from inpatient services; however, a large proportion of these will be diagnosed and managed in community settings. This would suggest that T1D may accentuate the severity of emergent psychiatric disorders, rather than cause them per se, and potentially contribute to limited generalizability of our findings to all healthcare settings. Second, the approaches to address potential sources of bias and/or confounding had their own limits. In particular, external adjustment for unmeasured confounders relied on information in existing literature; however, for multiple exposure–outcome pairs, information on only a limited number of confounders was available, contributing to differing levels of confidence that the detected associations are not due to unmeasured confounders. Third, the number of events for certain outcomes was very small, leading to considerable uncertainty in the estimates. Fourth, individuals with T1D were allowed to be used as matched counterparts of other people with T1D before they developed T1D. While such cases were exceedingly rare, this led to a partial overlap between the groups and potentially contributed to a marginal underestimation of true effects. Fifth, we had no information on emigration status, and we cannot rule out that a proportion of individuals was lost to follow-up. Sixth, we restricted our analyses to psychiatric conditions that occur, on average, later in life^15^ to increase the confidence in the temporal order of conditions; however, investigating the associations between T1D and earlier-onset neurodevelopmental disorders such as autism spectrum disorder and attention deficit hyperactivity disorder in future studies is warranted. Seventh, MR evidence weakened following adjustment for multiple testing; thus, these results should be considered as suggestive and accepted with some caution. Eighth, it is possible that our MR results may be imprecise owing to statistical power. Despite using the largest GWAS available for exposures and outcomes, our post hoc MR power calculations showed a possible limitation in being able to detect particularly subtle causal effects. From this interpretation, it would follow that childhood-onset T1D may not be a sufficient solitary cause for psychiatric disorders but may interact with other known risk factors to subtly alter psychiatric risk. In future, replication of our work when larger GWAS for T1D, in particular childhood-onset, and psychiatric outcomes are available will be required. Ninth, we were unable to interrogate some observational associations with MR owing to the unavailability of GWAS data for some psychiatric outcomes. Lastly, all GWAS included in MR analyses were based on European samples, which reduced the risk of population stratification bias, but may limit the generalizability of findings to other populations.

## Conclusion

Through the combination of detailed observational and genetic epidemiological analyses, we provide insights into the wide-ranging and far-reaching psychiatric consequences of a T1D diagnosis in childhood. Our observational findings indicate higher risks of developing the majority of psychiatric disorders except psychoses in the proceeding decades after a childhood T1D diagnosis. However, we found a lack of consistent support for most studied psychiatric disorders in MR analysis, suggesting that these outcomes may be better explained by the psychological response to living with T1D and its treatment rather than by shared biological mechanisms. Separately, the inverse association between T1D and psychotic disorders or schizophrenia showed more consistency between observational and MR analyses, although MR evidence weakened following adjustment for multiple testing. Disentangling the potential mechanisms between T1D and schizophrenia warrants additional basic and experimental research efforts, but the results of this study clearly show that monitoring and addressing the mental health needs of children with T1D is imperative.

## Methods

### Observational analysis

#### Data

We used individual-level, de-identified data from Czech nationwide registers of (1) all-cause hospitalizations and (2) all-cause deaths, covering virtually the entire Czech population (approximately 10.7 million inhabitants). The Czech healthcare system is based on the compulsory insurance model, with the population coverage being virtually universal^35^. Beyond monitoring of public health, information from the registers is used to reimburse the service providers by insurance companies, thus increasing the confidence in their validity. Complete data between 1 January 1994 (the earliest available) and 31 December 2017 were used. This study was approved by the Ethics Committee of the National Institute of Mental Health (approval number 182/22). Owing to the legal mandate of the data, the analyses did not require informed consents from participants. See Supplementary Methods for further details.

#### Exposure

We included all hospitalizations between 1 January 1994 and 31 December 2007. We restricted the main analysis to individuals aged ≤14 years to limit the risk of reverse causality, because the incidence of examined psychiatric disorders before 14 years is thought to be modest^36^. The exposed cohort consisted of individuals with a recorded T1D diagnosis (International Classification of Diseases 10th Revision (ICD-10) code E10). We expected near-total detection of incident symptomatic T1D cases during the exposure window, since nearly all children with symptomatic T1D are initially hospitalized for treatment in Czechia^37^. The unexposed cohort consisted of (1) hospitalized individuals with no history of T1D during the examined time period and (2) the pre-T1D hospital records of individuals with T1D. Thus, an individual was allowed to be present both as an exposed individual and as an unexposed counterpart for another exposed individual. Exposed and unexposed individuals were included if they had no presence of a psychiatric disorder (F1–F6) listed at the index hospitalization. See Supplementary Methods for further details.

#### Outcome

Psychiatric disorders were assessed between the index hospitalization and 31 December 2017. Each individual was followed up between 10 and 24 years. We examined six ICD-10 psychiatric diagnostic groups (F1, F2, F3, F4, F5 and F6). We also examined 21 specific or closely related psychiatric disorders (F10, F11–F19, F11, F12, F13–F19, F20, F21–F29, F30–F31, F32–F33, F34–F39, F41, F410, F43, F40 or F42 or F44–F48, F50, F500–F501, F502–F503, F504–F509, F51–F59, F60 and F61–F69). See Supplementary Methods for further details.

#### Matching

We exact-matched each exposed individual with unexposed counterparts on age, sex, year and month at discharge from the exposed individuals’ index hospitalization. We randomly selected ten unique counterparts for each exposed individual. Sex and age at discharge from index hospitalization were considered important potential confounders; year and month at discharge ensured that the matched individuals would have a similar length of follow-up period and to control for possible cohort and calendar effects, respectively.

#### Statistical analysis

We provide baseline descriptive characteristics. We used stratified Cox proportional hazards models, with each stratum consisting of 11 individuals: 1 exposed and 10 matched unexposed counterparts. We considered the ‘event’ as the first recorded occurrence of a psychiatric disorder on a hospital record, examined separately for each included psychiatric disorder. We censored individuals who died or who did not experience the outcome during the follow-up period. Our observational analyses aimed to estimate the total potential causal effect of T1D diagnosis in childhood on the risk of subsequent psychiatric disorders. Results are expressed as HR with 95% CI, indicating the relative risk of developing psychiatric disorders in children with T1D compared with matched counterparts. Separate cumulative events plots were generated for each exposure–outcome pair. We used the R (4.0.3)^38^ libraries survival (3.2-7) and EValue (4.1.3)^39^ and avoided null-hypothesis significance tests^40^.

#### Sensitivity analyses of observational data

We performed several sets of sensitivity analyses to interrogate the robustness of our results under different scenarios, described in detail in Supplementary Methods. In brief, first, to further reduce the risk of including individuals who could have a psychiatric disorder before the index hospitalization (that is, reverse causality), we created cohorts with age at recorded T1D diagnosis restricted to 9 years or less.

Second, to account for the possibility that our main analysis contained prevalent cases that may have a higher disease severity, which, in turn, may increase the risk of experiencing the outcome, we restricted analysis to incident cases only. We did this by including only individuals for whom our data cover their entire lifespan.

Third, to account for the possibility that individuals with T1D might have more frequent healthcare interactions than their unexposed counterparts, which could lead to an increased chance of receiving a diagnosis of psychiatric disorder (informative presence bias^16^), we performed analysis after additionally matching children with T1D with up to 5 counterparts on the number of hospitalizations 3 years before the index hospitalization; we were not able to match 2 (0.05%) individuals. We also performed analysis comparing outcomes of children with T1D and children diagnosed with asthma (ICD-10 code J45), another lifelong chronic disease commonly diagnosed in childhood. We matched up to 3 children with asthma with each T1D exposed individual; we were unable to match 98 (2.15%) individuals.

Finally, we applied two analytical strategies to quantitively assess unmeasured confounding. First, we computed *E* values for each of our regression models where the 95% CI did not include a null effect, indicating the level of confounding that would be required to explain away the observed associations^17^. We considered the outcomes to be rare. Second, we performed external adjustment for unmeasured confounders to calculate how known but unmeasured confounders would influence the results^18^.

### MR analysis

#### Selection of genetic variants for the exposures and outcomes

Where we identified evidence for observational associations, we performed univariable bidirectional MR analysis on GWAS of European participants, aiming to assess the total potential causal effect of T1D on subsequent psychiatric disorders. All GWAS adjusted for age, sex and population structure. Informed consent was sought per the original GWAS protocols. We used (1) all independent (10,000 kb pairs apart, *r*^2^ < 0.001) SNPs reported to be associated with T1D at the genome-wide level (*P* < 10^−8^) (*trans* instrument), and (2) six instruments featuring SNPs located in genes (*GLIS3*, *CTSH*, *IKZF3*, *IL10*, *IL2RA* and *THEMIS*) with potentially different T1D-inducing biological mechanisms that are particularly associated with childhood-onset T1D^41^ (*cis* instruments). The *cis* instruments were included to increase specificity to childhood-onset T1D, since genetically predicted T1D liability is distributed across all ages of T1D diagnosis^19^ (Supplementary Methods). Where SNPs were not available in the outcome datasets, we located proxy SNPs using linkage disequilibrium tagging (*r*^2^ > 0.8) via LDlink^42^. Approximated *F*-statistics^43^ (beta^2^/s.e.^2^) were calculated for each T1D genetic instrument used as a measure of instrument strength. For bidirectional analyses, we used complete summary data from the T1D GWAS as the outcome.

#### Statistical analysis

The *trans* instrument was clumped for linkage disequilibrium (that is, where more than one SNP with potentially different effects is tagged by an exposure SNP and can bias results) to ensure independence. For palindromic SNPs, the forward strand was inferred where possible using allele frequency information. Alleles were harmonized based on matching alleles. Where ≥2 SNPs were available for analysis, our primary analysis method was IVW regression, with correlation adjustment for *cis* instruments. Where <2 SNPs were available for analysis, we used the Wald ratio. Where ≥2 SNPs were available for analysis, we also conducted weighted median and MR-Egger regression as secondary analyses (Supplementary Methods). The results are expressed as OR with 95% CI, representing the change in odds of outcome per s.d. increase in genetically predicted T1D risk. Since our MR analysis can be considered as confirmatory, we included *P* values, including those adjusted for multiple testing, to explore the impact of potential type II statistical error on our results^44^. We used the Holm–Bonferroni method for *P*-value adjustment^45^.

#### MR sensitivity analyses

We performed several sensitivity analyses to estimate the robustness of our MR results. Power calculations^46^ estimated the minimum detectable causal effect, given the available GWAS sample sizes. SNP heterogeneity was estimated using Cochran’s *Q* test. Horizontal pleiotropy (where an exposure SNP influences the outcome by mechanisms other than through the exposure) was estimated using the MR-Egger regression intercept and the ‘MR pleiotropy residual sum and outlier’ (MR-PRESSO) method^47^ (Supplementary Methods). Using MR-PRESSO, we performed the global test to estimate for horizontal pleiotropy and, where evident, used the method to correct the IVW estimate via outlier removal. Variability between the beta-coefficients for genetic associations with the exposure in SNP–exposure associations, which can affect MR-Egger estimates, was estimated using the *I*^2^_GX_ statistic^48^. We used the R (4.2.1)^38^ libraries TwoSampleMR (0.5.6)^49^, MendelianRandomization (0.6.0)^50^ and MRPRESSO (1.0)^47^.

### Reporting summary

Further information on research design is available in the Nature Portfolio Reporting Summary linked to this article.

## Supplementary information

Supplementary Information Supplementary Methods, Tables 1–16 and Figs. 1–46.

Reporting Summary

## Data Availability

Owing to its sensitive character, the observational data cannot be published or shared with external subjects without a permission granted by the Czech Institute of Health Information and Statistics. Data used for Mendelian randomization are available without restrictions from sources indicated in Supplementary Information. T.F. and K.M. had full access to all observational data in the study and take responsibility for the integrity of the data and the accuracy of the data analysis. B.I.P. had access to all GWAS data in the study and takes responsibility for the integrity of the data and the accuracy of the data analysis.

## Code availability

The full analytical code is available at a dedicated GitHub repository: https://github.com/tmfmnk/Psychiatric-Disorders-in-Childhood-Type-1-Diabetes/.
