# Supplementary material for: Investigating the Potential Causal Link of Childhood-Onset Type 1 Diabetes with Psychiatric Disorders in Adolescence and Early Adulthood: A Nationwide Cohort and Genome-wide Mendelian Randomization Study
Source: Nat Ment Health. Author manuscript; Available in PMC 2024 Sep 11. (PMC11383797; doi:10.1038/s44220-024-00280-8)
Supplement: Supplementary material [file EMS197945-supplement-Supplementary_material.pdf]

nature mental health

Supplementary information https://doi.org/10.1038/s44220-024-00280-8

Childhood-onset type 1 diabetes and subsequent adult psychiatric disorders: a nationwide cohort and genome-wide Mendelian randomization study

In the format provided by the authors and unedited

Table of Contents

SUPPLEMENTARY METHODS 4

OBSERVATIONAL ANALYSIS 4

Data 4

Exposure 4

Outcome 5

Sensitivity Analyses 5

MENDELIAN RANDOMIZATION (MR) ANALYSIS 7

Selection of Genetic Instruments for T1D 7

Inverse Weighted Variance (IVW) Analysis 7

Weighted Median Analysis 7

MR Egger Analysis 7

Pleiotropy Residual Sum and Outlier (MR-PRESSO) Test 8

SUPPLEMENTARY RESULTS 9

SUPPLEMENTARY TABLE 1 RISK OF OCCURRENCE OF MENTAL DISORDERS IN CHILDREN WITH T1D, SEX-STRATIFIED 9

SUPPLEMENTARY TABLE 2 DESCRIPTION OF COHORTS SENSITIVITY ANALYSIS, T1D DIAGNOSED UP TO 9 YEARS OF AGE 10

SUPPLEMENTARY TABLE 3 DESCRIPTION OF COHORTS SENSITIVITY ANALYSIS, INCIDENT CASES OF T1D 11

SUPPLEMENTARY TABLE 4 DESCRIPTION OF COHORTS SENSITIVITY ANALYSIS, ADJUSTMENT FOR THE NUMBER OF PREVIOUS HOSPITALIZATIONS 12

SUPPLEMENTARY TABLE 5 DESCRIPTION OF COHORTS SENSITIVITY ANALYSIS, COMPARISON WITH INDIVIDUALS WITH ASTHMA 13

SUPPLEMENTARY TABLE 6 E-VALUES 14

SUPPLEMENTARY TABLE 7 EXTERNAL ADJUSTMENT FOR UNMEASURED CONFOUNDING 15

SUPPLEMENTARY TABLE 8 EXPOSURES AND OUTCOMES USED IN MENDELIAN RANDOMIZATION ANALYSES AND SOURCE OF GWAS SUMMARY STATISTICS 19

SUPPLEMENTARY TABLE 9 MAIN MENDELIAN RANDOMIZATION RESULTS 20

SUPPLEMENTARY TABLE 10 BIDIRECTIONAL MENDELIAN RANDOMIZATION RESULTS 24

SUPPLEMENTARY TABLE 11 MENDELIAN RANDOMIZATION SENSITIVITY ANALYSIS, INSTRUMENT STRENGTH OF EXPOSURE SNPS 25

SUPPLEMENTARY TABLE 12 MENDELIAN RANDOMIZATION SENSITIVITY ANALYSIS, MEDIAN AND RANGE FOR INSTRUMENT STRENGTH OF EXPOSURE SNPS 28

SUPPLEMENTARY TABLE 13 MENDELIAN RANDOMIZATION SENSITIVITY ANALYSIS, TESTS OF HETEROGENEITY AND HORIZONTAL PLEIOTROPY 29

SUPPLEMENTARY TABLE 14 MENDELIAN RANDOMIZATION SENSITIVITY ANALYSIS, MR-PRESSO 31

SUPPLEMENTARY TABLE 15 MENDELIAN RANDOMIZATION SENSITIVITY ANALYSIS, TESTS FOR MEASUREMENT ERROR OF EXPOSURE SNPS 32

SUPPLEMENTARY TABLE 16 MENDELIAN RANDOMIZATION POWER CALCULATION 33

SUPPLEMENTARY FIGURE 1 FLOWCHART 34

SUPPLEMENTARY FIGURE 2 MATCHING PROCEDURE 35

SUPPLEMENTARY FIGURE 3 DIRECTED ACYCLIC GRAPH 36

SUPPLEMENTARY FIGURE 4 CUMULATIVE EVENT PLOT ON DEVELOPING SUBSTANCE USE DISORDERS IN CHILDREN WITH T1D 37

SUPPLEMENTARY FIGURE 5 CUMULATIVE EVENT PLOT ON DEVELOPING ALCOHOL USE DISORDERS IN CHILDREN WITH T1D 38

SUPPLEMENTARY FIGURE 6 CUMULATIVE EVENT PLOT ON DEVELOPING DRUG USE DISORDERS IN CHILDREN WITH T1D 39

SUPPLEMENTARY FIGURE 7 CUMULATIVE EVENT PLOT ON DEVELOPING OPIOID USE DISORDERS IN CHILDREN WITH T1D 40

SUPPLEMENTARY FIGURE 8 CUMULATIVE EVENT PLOT ON DEVELOPING CANNABIS USE DISORDERS IN CHILDREN WITH T1D 41

Supplementary Figure 9 Cumulative Event Plot on Developing Other Non-alcohol Substance Use Disorders in Children with T1D 42
Supplementary Figure 10 Cumulative Event Plot on Developing Psychotic Disorders in Children with T1D 43
Supplementary Figure 11 Cumulative Event Plot on Developing Schizophrenia in Children with T1D 44
Supplementary Figure 12 Cumulative Event Plot on Developing Other Psychotic Disorders in Children with T1D 45
Supplementary Figure 13 Cumulative Event Plot on Developing Mood Disorders in Children with T1D 46
Supplementary Figure 14 Cumulative Event Plot on Developing Bipolar Disorder in Children with T1D 47
Supplementary Figure 15 Cumulative Event Plot on Developing Depression in Children with T1D 48
Supplementary Figure 16 Cumulative Event Plot on Developing Other Mood Disorders in Children with T1D 49
Supplementary Figure 17 Cumulative Event Plot on Developing Anxiety Disorders in Children with T1D 50
Supplementary Figure 18 Cumulative Event Plot on Developing Other Anxiety Disorders in Children with T1D 51
Supplementary Figure 19 Cumulative Event Plot on Developing Panic Disorder in Children with T1D 52
Supplementary Figure 20 Cumulative Event Plot on Developing Reaction to Severe Stress and Adjustment Disorders in Children with T1D 53
Supplementary Figure 21 Cumulative Event Plot on Developing All Other Anxiety Disorders in Children with T1D 54
Supplementary Figure 22 Cumulative Event Plot on Developing Behavioural Syndromes in Children with T1D 55
Supplementary Figure 23 Cumulative Event Plot on Developing Eating Disorders in Children with T1D 56
Supplementary Figure 24 Cumulative Event Plot on Developing Anorexia Nervosa in Children with T1D 57
Supplementary Figure 25 Cumulative Event Plot on Developing Bulimia Nervosa in Children with T1D 58
Supplementary Figure 26 Cumulative Event Plot on Developing Other Eating Disorders in Children with T1D 59
Supplementary Figure 27 Cumulative Event Plot on Developing Other Behavioural Syndromes in Children with T1D 60
Supplementary Figure 28 Cumulative Event Plot on Developing Personality Disorders in Children with T1D 61
Supplementary Figure 29 Cumulative Event Plot on Developing Specific Personality Disorders in Children with T1D 62
Supplementary Figure 30 Cumulative Event Plot on Developing Other Personality Disorders in Children with T1D 63
Supplementary Figure 31 Risk of Occurrence of Psychiatric Disorders in Individuals with Childhood-onset T1D Sensitivity Analysis, T1D Diagnosed up-to 9 Years of Age 64
Supplementary Figure 32 Risk of Occurrence of Psychiatric Disorders in Individuals with Childhood-onset T1D Sensitivity Analysis, Incident Cases of T1D 65
Supplementary Figure 33 Risk of Occurrence of Psychiatric Disorders in Individuals with Childhood-onset T1D Sensitivity Analysis, Adjustment for the Number of Previous Hospitalizations 66
Supplementary Figure 34 Risk of Occurrence of Psychiatric Disorders in Individuals with Childhood-onset T1D Sensitivity Analysis, Comparison with Individuals with Asthma 67
Supplementary Figure 35 Scatterplots for MR Analyses with Evidence for an Association of T1D with Subsequent Mental Disorders, *Trans* Instrument with Schizophrenia 68
Supplementary Figure 36 Scatterplots for MR Analyses with Evidence for an Association of T1D with Subsequent Mental Disorders, *GLIS3 Cis* Instrument with Schizophrenia 69
Supplementary Figure 37 Scatterplots for MR Analyses with Evidence for an Association of T1D with Subsequent Mental Disorders, *CTSH Cis* Instrument with Schizophrenia 70

Supplementary Figure 38 Scatterplots for MR Analyses with Evidence for an Association of T1D with Subsequent Mental Disorders, *GLIS3 Cis* Instrument with Anxiety Disorders 71
Supplementary Figure 39 Scatterplots for MR Analyses with Evidence for an Association of T1D with Subsequent Mental Disorders, *GLIS3* Cis instrument with Major Depressive Disorder 72
Supplementary Figure 40 Scatterplots for MR Analyses with Evidence for an Association of T1D with Subsequent Mental Disorders, *GLIS3 Cis* Instrument with Alcohol Dependence 73
Supplementary Figure 41 Scatterplots for MR Analyses with Evidence for an Association of T1D with Subsequent Mental Disorders, *IL-2RA Cis* Instrument with Anorexia Nervosa 74
Supplementary Figure 42 Single SNP Plots for MR Analyses with Evidence for an Association of T1D with Subsequent Mental Disorders, *Trans* Instrument with Schizophrenia 75
Supplementary Figure 43 Single SNP Plots for MR Analyses with Evidence for an Association of T1D with Subsequent Mental Disorders, *GLIS3 Cis* Instrument with Schizophrenia 76
Supplementary Figure 44 Single SNP Plots for MR Analyses with Evidence for an Association of T1D with Subsequent Mental Disorders, *CTSH Cis* Instrument with Schizophrenia 77
Supplementary Figure 45 Single SNP Plots for MR Analyses with Evidence for an Association of T1D with Subsequent Mental Disorders, *GLIS3 Cis* Instrument with Anxiety Disorders 78
Supplementary Figure 46 Single SNP Plots for MR Analyses with Evidence for an Association of T1D with Subsequent Mental Disorders, *GLIS3 Cis* Instrument with Major Depressive Disorder 79

REFERENCES 80

Supplementary Methods
Observational Analysis
Data
We used individual-level, de-identified data from the Czech nationwide registers (1) of all-cause hospitalizations and (2) and all-cause deaths, covering virtually the entire Czech population (approximately 10.7 million inhabitants). Linkage is possible via a unique identifier assigned after birth included in both registers. The state-funded Institute of Health Information and Statistics (IHIS) is responsible for the maintenance and quality control of the registers. The registers are used for public health purposes and as claim database by Czech insurance companies. Complete data covering the time-period from 1st January 1994 (the earliest available) to 31st December 2017 was provided by the IHIS to the Czech National Institute of Mental Health (NIMH). The only exception are the third, fourth and fifth diagnosis codes, which were not provided for years 2016 and 2017. The present study was approved by the Ethical Committee of the NIMH (code number 182/22).

All-cause hospitalization records were generated by health professionals who routinely completed a standard, mandatory form at patient discharge [1]. Information on clinical characteristics is gathered, including the date of admission and discharge, primary and up-to five secondary diagnoses coded according to World Health Organization International Classification of Diseases, tenth edition (ICD-10). In addition, basic socio-demographic information (e.g., age and sex) are always recorded. Other sociodemographic information (marital status, occupation and region of residence) may also be recorded. Individuals before 15 years of age have the occupation coded as not working. Then, the register of all-cause deaths consists of information from death certificates that are routinely completed by physicians for all deaths occurring in Czechia [1]. Variables present in the register of all-cause deaths include the date of death, the ICD-10 cause and, if applicable, the external cause of death, age at death and sex.

Exposure
We excluded (1) records with missing information on any of the key variables (sex, age, admission and discharge date, region of residence, primary diagnosis) or with invalid admission and discharge dates, (2) records with multiple registered dates of death or hospitalizations after the date of death, and (3) overlapping hospitalizations where the admission date of a hospitalization occurred before the discharge date of another hospitalization. We applied the first two criteria to remove records affected by administrative and/or technical errors, while the third criterion to avoid severe identification problems (negative time-to-events). We included all hospitalizations that began and ended between January 1st 1994 and December 31st 2007. To mitigate the risk of loss to follow-up, we excluded individuals residing outside Czechia.

To construct the exposed cohort, we extracted all records with ICD-10 codes (E10) related to type 1 diabetes (T1D) being listed as a diagnosis. When multiple records were available for an individual, we considered the first one as the index. Then, the unexposed cohort consisted of (1) records of individuals who had no history of T1D during the examined time-period and (2) records of individuals with T1D prior the onset of T1D. Thus, an individual was allowed to be present as both as an exposed individual and as unexposed counterpart.

Neither the exposed individuals nor their unexposed counterparts were allowed to have any psychiatric disorder (ICD-10 codes F1-F6 listed on the index hospitalization as either the

primary diagnosis or one of the five secondary diagnoses: these individuals were removed from the analysis.

Outcome

For each individual, we assessed the occurrence of psychiatric disorders in the time-period following the discharge from the index hospitalization, and up to December 31st 2017. Thus, each individual was followed up for at least 10 years. We examined six diagnostic groups of psychiatric disorders (substance use disorders (F1), psychotic disorders (F2), mood disorders (F3), anxiety disorders (F4), behavioural syndromes (F5), personality disorders (F6)) and 21 specific or closely related psychiatric disorders (alcohol use disorders (F10), drug use disorders (F11-F19), opioid use disorders (F11), cannabis use disorders (F12), other non-alcohol substance use disorders (F13-F19), schizophrenia (F20), other psychotic disorders (F21-F29), bipolar disorder (F30-F31), depression (F32-F33), other mood disorders (F34-F39), other anxiety disorders (F41), panic disorder (F410), reaction to severe, and adjustment disorders (F43), all other anxiety disorders (F40 or F42 or F44-F48), eating disorders (F50), anorexia nervosa (F500-F501), bulimia nervosa (F502-F503), other eating disorders (F504-F509), other behavioural syndromes (F51-F59), specific personality disorders (F60), and other personality disorders (F61-F69)). We considered the occurrence of a psychiatric disorder when it was listed as either the primary diagnosis or one of the five secondary diagnoses. When an individual had records related to multiple psychiatric disorders, we included them for each separately. We did not consider combinations of psychiatric disorders.

Sensitivity Analyses

To investigate whether our estimates are robust and not due to design choices and/or biases commonly present in observational studies, we performed multiple sets of sensitivity analysis, interrogating the link between T1D and psychiatric disorders under different scenarios. First, to further reduce the risk of including individuals who could have a psychiatric disorder before the index hospitalization (i.e., reverse causality), we constructed cohorts with age at the index hospitalization restricted to nine or less years. The occurrence of psychiatric disorders before this age is extremely rare [2], thus, the chance of reverse causality should be reduced to a very low level. We report results for models with at least five outcomes in both the exposed and unexposed group.

Second, our cohort of children with T1D most likely consisted of both incident and prevalent cases. Since individuals who are repeatedly receiving inpatient treatment for T1D might have a higher disease severity, which could be, then, responsible for worse outcomes, we restricted the analysis to incident cases. To do so, we included only individuals for whom our data cover their entire life (i.e., individuals aged 0 years in 1994, aged 0 to 1 years in 1995 and so on). We report results for models with at least five outcomes in both the exposed and unexposed group.

Third, children with T1D might have more frequent interactions with the health-care system than their counterparts, which could lead to increased chance of receiving a diagnosis of psychiatric disorder (i.e., informative presence bias) [3]. To investigate whether accounting for informative presence bias would change the results, we calculated the number of hospitalizations in the time-period of three years prior to the index hospitalization, and matched children with T1D with their counterparts on this number of hospitalisations, in addition to matching on age, sex and year and month at discharge from index hospitalization. We categorized the number of hospitalizations such as 0, 1, 2, 3, 4, 5, 6 to 7, 8 to 9, 10 to 15, 16 to 20, 21 to 30, 31 to 40, 41 to 50, and more than 50. We matched each individual T1D with up to five counterparts. We were not able to match 2 (0.05%) individuals. We note that to be able to

assess the past histories of hospitalizations for each individual, we restricted the analysis to individuals hospitalized between January 1st 1997 and December 31st 2007. We report results for models with at least five outcomes in both the exposed and unexposed group.

We further investigated how informative presence bias might be influencing our results by comparing the outcomes of children with T1D with children diagnosed with asthma. Since both T1D and asthma are commonly diagnosed in childhood and constitute life-long, currently incurable illnesses that are associated with substantial limitations of daily life activities and frequent contact with the health-care system, the results should be less biased by differences on these factors between the groups. To do so, we matched up to three children with asthma with each exposed individual on age, sex and year at discharge from index hospitalization. We were not able to match 98 (2.15%) individuals. We note that we did not use matching on month at discharge from index hospitalization to increase the number of matched individuals, however we adjusted for it in stratified Cox proportional hazards models. We report results for models with at least five outcomes in both the exposed and unexposed group.

Next, to deal with the level of unmeasured confounding, we applied two analytical strategies. First, we computed E-values for each of our regression models. The E-values indicate what the hazard ratio would need to be for an unmeasured confounder, or set of confounders, to explain away the associations observed in the models [4]. Second, we used the expert knowledge of team members to identify factors that could be considered as confounders (see directed acyclic graph in Supplementary Figure 1) to conduct external for unmeasured confounders. Importantly, this approach can accommodate only binary confounders, thus non-binary confounders such as family income could not be included. As an initial step, we extracted from literature the associations of the identified binary confounders (i.e., asthma, caesarean section, gestational age (< 37 weeks), maternal age (>= 35 years), maternal non-smoking, parental history of mental disorders (both parents), and maternal diabetes) with (1) the exposure and with (2) the outcome. The details of included studies and the exact calculation is provided in an interactive Excel file on GitHub: https://github.com/tmfmnk/Psychiatric-Disorders-in-Childhood-Type-1-Diabetes/tree/main/External%20adjustment%20for%20unmeasured%20confounders. When available, we extracted the associations from large-scale studies that used national registry data. For some confounder-exposure or confounder-outcome pairs, information was not available, leading to an uneven number of confounders in different analyses. The results of external adjustment indicate (1) the level of confounding associated with each confounder separately and (2) the weighted average confounding across all confounders, both expressed in percentage points [5]. Larger values suggest a lower confidence in the results not being due to unaccounted confounding.

Mendelian Randomization (MR) Analysis

Selection of Genetic Instruments for T1D

The *trans* instrument features all independent (10,000kb pairs apart, $r^2<0.001$) single-nucleotide polymorphisms (SNPs) reported to be associated with T1D at genome-wide level ($p<10^{-8}$). We also included six instruments featuring SNPs located in genes (*GLIS3*, *CTSH*, *IKZF3, IL-10, IL-2RA, THEMIS*) with known T1D-inducing biological mechanisms that are particularly associated with childhood-onset T1D [6] (*cis* instruments).

GLIS3 is expressed in the pancreas [6], and is a transcriptional regulator of insulin expression [7] and required for function of mature β-cells [8]. CTSH is a lysosomal protein with actions in the Islets of Langerhans [6] and is associated with function of the innate immune system [6]. CTSH functions as an endopeptidase and can cleave the N terminus of the Toll-like receptor 3 (TLR3) protein, which is expressed in the Islets of Langerhans, increasing functionality [9]. Increased CTSH expression associated with T1D susceptibility results in increased TLR3 N terminus cleavage [10], heightened responses to viral infections, and increased release of type 1 interferon. For our *CTSH cis* instrument, we specifically used the variants rs2289702 and rs3825932, which are in low LD ($r^2 = 0.26$), and have previously been implicated in T1D aetiology by altering sensitivity of β-cells to apoptosis [11]. IKZF3 also has actions in the Islets of Langerhans and regulates B cell activation thresholds and differentiation [12] and is required for generation of high affinity plasma cells [13]. IKZF3 is associated with multiple diseases, including asthma and paediatric asthma [14]. For our *IKZF3 cis* instrument, we specifically used the variant rs921649, in which the direction of the effect is opposite in asthma to autoimmune diseases [14]. THEMIS is expressed in thymocytes and circulating T cells, and is a key signalling molecule for T-cell development and survival [15]. For our *THEMIS cis* instrument, we specifically used the variant rs13204742, which has been shown to be protective for early-diagnosed T1D and celiac disease [16] but increase risk for irritable bowel disease [17]. IL-2RA and IL-10 also have known functions in T- or B-cell biology, and may exert their T1D-risk via increasing immune infiltration, antigen recognition, and a destruction of β-cells [6].

Inverse Weighted Variance (IVW) Analysis

IVW consists of a weighted linear regression of SNP-exposure SNP-outcome effect estimates. The IVW estimate is the inverse variance weighted mean of ratio estimates from 2 or more instruments [18], and assumes that all SNPs are valid instruments or that the sum of directional bias is zero. Since the intercept is an estimate of average pleiotropic effects across IVs, in an IVW approach the intercept is fixed to 0.

Weighted Median Analysis

The weighted median is the median of the weighted empirical distribution function of individual SNP ratio estimates. This method provides a consistent effect estimate if more than 50% of the information comes from valid SNPs [19].

MR Egger Analysis

MR-Egger regression consists of a weighted linear regression similar to IVW, with the assumption that horizontal pleiotropic effects and SNP-exposure associations are uncorrelated (i.e., the InSIDE assumption is not violated [20]), therefore the intercept is not fixed. MR Egger regression provides a valid effect estimate even if all SNPs are invalid instruments but assumes that uncertainty in the SNP-exposure association estimates is negligible (the 'NOME' assumption [21]).

Pleiotropy Residual Sum and Outlier (MR-PRESSO) Test

MR-PRESSO [22] relies on a regression framework where the variants' effects on the outcome are regressed on the same variants' effects on exposure, with the slope of the regression line providing an estimate of the causal effect of the exposure on the outcome. The MR-PRESSO global test evaluates overall horizontal pleiotropy amongst all IVs in a single MR test by comparing the observed distance of all the variants to the regression line (residual sum of squares) to the expected distance under the null hypothesis of no horizontal pleiotropy. The MR-PRESSO outlier test evaluates the presence of specific horizontal pleiotropic outlier variants by using the observed and expected distributions of the tested variant. Finally, the MR-PRESSO distortion test evaluates the significance of the distortion between the causal estimate before and after removal of the horizontal pleiotropic outlier variants (detected from the outlier test of MR-PRESSO).

Supplementary Results

Supplementary Table 1 Risk of Occurrence of Mental Disorders in Children with T1D, Sex-Stratified

Outcome	Females	Males
Substance use disorders	1.25 (1.02; 1.54)	1.48 (1.27; 1.73)
Alcohol use disorders	1.42 (1.06; 1.89)	1.60 (1.33; 1.93)
Drug use disorders	0.99 (0.74; 1.32)	1.39 (1.12; 1.73)
Opioid use disorders	1.21 (0.48; 3.06)	1.70 (0.87; 3.34)
Cannabis use disorders	0.71 (0.29; 1.77)	1.71 (1.13; 2.59)
Other non-alcohol substance use disorders	1.02 (0.76; 1.37)	1.37 (1.08; 1.73)
Psychotic disorders	0.72 (0.35; 1.47)	0.45 (0.22; 0.91)
Schizophrenia	1.12 (0.48; 2.60)	0.50 (0.20; 1.23)
Other psychotic disorders	0.57 (0.23; 1.41)	0.49 (0.22; 1.12)
Mood disorders	2.24 (1.65; 3.04)	2.48 (1.66; 3.70)
Bipolar disorder	2.63 (0.98; 7.05)	NA
Depression	2.31 (1.67; 3.21)	3.24 (2.13; 4.93)
Anxiety disorders	1.48 (1.24; 1.77)	1.82 (1.47; 2.24)
Other anxiety disorders	1.57 (1.08; 2.28)	1.94 (1.22; 3.10)
Panic disorder	1.70 (0.84; 3.44)	NA
Reaction to severe stress, and adjustment disorders	1.59 (1.23; 2.05)	1.99 (1.53; 2.60)
All other anxiety disorders	1.37 (1.03; 1.82)	1.30 (0.87; 1.94)
Behavioural syndromes	4.17 (3.11; 5.60)	4.18 (2.51; 6.96)
Eating disorders	3.73 (2.62; 5.32)	NA
Anorexia nervosa	2.75 (1.69; 4.45)	NA
Bulimia nervosa	6.50 (3.23; 13.07)	NA
Other eating disorders	6.64 (3.65; 12.05)	NA
Other behavioural syndromes	5.20 (3.11; 8.72)	6.04 (3.46; 10.52)
Personality disorders	1.45 (1.00; 2.11)	1.35 (0.97; 1.86)
Specific personality disorders	1.55 (1.03; 2.35)	1.98 (1.30; 3.02)
Other personality disorders	1.32 (0.70; 2.47)	1.13 (0.75; 1.71)

NA denotes a situation when the number of outcomes in either the individuals with type 1 diabetes or their matched counterparts was less than 5. The associations between T1D and psychiatric disorders are expressed as adjusted hazard ratios with 95% confidence intervals.

Supplementary Table 2 Description of Cohorts Sensitivity Analysis, T1D Diagnosed up to 9 Years of Age

	Matched counterparts	Type 1 diabetes cohort
Total, n	23,680	2,368
Males, n (%)	12,470 (52.66)	1,247 (52.66)
Age, mean (SD)	5.51 (2.58)	5.51 (2.59)
Discharge year on index hospitalisation, median (IQR)	2001 (1997-2004)	2001 (1997-2004)
Discharge month of index hospitalisation, median (IQR)	6 (3-10)	6 (3-10)

The results are presented as absolute numbers (n) with proportions (%), means with standard deviations (SD), and medians with interquartile ranges (IQR). The distribution on sex, age, month and year at discharge is the same due to exact matching on these characteristics.

Supplementary Table 3 Description of Cohorts Sensitivity Analysis, Incident Cases of T1D

	Matched counterparts	Type 1 diabetes cohort
Total, n	17,290	1,729
Males, n (%)	9,200 (53.21)	920 (53.21)
Age, mean (SD)	5.76 (3.38)	5.76 (3.38)
Discharge year on index hospitalisation, median (IQR)	2004 (2002-2006)	2004 (2002-2006)
Discharge month of index hospitalisation, median (IQR)	6 (3-10)	6 (3-10)

The results are presented as absolute numbers (n) with proportions (%), means with standard deviations (SD), and medians with interquartile ranges (IQR). The distribution on sex, age, month and year at discharge is the same due to exact matching on these characteristics.

Supplementary Table 4 Description of Cohorts Sensitivity Analysis, Adjustment for the Number of Previous Hospitalizations

	Matched counterparts	Type 1 diabetes cohort
Total, n	19,341	3,878
Males, n (%)	10,243 (52.96)	2,053 (52.94)
Age, mean (SD)	8.75 (3.87)	8.76 (3.87)
Discharge year on index hospitalisation, median (IQR)	2002 (1999-2004)	2002 (1999-2004)
Discharge month of index hospitalisation, median (IQR)	6 (3-10)	6 (3-10)

The results are presented as absolute numbers (n) with proportions (%), means with standard deviations (SD), and medians with interquartile ranges (IQR).

Supplementary Table 5 Description of Cohorts Sensitivity Analysis, Comparison with Individuals with Asthma

	Matched counterparts	Type 1 diabetes cohort
Total, n	13,365	4,458
Males, n (%)	7,217 (54.00)	2,406 (53.97)
Age, mean (SD)	8.59 (3.89)	8.59 (3.89)
Discharge year on index hospitalisation, median (IQR)	2000 (1997-2004)	2000 (1997-2004)
Discharge month of index hospitalisation, median (IQR)	7 (4-10)	6 (3-10)

The results are presented as absolute numbers (n) with proportions (%), means with standard deviations (SD), and medians with interquartile ranges (IQR).

Supplementary Table 6 E-values

	E-value
Substance use disorders	2.14
Alcohol use disorders	2.45
Drug use disorders	1.72
Opioid use disorders	NA
Cannabis use disorders	NA
Other non-alcohol substance use disorders	NA
Psychotic disorders	3.02
Schizophrenia	NA
Other psychotic disorders	3.22
Mood disorders	4.08
Bipolar disorder	NA
Depression	4.66
Other mood disorders	NA
Anxiety disorders	2.6
Phobias	2.79
Other anxiety disorders	NA
Panic disorder	2.92
Generalized anxiety disorder	2.02
Obsessive-compulsive disorder	7.82
Reaction to severe stress, and adjustment disorders	6.4
All other anxiety disorders	4.53
Behavioural syndromes	11.86
Eating disorders	10.49
Anorexia nervosa	10.61
Bulimia nervosa	2.13
Other eating disorders	2.88
Other behavioural syndromes	NA
Personality disorders	2.14
Specific personality disorders	2.45
Other personality disorders	1.72

Children with T1D were exact matched on sex, age, discharge year and discharge month on the index hospitalization. The models were adjusted for sex, age, discharge year and discharge month at the index hospitalization. NA denotes situations when the estimates in the main analysis were consistent with a null effect.

Supplementary Table 7 External Adjustment for Unmeasured Confounding

Confounder	Exposure	Outcome	PRV confounder	ARR*	Percent bias†
Asthma	Type 1 diabetes	Alcohol use disorders	0.09	1.02	2.23
Maternal age (>= 35 years)	Type 1 diabetes	Alcohol use disorders	0.07	1.00	0.35
**Net confounding**					
**Sum of all negative biases**	**N/A**				
**Weighted average‡**	**1.36**				
**Sum of all positive biases**	**2.58**				
Caesarean section	Type 1 diabetes	Anorexia nervosa	0.11	1.00	0.20
Maternal age (>= 35 years)	Type 1 diabetes	Anorexia nervosa	0.07	1.00	0.28
**Net confounding**					
**Sum of all negative biases**	**N/A**				
**Weighted average‡**	**0.23**				
**Sum of all positive biases**	**0.48**				
Asthma	Type 1 diabetes	Anxiety disorders	0.09	1.02	1.62
Caesarean section	Type 1 diabetes	Anxiety disorders	0.11	1.00	0.27
Maternal age (>= 35 years)	Type 1 diabetes	Anxiety disorders	0.07	1.00	0.33
Maternal non-smoking	Type 1 diabetes	Anxiety disorders	0.71	0.98	-1.87
Parental history of mental disorders (both parents)	Type 1 diabetes	Anxiety disorders	0.0162	1.01	0.55
Maternal diabetes	Type 1 diabetes	Anxiety disorders	0.01	1.02	2.18
**Net confounding**					
**Sum of all negative biases**	**-1.87**				
**Weighted average‡**	**-1.10**				
**Sum of all positive biases**	**4.95**				
Caesarean section	Type 1 diabetes	Bipolar disorder	0.11	1.00	0.42
Gestational age (< 37 weeks)	Type 1 diabetes	Bipolar disorder	0.06	1.04	3.61
Maternal age (>= 35 years)	Type 1 diabetes	Bipolar disorder	0.07	1.00	0.35
**Net confounding**					

**Sum of all negative biases**	**N/A**				
**Weighted average‡**	**1.16**				
**Sum of all positive biases**	**4.38**				
Maternal age (>= 35 years)	Type 1 diabetes	Cannabis use disorders	0.07	1.00	0.28
**Net confounding**					
**Sum of all negative biases**	**N/A**				
**Weighted average‡**	**0.28**				
**Sum of all positive biases**	**0.28**				
Asthma	Type 1 diabetes	Depression	0.09	1.02	1.92
Caesarean section	Type 1 diabetes	Depression	0.11	1.00	0.24
Gestational age (< 37 weeks)	Type 1 diabetes	Depression	0.06	1.01	1.27
Maternal non-smoking	Type 1 diabetes	Depression	0.71	0.98	-2.33
**Net confounding**					
**Sum of all negative biases**	**-2.33**				
**Weighted average‡**	**-1.44**				
**Sum of all positive biases**	**3.44**				
Caesarean section	Type 1 diabetes	Eating disorders	0.11	1.00	0.00
Gestational age (< 37 weeks)	Type 1 diabetes	Eating disorders	0.06	1.02	1.61
Maternal age (>= 35 years)	Type 1 diabetes	Eating disorders	0.07	1.00	0.07
Maternal diabetes	Type 1 diabetes	Eating disorders	0.01	1.01	1.12
**Net confounding**					
**Sum of all negative biases**	**N/A**				
**Weighted average‡**	**0.44**				
**Sum of all positive biases**	**2.81**				
Maternal age (>= 35 years)	Type 1 diabetes	Mood disorders	0.07	1.00	0.31
Maternal diabetes	Type 1 diabetes	Mood disorders	0.01	1.01	1.07
Parental history of mental disorders (both parents)	Type 1 diabetes	Mood disorders	0.0162	1.00	0.46
**Net confounding**					
**Sum of all negative biases**	**N/A**				
**Weighted average‡**	**0.41**				

**Sum of all positive biases**	**1.84**				
Maternal diabetes	Type 1 diabetes	Personality disorders	0.01	1.01	1.20
Maternal non-smoking	Type 1 diabetes	Personality disorders	0.71	0.93	-7.25
Parental history of mental disorders (both parents)	Type 1 diabetes	Personality disorders	0.0162	1.01	0.88
**Net confounding**					
**Sum of all negative biases**	**-7.25**				
**Weighted average‡**	**-6.97**				
**Sum of all positive biases**	**2.08**				
Gestational age (< 37 weeks)	Type 1 diabetes	Psychotic disorders	0.06	1.01	1.37
Maternal age (>= 35 years)	Type 1 diabetes	Psychotic disorders	0.07	1.00	0.47
Maternal non-smoking	Type 1 diabetes	Psychotic disorders	0.71	0.99	-1.45
Parental history of mental disorders (both parents)	Type 1 diabetes	Psychotic disorders	0.0162	1.02	2.34
**Net confounding**					
**Sum of all negative biases**	**-1.45**				
**Weighted average‡**	**-1.02**				
**Sum of all positive biases**	**4.18**				
Caesarean section	Type 1 diabetes	Schizophrenia	0.11	1.01	0.71
Maternal age (>= 35 years)	Type 1 diabetes	Schizophrenia	0.07	1.00	0.49
Maternal diabetes	Type 1 diabetes	Schizophrenia	0.01	1.03	2.52
Parental history of mental disorders (both parents)	Type 1 diabetes	Schizophrenia	0.0162	1.03	2.74
**Net confounding**					
**Sum of all negative biases**	**N/A**				
**Weighted average‡**	**0.87**				
**Sum of all positive biases**	**6.46**				
Maternal age (>= 35 years)	Type 1 diabetes	Specific personality disorders	0.07	1.00	0.35
**Net confounding**					
**Sum of all negative biases**	**N/A**				
**Weighted average‡**	**0.35**				

**Sum of all positive biases**	**0.35**				
Maternal age (>= 35 years)	Type 1 diabetes	Substance use disorders	0.07	1.00	0.28
Maternal diabetes	Type 1 diabetes	Substance use disorders	0.01	1.00	-0.13
Parental history of mental disorders (both parents)	Type 1 diabetes	Substance use disorders	0.0162	1.02	1.73
**Net confounding**					
**Sum of all negative biases**	-0.13				
**Weighted average**‡	0.48				
**Sum of all positive biases**	2.02				

*Apparent relative risk between exposure (type 1 diabetes) and psychiatric disorders outcome if the potential confounder was not controlled for, under the assumption that the fully adjusted relative risk RR equals 1.0.

† Bias = [(ARR-RR)/RR] *100.

‡ Weighted average of net confounding equals to the sum of all component biases weighted by the population prevalence of each confounder.

Supplementary Table 8 Exposures and Outcomes Used in Mendelian Randomization Analyses and Source of GWAS Summary Statistics

Trait	Author, year (consortium)	Sample size	Cases/controls	Participant description	PubMed identifier
Exposure					
T1D	Chiou et al, 2021	520,580	18,942/501,638	European Adults	34012112
Outcome					
Alcohol dependence	Walters et al (2018) (PGC)	46,568	11,569/34,599	European Adults	30336701
Anorexia nervosa	Watson et al, 2019 (PGC)	72,517	16,992/55,525	European Adults	31308545
Major depressive disorder	Wray et al, 2018 (PGC)	480,359	135,458/344,901	European Adults	29700475
Schizophrenia	Trubetskoy et al, 2022 (PGC)	320,404	76,755/243,649	European Adults	29483656
Bipolar disorder	Mullins et al, 2021 (PGC)	413,466	41,917/371,549	European Adults	34002096
Anxiety disorder	Otowa et al, 2016 (PGC)	31,060	18,186/17,310	European Adults	26754954

Supplementary Table 9 Main Mendelian Randomization Results

Exposure	Outcome	Method	Odds ratio (95% confidence intervals)	*p*-value	Adjusted *p*-value
T1D *trans* instrument	Alcohol dependence	IVW	1.01 (0.96; 1.09)	0.600	1.000
		Weighted Median	1.03 (0.93; 1.14)	0.565	0.652
		MR Egger	0.95 (0.86; 1.05)	0.283	0.849
	Schizophrenia	IVW	0.96 (0.93; 0.99)	0.019	0.133
		Weighted Median	0.96 (0.92; 0.99)	0.032	0.096
		MR Egger	0.94 (0.89; 0.99)	0.018	0.072
	Bipolar disorder	IVW	0.98 (0.94; 1.02)	0.374	1.000
		Weighted Median	0.97 (0.92; 1.03)	0.346	0.858
		MR Egger	0.96 (0.90; 1.03)	0.319	0.992
	Major depressive disorder	IVW	0.99 (0.97; 1.02)	0.523	1.000
		Weighted Median	1.00 (0.97; 1.04)	0.897	1.000
		MR Egger	0.98 (0.94; 1.02)	0.338	1.000
	Anxiety disorders	IVW	0.94 (0.89; 1.05)	0.094	0.384
		Weighted Median	0.92 (0.84; 1.02)	0.098	0.294
		MR Egger	0.91 (0.82; 0.99)	0.032	0.128
	Anorexia nervosa	IVW	0.97 (0.94; 1.01)	0.135	0.708
		Weighted Median	0.98 (0.93; 1.03)	0.381	1.000
		MR Egger	0.95 (0.90; 1.01)	0.085	0.340
T1D *GLIS3 cis* instrument	Alcohol dependence	Corr-Adjusted IVW	0.91 (0.76; 1.34)	0.230	1.000
		Weighted Median	0.78 (0.65; 0.96)	0.017	0.068
		MR Egger	0.93 (0.61; 1.42)	0.745	0.849
	Schizophrenia	Corr-Adjusted IVW	1.00 (0.98; 1.02)	0.991	1.000
		Weighted Median	1.10 (1.03; 1.19)	0.007	0.028
		MR Egger	1.02 (0.87; 1.19)	0.849	1.000
	Bipolar disorder	Corr-Adjusted IVW	1.05 (0.96; 1.18)	0.317	1.000
		Weighted Median	1.05 (0.94; 1.18)	0.369	0.858

		MR Egger	0.98 (0.77; 1.27)	0.882	1.000
	Major depressive disorder	Corr-Adjusted IVW	0.99 (0.98; 1.01)	0.146	1.000
		Weighted Median	1.07 (1.00; 1.15)	0.032	0.128
		MR Egger	1.01 (0.87; 1.17)	0.896	1.000
	Anxiety disorders	Corr-Adjusted IVW	1.46 (1.22; 1.75)	< 0.001	< 0.001
		Weighted Median	1.39 (1.11; 1.74)	0.004	0.016
		MR Egger	1.02 (0.62; 1.67)	0.933	1.000
	Anorexia nervosa	Corr-Adjusted IVW	1.00 (0.98; 1.03)	0.999	1.000
		Weighted Median	1.00 (0.89; 1.12)	0.994	1.000
		MR Egger	1.11 (0.88; 1.38)	0.394	1.000
T1D *CTSH cis* instrument	Alcohol dependence	Corr-Adjusted IVW	0.74 (0.41; 1.36)	0.337	1.000
	Schizophrenia	Corr-Adjusted IVW	0.82 (0.68; 0.99)	0.042	0.252
	Bipolar Disorder	Corr-Adjusted IVW	0.93 (0.67; 1.28)	0.660	1.000
	Major depressive disorder	Corr-Adjusted IVW	1.02 (0.85; 1.23)	0.811	1.000
	Anxiety Disorders	Corr-Adjusted IVW	0.52 (0.23; 1.20)	0.124	0.620
	Anorexia Nervosa	Corr-Adjusted IVW	0.97 (0.68; 1.40)	0.890	1.000
T1D *IKZF3 cis* instrument	Alcohol dependence	Wald Ratio	0.75 (0.36; 1.56)	0.448	1.000
	Schizophrenia	Wald Ratio	0.83 (0.63; 1.10)	0.205	1.000
	Bipolar Disorder	Wald Ratio	0.68 (0.43; 1.05)	0.084	0.504
	Major depressive disorder	Wald Ratio	0.91 (0.70; 1.18)	0.482	1.000
	Anxiety Disorders	Wald Ratio	0.69 (0.29; 1.65)	0.405	1.000
	Anorexia Nervosa	Wald Ratio	1.53 (0.98; 2.38)	0.059	1.000
T1D *THEMIS cis* instrument	Alcohol dependence	Wald Ratio	2.29 (0.61; 8.65)	0.218	1.000
	Schizophrenia	Wald Ratio	0.88 (0.55; 1.40)	0.586	1.000
	Bipolar Disorder	Wald Ratio	0.98 (0.48; 2.01)	0.959	1.000
	Major depressive disorder	Wald Ratio	0.97 (0.64; 1.49)	0.902	1.000
	Anxiety Disorders	Wald Ratio	1.11 (0.27; 4.59)	0.887	1.000
	Anorexia Nervosa	Wald Ratio	1.79 (0.86; 3.71)	0.118	0.708
T1D *IL-2RA cis* instrument	Alcohol dependence	Corr-Adjusted IVW	0.80 (0.55; 1.17)	0.257	1.000
		Weighted Median	0.87 (0.75; 1.00)	0.053	0.159
		MR Egger	0.90 (0.66; 1.21)	0.049	0.196

	Schizophrenia	Corr-Adjusted IVW	0.93 (0.80; 1.07)	0.292	1.000
		Weighted Median	0.96 (0.90; 1.01)	0.107	0.214
		MR Egger	0.95 (0.82; 0.383)	0.383	1.000
	Bipolar disorder	Corr-Adjusted IVW	0.95 (0.77; 1.17)	0.620	1.000
		Weighted Median	0.94 (0.86; 1.02)	0.133	0.532
		MR Egger	0.89 (0.75; 1.07)	0.248	0.992
	Major depressive disorder	Corr-Adjusted IVW	0.95 (0.84; 1.08)	0.436	1.000
		Weighted Median	0.96 (0.92; 1.02)	0.186	0.558
		MR Egger	0.92 (0.82; 1.02)	0.181	0.724
	Anxiety disorders	Corr-Adjusted IVW	0.83 (0.54; 1.27)	0.394	1.000
		Weighted Median	0.87 (0.73; 1.03)	0.109	0.294
		MR Egger	0.85 (0.58; 1.23)	0.395	1.000
	Anorexia nervosa	Corr-Adjusted IVW	0.89 (0.72; 1.11)	0.319	1.000
		Weighted Median	0.88 (0.81; 0.96)	0.003	0.012
		MR Egger	0.80 (0.67; 0.96)	0.665	1.000
T1D *IL-10 cis* instrument	Alcohol dependence	Corr-Adjusted IVW	1.18 (0.69; 2.01)	0.541	1.000
		Weighted Median	1.19 (0.84; 1.70)	0.326	0.652
		MR Egger	2.32 (0.59; 9.11)	0.352	0.849
	Schizophrenia	Corr-Adjusted IVW	1.01 (0.82; 1.25)	0.913	1.000
		Weighted Median	1.02 (0.88; 1.62)	0.871	0.871
		MR Egger	1.06 (0.68; 1.64)	0.833	1.000
	Bipolar disorder	Corr-Adjusted IVW	1.13 (0.81; 1.57)	0.469	1.000
		Weighted Median	1.13 (0.90; 1.41)	0.286	0.858
		MR Egger	0.97 (0.49; 1.95)	0.945	1.000
	Major depressive disorder	Corr-Adjusted IVW	0.98 (0.91; 1.18)	0.828	1.000
		Weighted Median	0.98 (0.86; 1.12)	0.760	1.000
		MR Egger	1.00 (0.67; 1.49)	0.999	1.000
	Anxiety disorders	Corr-Adjusted IVW	0.85 (0.47; 1.64)	0.679	1.000
		Weighted Median	0.86 (0.55; 1.32)	0.487	0.487
		MR Egger	0.79 (0.20; 3.05)	0.761	1.000
	Anorexia nervosa	Corr-Adjusted IVW	1.10 (0.80; 1.51)	0.554	1.000

		Weighted Median	1.10 (0.89; 1.37)	0.384	1.000
		MR Egger	0.97 (0.49; 1.89)	0.929	1.000

We used IVW OR for *trans* instrument, correlation-adjusted IVW OR for *cis* instruments with ≥2 SNPs, or Wald ratio for *cis* instruments with <2 SNPs. The estimated effects represent the change in odds of outcome per standard deviation increase in genetically-predicted type 1 diabetes risk, and are accompanied by 95% confidence intervals. Adjusted *p*-values were computed using the Holm-Bonferroni method per T1D instrument.

Supplementary Table 10 Bidirectional Mendelian Randomization Results

Outcome	Exposure	Method	Odds ratio (95% confidence interval)	*p*-value	Adjusted *p*-value
T1D	Alcohol dependence	IVW	1.06 (0.09; 12.43)	0.966	1.000
		Weighted Median	0.71 (0.14; 3.61)	0.682	1.000
		MR Egger	0.58 (0.01; 48.43)	0.818	1.000
	Schizophrenia	IVW	1.01 (0.94; 1.10)	0.720	1.000
		Weighted Median	1.10 (0.90; 1.31)	0.554	1.000
		MR Egger	1.05 (0.76; 1.44)	0.773	1.000
	Bipolar disorder	IVW	0.81 (0.66; 1.32)	0.476	1.000
		Weighted Median	0.51 (0.22; 1.99)	0.813	1.000
		MR Egger	0.51 (0.03; 7.45)	0.632	1.000
	Major depressive disorder	IVW	0.91 (0.25; 3.21)	0.877	1.000
		Weighted Median	1.36 (0.45; 4.15)	0.583	1.000
		MR Egger	27.12 (0.03; 21822.30)	0.434	1.000
	Anxiety disorders	Wald Ratio	1.41 (0.76; 2.10)	0.331	1.000
	Anorexia nervosa	IVW	0.85 (0.56; 1.28)	0.424	1.000
		Weighted Median	0.77 (0.59; 1.01)	0.054	1.000
		MR Egger	0.25 (0.01; 14.22)	0.570	1.000

The estimated effects represent the change in odds of outcome per standard deviation increase in genetically-predicted type 1 diabetes risk, and are accompanied by 95% confidence intervals. Adjusted *p*-values were computed using the Holm-Bonferroni method per T1D instrument.

Supplementary Table 11 Mendelian Randomization Sensitivity Analysis, Instrument Strength of Exposure SNPs

Exposure	SNP	F-statistic
T1D *trans* instrument	rs10751776	1.0430225
	rs574384	16.1718423
	rs12742756	21.3697067
	rs855330	3.48560679
	rs6679677	216.512973
	rs2493411	3.18264576
	rs10801128	2.28298669
	rs17623914	17.0999274
	rs3024493	32.3913714
	rs12128789	3.41508722
	rs1881146	19.0546373
	rs55893453	1.75626409
	rs12464462	25.3631
	rs4490209	20.7644703
	rs2111485	7.71176277
	rs6434435	22.4341981
	rs3087243	84.0075231
	rs13018977	2.10019595
	rs7668577	2.17698755
	rs13147049	32.2865718
	rs2611211	29.4138542
	rs12644686	18.2527022
	rs2303137	24.1105248
	rs114378220	4.74888114
	rs2188962	1.13758219
	rs1050979	4.20396487
	rs55969931	48.7600293
	rs72838204	36.6987786
	rs2395471	333.310539
	rs12665124	16.111099
	rs2523679	225.137914
	rs1008438	233.345052
	rs74999184	494.748587
	rs112647257	110.949161
	rs6908626	18.3734098
	rs9385401	6.0213624
	rs4548024	20.8560102
	rs10224046	1.41237154
	rs17323934	31.2147574
	rs7776597	7.71241077
	rs7795896	34.8937708
	rs1947178	21.9119523
	rs13259300	25.7462936
	rs3802214	18.1594255

	rs1574285	42.4588501
	rs12257077	6.49021946
	rs61839660	66.913975
	rs41295159	18.7683683
	rs722988	1.34216662
	rs78325861	16.8888327
	rs7068821	47.3875523
	rs114278107	26.6656459
	rs7110099	249.638708
	rs663743	27.0432039
	rs7936434	0.96494239
	rs607703	2.28336352
	rs1701704	47.9604934
	rs3184504	108.966886
	rs238265	23.6528511
	rs9517712	25.3505196
	rs17106304	5.18911005
	rs1350275	24.2112875
	rs56994090	43.5920639
	rs34593439	33.7128249
	rs12927355	76.2753758
	rs231972	8.85011362
	rs55993634	13.0170569
	rs8046043	21.5641109
	rs61759532	3.61083679
	rs35327136	21.7497297
	rs57209021	2.03499758
	rs7237497	57.4861253
	rs1808094	33.0176292
	rs34536443	34.7968088
	rs113374757	31.0081303
	rs601338	7.64301639
	rs202535	29.4257127
	rs11203203	11.368962
	rs4820827	43.3724974
	rs2543537	23.8205532
	rs229527	3.88457801
	rs4380994	41.1271379
	rs3892354	40.9239031
	rs1574285	42.4588501
	rs10974435	41.6478097
	rs10758591	41.1806829
	rs7024686	40.2979255
	rs7041847	41.5877095
	rs7034200	7.59917835
	rs10814914	42.1538184
	rs10116772	7.26763225
	rs10814915	41.0615725

T1D *GLIS3 cis* instrument	rs6476839	7.15916111
	rs6476842	36.4987741
	rs7020673	7.08472301
	rs10974438	3.2206397
	rs10758593	7.40761893
	rs7867224	40.6413218
	rs10814916	4.61836911
	rs34706136	0.16664369
	rs4339696	5.98073658
	rs10814917	40.2454297
T1D *CTSH cis* instrument	rs2289702	33.1984623
	rs12603332	17.2524004
T1D *IKZF3 cis* instrument	rs921649	17.86758
T1D *IL-2RA cis* instrument	rs12722563	55.1592391
	rs12722558	55.4236431
	rs12722552	55.4973496
	rs12722522	65.4494793
	rs12722508	65.9075605
	rs7909519	66.0780958
	rs61839660	66.913975
	rs12722496	66.9366014
	rs12722495	66.9079866
	rs41295049	59.1040382
	rs41295061	58.5218034
	rs41295065	58.4077133
	rs35285258	31.2737184
	rs11594656	31.3017501
	rs6602437	8.97159278
T1D *IL-10 cis* instrument	rs3024505	31.845647
	rs3024495	31.892844
	rs3024493	32.3913714
	rs3122605	5.50728765
T1D *THEMIS cis* instrument	rs13204742	7.046297

Supplementary Table 12 Mendelian Randomization Sensitivity Analysis, Median and Range for Instrument Strength of Exposure SNPs

Exposure	Median f-statistic	Range of f-statistics
T1D t*rans* instrument	21.74	0.96-494.75
T1D *GLIS3 cis i*nstrument	40.25	0.17-42.45
T1D *CTSH cis* instrument	25.22	17.25-33.19
T1D *IZKF3 cis* instrument	17.86758	17.86758
T1D *IL-2RA cis* instrument	58.28	8.97-66.94
T1D *IL-10 cis* instrument	31.87	5.51-32.39
T1D *THEMIS cis* instrument	7.046297	7.046297

For *IKZF3* and *THEMIS cis* instruments, the range is the same as the median as <2 SNPs.

Supplementary Table 13 Mendelian Randomization Sensitivity Analysis, Tests of Heterogeneity and Horizontal Pleiotropy

Exposure	Outcome	IVW*		MR Egger			
		Cochran's Q (df)	*p*-value	Cochran's Q (df)	*p*-value	MR Egger intercept (SE)	Intercept *p*-value
T1D *trans* instrument	Alcohol dependence	81.10 (66)	0.085	76.81 (65)	0.130	0.01 (0.01)	0.062
	Schizophrenia	222.00 (66)	<0.001	217.70 (65)	<0.001	0.01 (0.01)	0.261
	Bipolar disorder	19.90 (66)	1.000	19.54 (65)	1.000	0.00 (0.00)	0.552
	Major depressive disorder	15.14 (66)	1.000	14.61 (65)	1.000	0.00 (0.00)	0.468
	Anxiety disorders	53.58 (66)	0.868	51.75 (65)	0.883	0.01 (0.01)	0.207
	Anorexia nervosa	106.32 (66)	0.002	104.73 (65)	0.002	0.00 (0.00)	0.320
T1D *GLIS3 cis* instrument	Alcohol dependence	2.33 (19)	0.999	1.64 (18)	0.999	-0.01 (0.02)	0.419
	Schizophrenia	35.05 (19)	0.014	2.30 (18)	1.000	0.01 (0.01)	0.321
	Bipolar disorder	11.07 (19)	0.921	0.27 (18)	1.000	0.01 (0.01)	0.590
	Major depressive disorder	23.88 (19)	0.201	0.14 (18)	1.000	0.01 (0.01)	0.317
	Anxiety disorders	44.56 (19)	<0.001	1.84 (18)	1.000	0.02 (0.02)	0.149
	Anorexia nervosa	14.63 (19)	0.999	5.60 (18)	0.986	-0.01 (0.01)	0.196
T1D *CTSH cis* instrument	Alcohol dependence	0.29 (1)	0.589	-	-	-	-
	Schizophrenia	0.22 (1)	0.642	-	-	-	-
	Bipolar disorder	0.03 (1)	0.854	-	-	-	-
	Major depressive disorder	0.27 (1)	0.602	-	-	-	-
	Anxiety disorders	0.33 (1)	0.564	-	-	-	-
	Anorexia nervosa	0.05 (10	0.816	-	-	-	-
T1D *IL-2RA cis* instrument	Alcohol dependence	1.51 (11)	0.999	1.46 (10)	0.999	-0.01 (0.03)	0.841
	Schizophrenia	3.31 (11)	0.986	3.29 (10)	0.974	0.00 (0.00)	0.905
	Bipolar disorder	0.50 (11)	0.999	0.06 (10)	0.999	0.01 (0.02)	0.529
	Major depressive disorder	1.13 (11)	0.999	0.16 (10)	0.999	0.01 (0.01)	0.348
	Anxiety disorders	5.71 (11)	0.892	5.70 (10)	0.840	0.00 (0.03)	0.953
	Anorexia nervosa	7.24 (11)	0.780	6.26 (10)	0.793	0.02 (0.02)	0.346
	Alcohol dependence	1.04 (3)	0.792	0.01 (2)	0.997	-0.07 (0.07)	0.416
	Schizophrenia	0.04 (3)	0.998	0.01 (2)	0.999	0.00 (0.02)	0.855

T1D *IL-10 cis* instrument	Bipolar disorder	0.21 (3)	0.975	0.00 (2)	0.999	0.02 (0.04)	0.689
	Major depressive disorder	0.01 (3)	0.999	<0.001 (2)	0.999	0.00 (0.02)	0.922
	Anxiety disorders	0.82 (3)	0.846	0.67 (2)	0.665	-0.01 (0.07)	0.983
	Anorexia nervosa	0.18 (3)	0.981	0.00 (2)	0.999	0.01 (0.03)	0.713

*IVW for *trans* instrument, correlation-adjusted IVW for *cis* instruments.
IKZF3, and *THEMIS cis* instruments not included as <2 SNPs. *CTSH cis* instrument sensitivity analyses limited as 2 SNPs only.

Supplementary Table 14 Mendelian Randomization Sensitivity Analysis, MR-PRESSO

Exposure	Outcome	MR-PRESSO global test		Outlier-corrected IVW		Distortion test	
		RSS	*p*-value	β (SE)	*p*-value	Coef.	*p*-value
T1D *trans* instrument	Alcohol dependence	99.86	0.073	-	-	-	-
	Schizophrenia	249.10	<0.001	-0.021	0.032	-63.53	0.155
	Bipolar disorder	25.79	1.000	-	-	-	-
	Major depressive disorder	19.73	1.000	-	-	-	-
	Anxiety disorders	65.76	0.889	-	-	-	-
	Anorexia nervosa	126.80	0.001	-0.03 (0.02)	0.029	18.3	0.774
T1D *GLIS3 cis* instrument	Alcohol dependence	3.18	1.000	-	-	-	-
	Schizophrenia	2.74	1.000	-	-	-	-
	Bipolar disorder	0.70	1.000	-	-	-	-
	Major depressive disorder	1.60	1.000	-	-	-	-
	Anxiety disorders	6.21	0.999	-	-	-	-
	Anorexia nervosa	8.53	0.994	-	-	-	-
T1D *IL-2RA cis* instrument	Alcohol dependence	2.15	1.000	-	-	-	-
	Schizophrenia	5.67	0.986	-	-	-	-
	Bipolar disorder	0.66	1.000	-	-	-	-
	Major depressive disorder	1.85	1.000	-	-	-	-
	Anxiety disorders	10.06	0.851	-	-	-	-
	Anorexia nervosa	10.04	0.855	-	-	-	-
T1D *IL-10 cis* instrument	Alcohol dependence	1.15	0.880	-	-	-	-
	Schizophrenia	0.05	0.999	-	-	-	-
	Bipolar disorder	0.25	0.987	-	-	-	-
	Major depressive disorder	0.01	1.000	-	-	-	-
	Anxiety disorders	1.66	0.829	-	-	-	-
	Anorexia nervosa	0.21	0.988	-	-	-	-

CTSH, IKZF3 and *THEMIS cis* instruments not included as ≤2 SNPs.

Supplementary Table 15 Mendelian Randomization Sensitivity Analysis, Tests for Measurement Error of Exposure SNPs

Exposure	I^2_{GX} of SNP-exposure associations
T1D *trans* instrument	0.947
T1D *GLIS3 cis* instrument	0.738
T1D *CTSH cis* instrument	0.922
T1D *IL-2RA cis* instrument	0.883
T1D *IL-10 cis* instrument	0.597

IKZF3 and *THEMIS cis* instruments not included as <2 SNPs.

Supplementary Table 16 Mendelian Randomization Power Calculation

Outcome	Variance explained (R^2) by the instrument	Minimum causal effect (odds ratio) detectable with 80% power
Schizophrenia	0.010	0.89-1.11
	0.025	0.92-1.08
	0.050	0.94-1.06
	0.075	0.95-1.05
	0.100	0.96-1.04
Bipolar disorder	0.010	0.77-1.29
	0.025	0.85-1.18
	0.050	0.89-1.12
	0.075	0.91-1.10
	0.100	0.92-1.09
Major depressive disorder	0.010	0.91-1.10
	0.025	0.94-1.06
	0.050	0.96-1.05
	0.075	0.96-1.04
	0.100	0.97-1.03
Anxiety disorders	0.010	0.71-1.41
	0.025	0.82-1.24
	0.050	0.85-1.17
	0.075	0.88-1.14
	0.100	0.89-1.12
Anorexia nervosa	0.010	0.78-1.28
	0.025	0.85-1.18
	0.050	0.89-1.12
	0.075	0.91-1.10
	0.100	0.92-1.09
Alcohol dependence	0.010	0.74-1.35
	0.025	0.82-1.21
	0.050	0.87-1.15
	0.075	0.89-1.12
	0.100	0.90-1.10

R^2 not reported and could not be approximated (see below), so range of possible R^2 values are shown. Power calculations were calculated from Burgess *et al.* [23]. Where possible, R^2 was taken from the original GWAS. Where this was not reported, R^2 was approximated using the formula 2*beta_X^2*MAF* (1-MAF), where beta_X is the genetic association with the instrument (in SDs). Causal effect refers to the log odds in SD.

Supplementary Figure 1 Flowchart

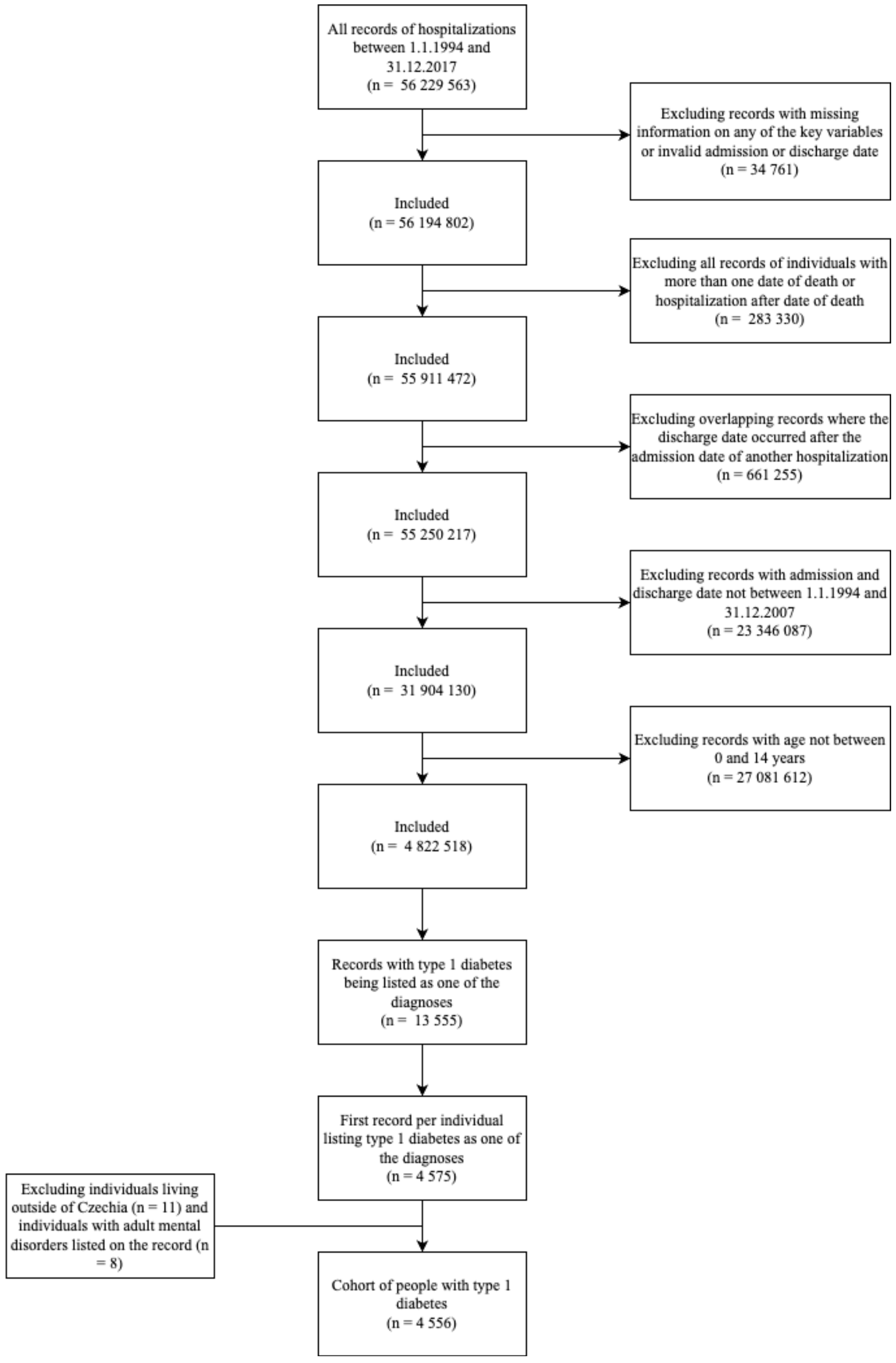

Supplementary Figure 2 Matching Procedure

Example 1 Matching of individual with type 1 diabetes with counterpart who did not develop type 1 diabetes up to that point

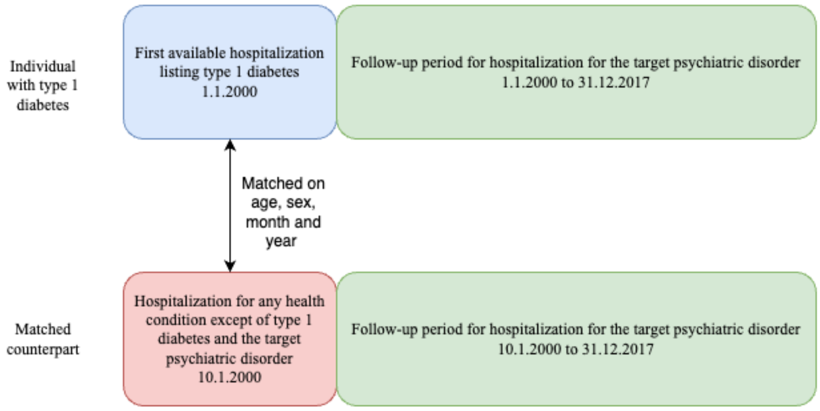

Example 2 Matching of individual with type 1 diabetes with counterpart who had no history of type 1 type diabetes up to that point and matching of the same exposed individual on their pre-exposure record with another exposed individual

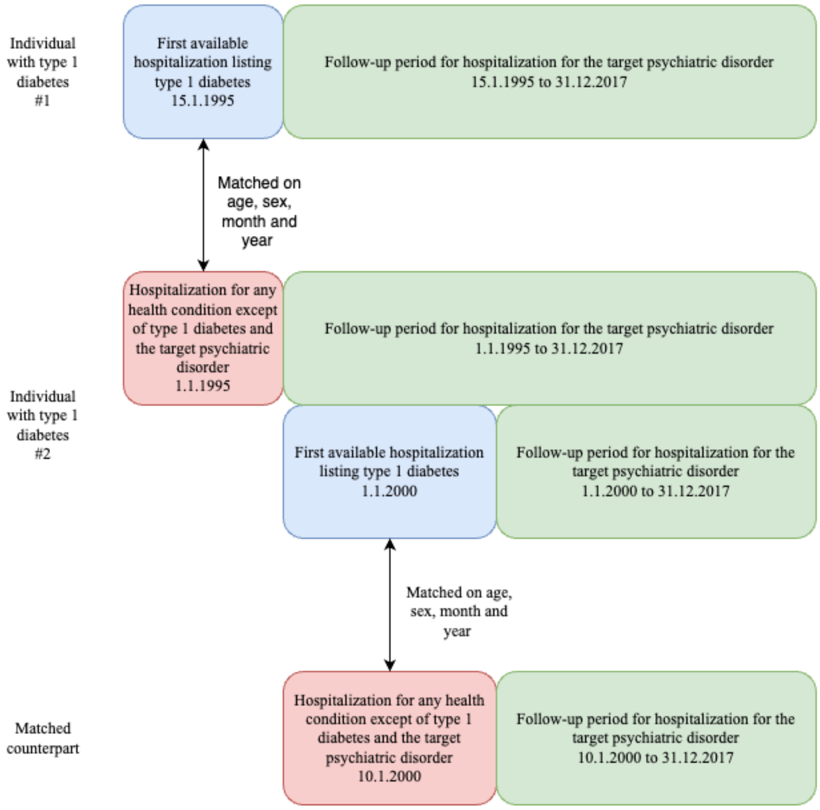

Supplementary Figure 3 Directed Acyclic Graph

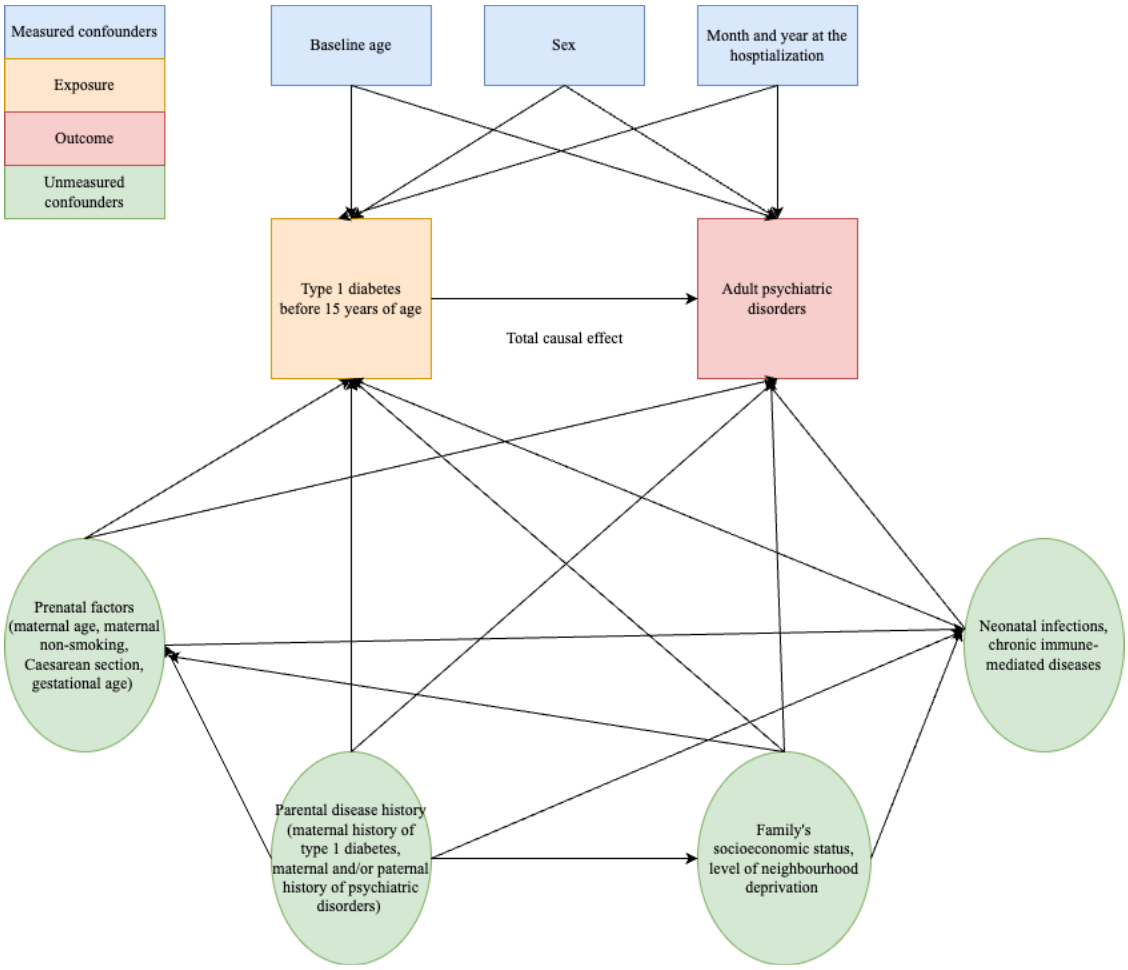

Supplementary Figure 4 Cumulative Event Plot on Developing Substance Use Disorders in Children with T1D

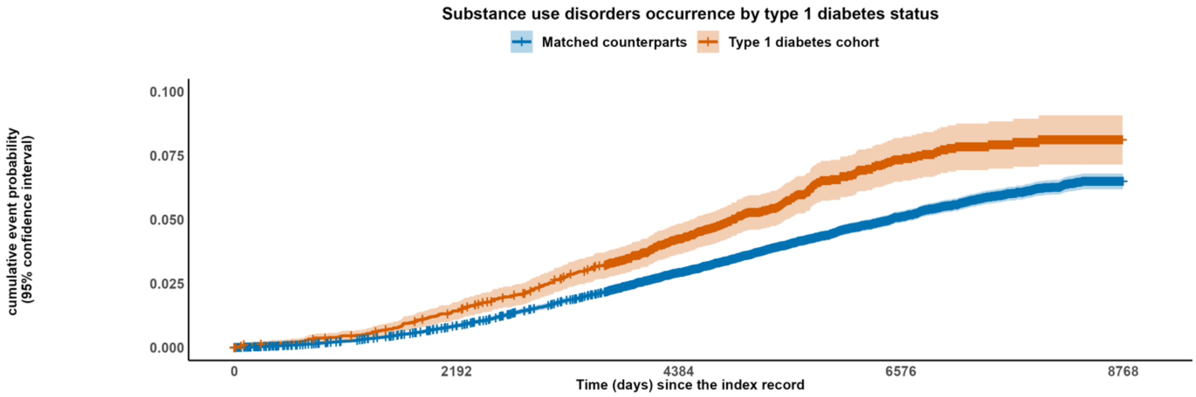

Number at risk

	0	2192	4384	6576	8768
Matched counterparts	45560	45011	38115	19355	0
Type 1 diabetes cohort	4556	4473	3745	1876	0

Time (days) since the index record

Cumulative number of censoring

	0	2192	4384	6576	8768
Matched counterparts	26	159	6147	24253	43416
Type 1 diabetes cohort	2	18	623	2401	4263

Time (days) since the index record

Cumulative number of events

	0	2192	4384	6576	8768
Matched counterparts	1	390	1305	1956	2144
Type 1 diabetes cohort	0	65	189	281	293

Time (days) since the index record

The curves in the plot depict the cumulative event probability per exposure status, accompanied by error bars expressed as 95% confidence intervals. The tables in the plot refer to the number of at risk individuals, cumulative number of censored individuals, and cumulative number of individuals who had the outcome per exposure status, respectively.

Supplementary Figure 5 Cumulative Event Plot on Developing Alcohol Use Disorders in Children with T1D

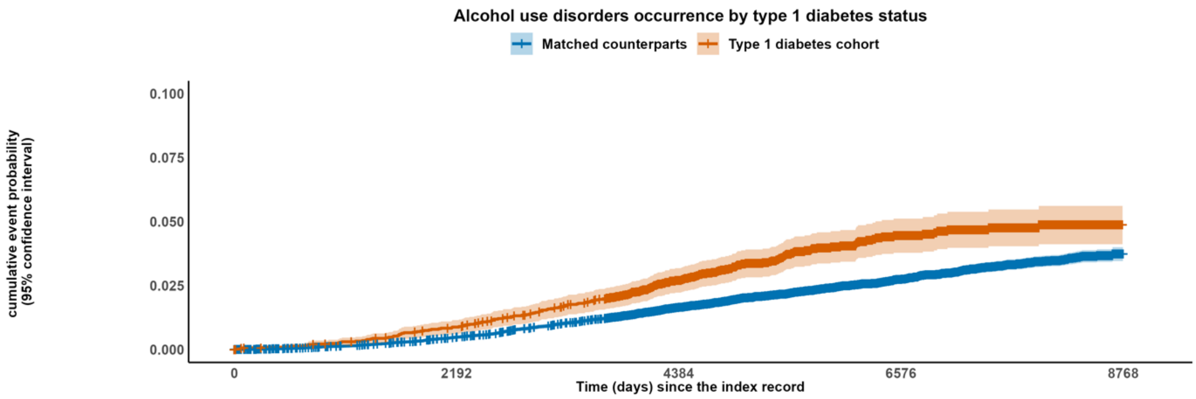

Number at risk

	0	2192	4384	6576	8768
Matched counterparts	45560	45181	38601	19806	0
Type 1 diabetes cohort	4556	4497	3797	1926	0

Time (days) since the index record

Cumulative number of censoring

	0	2192	4384	6576	8768
Matched counterparts	26	159	6231	24696	44377
Type 1 diabetes cohort	2	19	640	2459	4377

Time (days) since the index record

Cumulative number of events

	0	2192	4384	6576	8768
Matched counterparts	0	220	735	1062	1183
Type 1 diabetes cohort	0	40	120	173	179

Time (days) since the index record

The curves in the plot depict the cumulative event probability per exposure status, accompanied by error bars expressed as 95% confidence intervals. The tables in the plot refer to the number of at risk individuals, cumulative number of censored individuals, and cumulative number of individuals who had the outcome per exposure status, respectively.

Supplementary Figure 6 Cumulative Event Plot on Developing Drug Use Disorders in Children with T1D

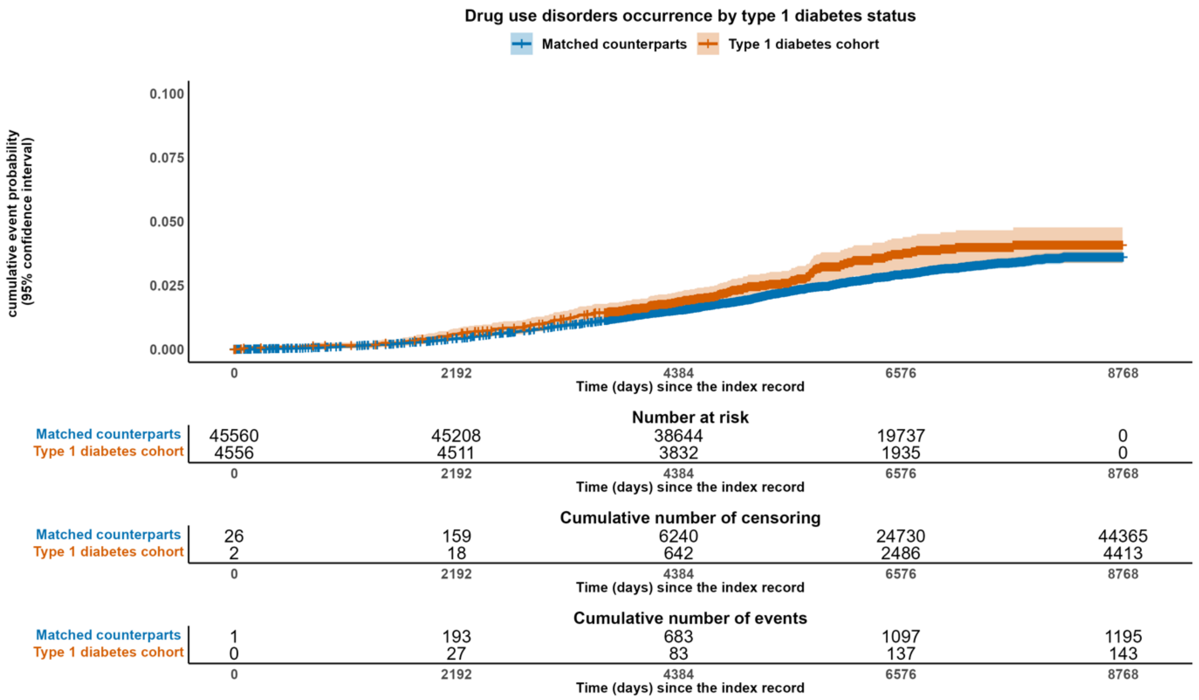

The curves in the plot depict the cumulative event probability per exposure status, accompanied by error bars expressed as 95% confidence intervals. The tables in the plot refer to the number of at risk individuals, cumulative number of censored individuals, and cumulative number of individuals who had the outcome per exposure status, respectively.

Supplementary Figure 7 Cumulative Event Plot on Developing Opioid Use Disorders in Children with T1D

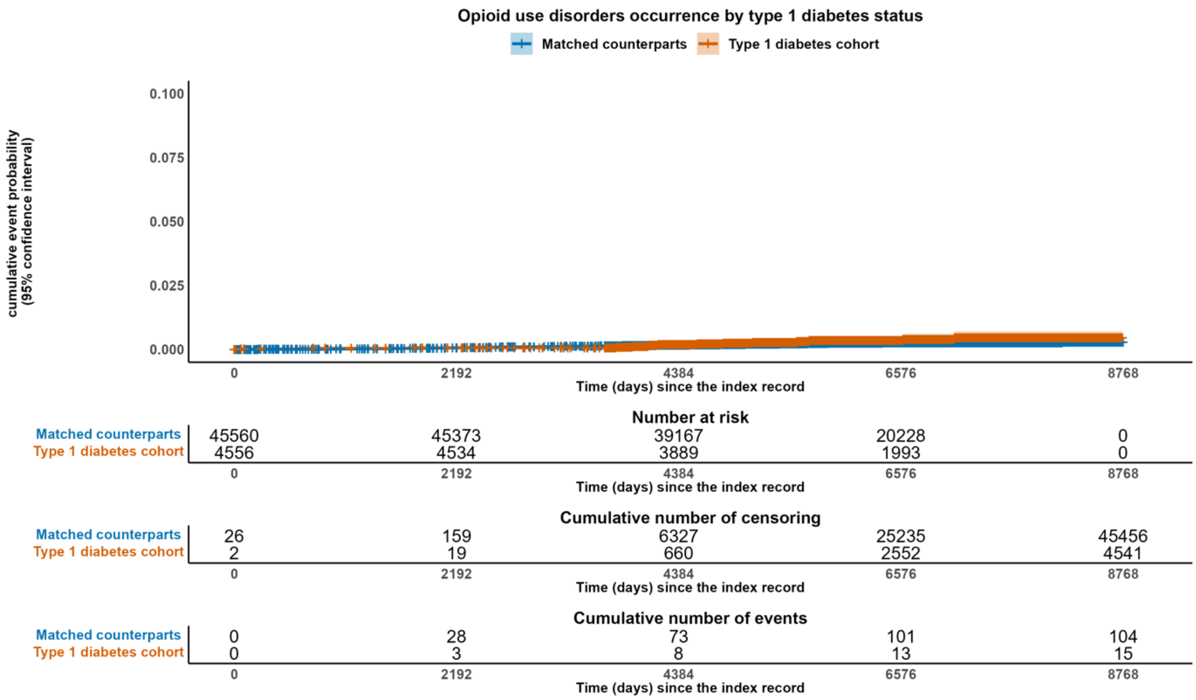

The curves in the plot depict the cumulative event probability per exposure status, accompanied by error bars expressed as 95% confidence intervals. The tables in the plot refer to the number of at risk individuals, cumulative number of censored individuals, and cumulative number of individuals who had the outcome per exposure status, respectively.

Supplementary Figure 8 Cumulative Event Plot on Developing Cannabis Use Disorders in Children with T1D

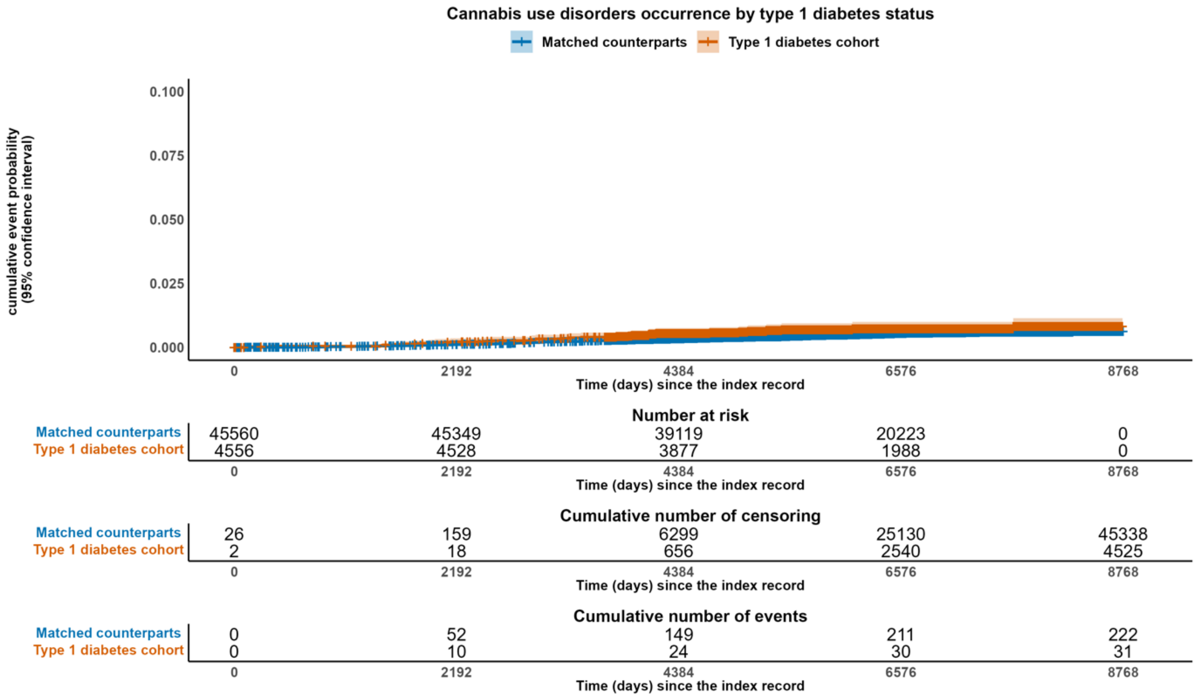

The curves in the plot depict the cumulative event probability per exposure status, accompanied by error bars expressed as 95% confidence intervals. The tables in the plot refer to the number of at risk individuals, cumulative number of censored individuals, and cumulative number of individuals who had the outcome per exposure status, respectively.

Supplementary Figure 9 Cumulative Event Plot on Developing Other Non-alcohol Substance Use Disorders in Children with T1D

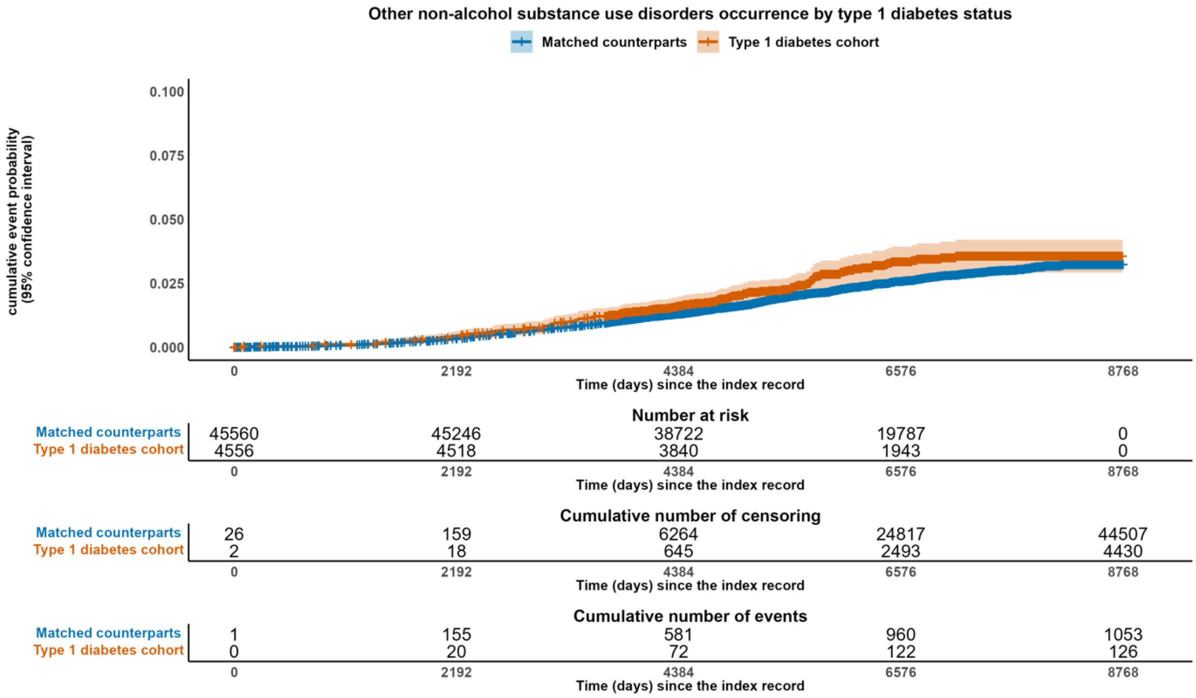

The curves in the plot depict the cumulative event probability per exposure status, accompanied by error bars expressed as 95% confidence intervals. The tables in the plot refer to the number of at risk individuals, cumulative number of censored individuals, and cumulative number of individuals who had the outcome per exposure status, respectively.

Supplementary Figure 10 Cumulative Event Plot on Developing Psychotic Disorders in Children with T1D

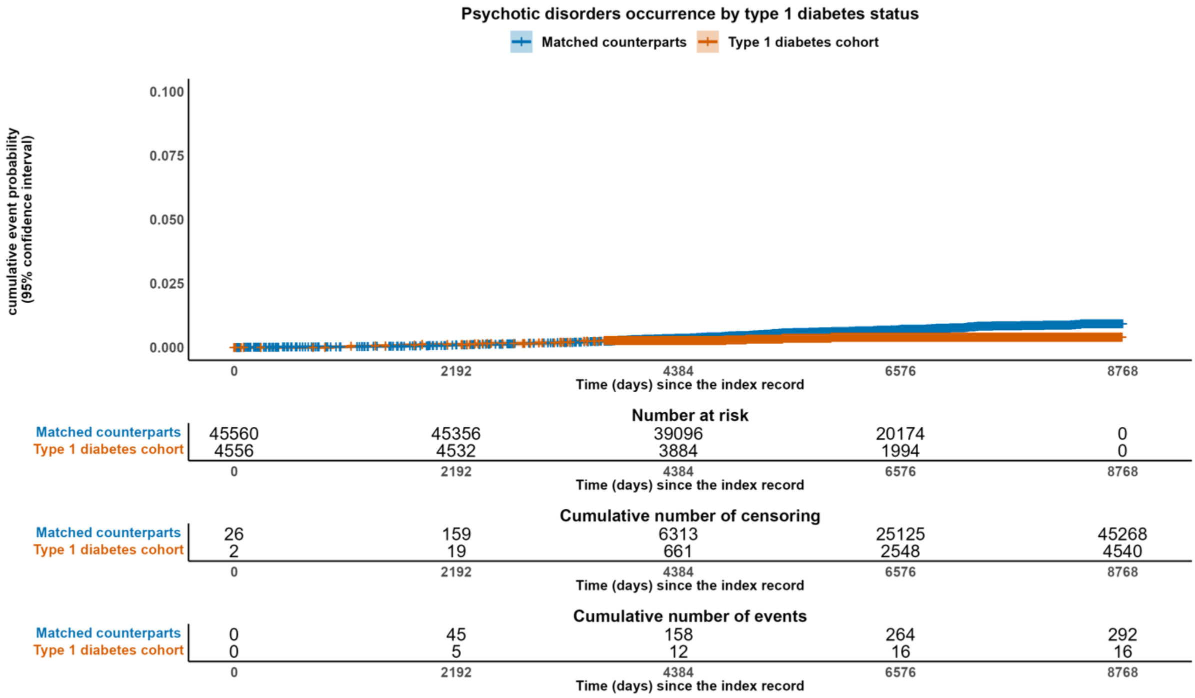

The curves in the plot depict the cumulative event probability per exposure status, accompanied by error bars expressed as 95% confidence intervals. The tables in the plot refer to the number of at risk individuals, cumulative number of censored individuals, and cumulative number of individuals who had the outcome per exposure status, respectively.

Supplementary Figure 11 Cumulative Event Plot on Developing Schizophrenia in Children with T1D

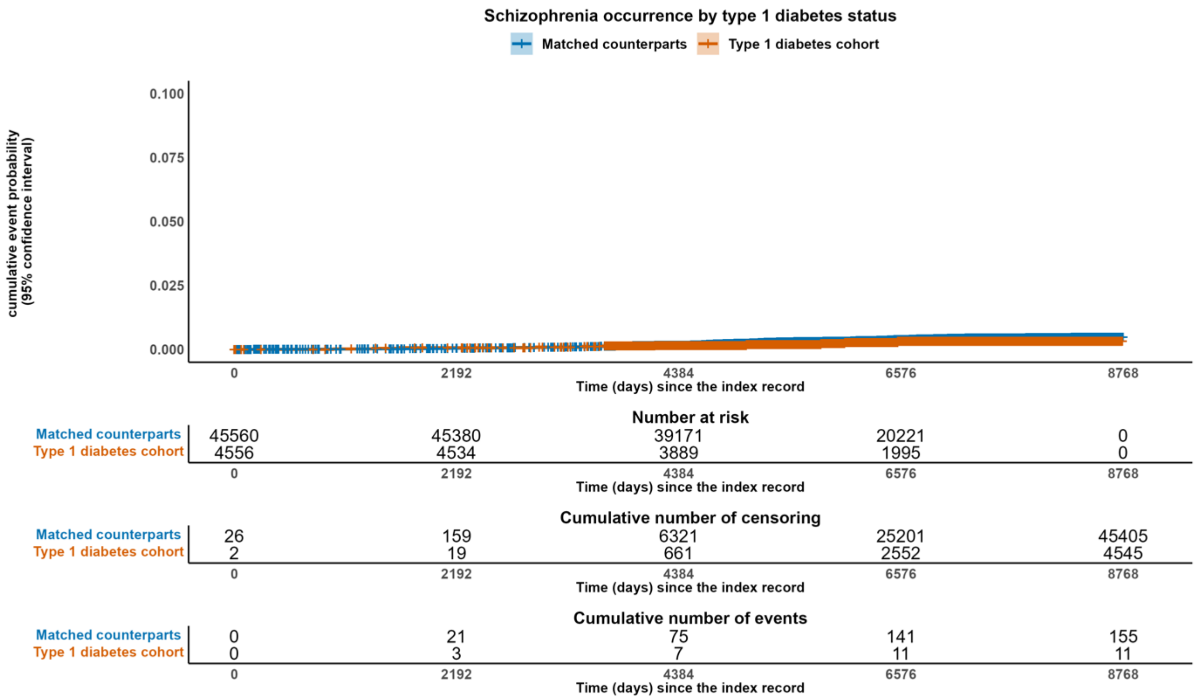

The curves in the plot depict the cumulative event probability per exposure status, accompanied by error bars expressed as 95% confidence intervals. The tables in the plot refer to the number of at risk individuals, cumulative number of censored individuals, and cumulative number of individuals who had the outcome per exposure status, respectively.

Supplementary Figure 12 Cumulative Event Plot on Developing Other Psychotic Disorders in Children with T1D

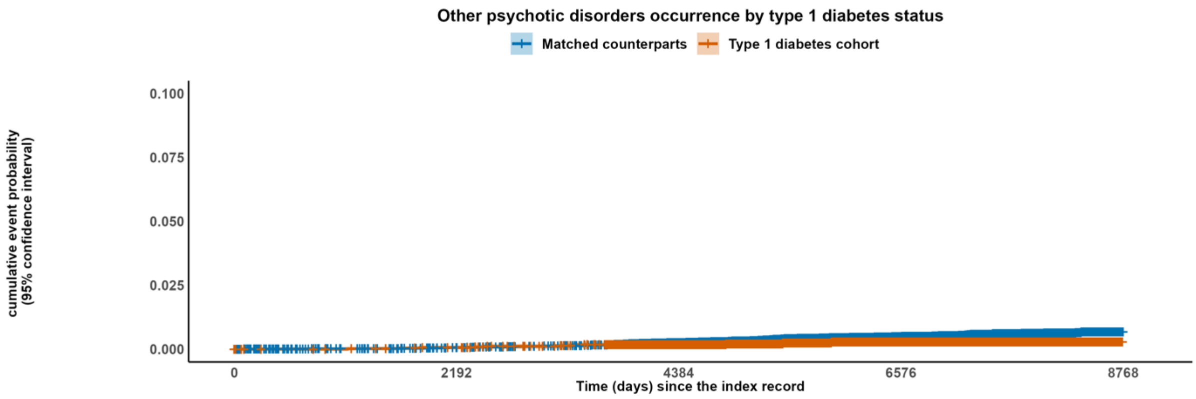

Number at risk

	0	2192	4384	6576	8768
Matched counterparts	45560	45371	39134	20210	0
Type 1 diabetes cohort	4556	4534	3887	1995	0

Time (days) since the index record

Cumulative number of censoring

	0	2192	4384	6576	8768
Matched counterparts	26	159	6319	25167	45351
Type 1 diabetes cohort	2	19	662	2552	4545

Time (days) since the index record

Cumulative number of events

	0	2192	4384	6576	8768
Matched counterparts	0	30	114	187	209
Type 1 diabetes cohort	0	3	8	11	11

Time (days) since the index record

The curves in the plot depict the cumulative event probability per exposure status, accompanied by error bars expressed as 95% confidence intervals. The tables in the plot refer to the number of at risk individuals, cumulative number of censored individuals, and cumulative number of individuals who had the outcome per exposure status, respectively.

Supplementary Figure 13 Cumulative Event Plot on Developing Mood Disorders in Children with T1D

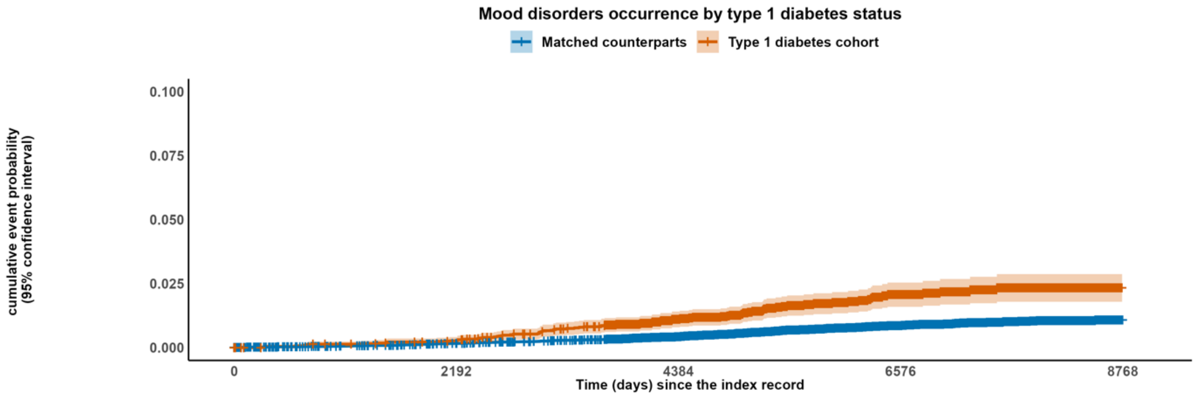

Number at risk

	0	2192	4384	6576	8768
Matched counterparts	45560	45327	39067	20114	0
Type 1 diabetes cohort	4556	4525	3856	1960	0

Time (days) since the index record

Cumulative number of censoring

	0	2192	4384	6576	8768
Matched counterparts	26	159	6311	25127	45208
Type 1 diabetes cohort	2	19	652	2521	4475

Time (days) since the index record

Cumulative number of events

	0	2192	4384	6576	8768
Matched counterparts	1	74	189	323	352
Type 1 diabetes cohort	0	12	49	77	81

Time (days) since the index record

The curves in the plot depict the cumulative event probability per exposure status, accompanied by error bars expressed as 95% confidence intervals. The tables in the plot refer to the number of at risk individuals, cumulative number of censored individuals, and cumulative number of individuals who had the outcome per exposure status, respectively.

Supplementary Figure 14 Cumulative Event Plot on Developing Bipolar Disorder in Children with T1D

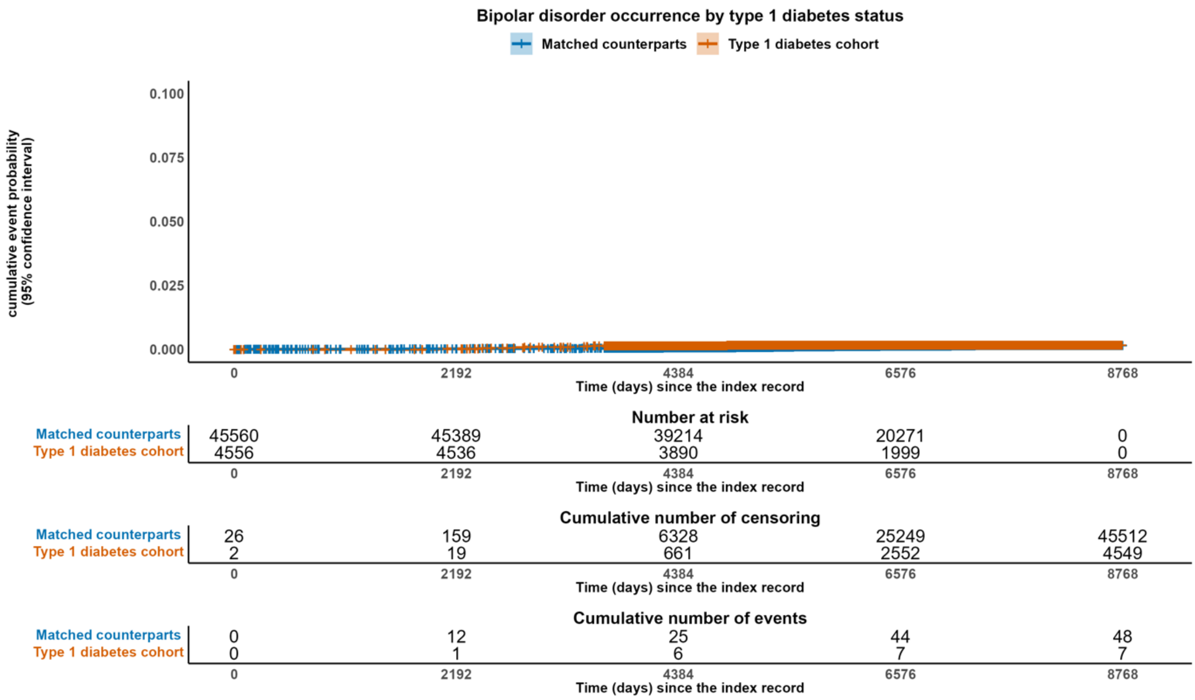

The curves in the plot depict the cumulative event probability per exposure status, accompanied by error bars expressed as 95% confidence intervals. The tables in the plot refer to the number of at risk individuals, cumulative number of censored individuals, and cumulative number of individuals who had the outcome per exposure status, respectively.

Supplementary Figure 15 Cumulative Event Plot on Developing Depression in Children with T1D

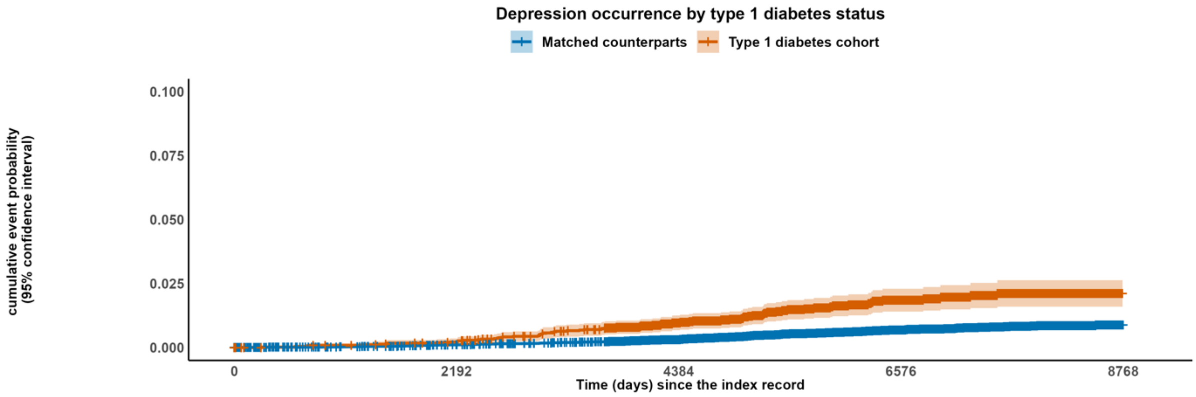

Number at risk

	0	2192	4384	6576	8768
Matched counterparts	45560	45352	39110	20150	0
Type 1 diabetes cohort	4556	4527	3862	1964	0

Time (days) since the index record

Cumulative number of censoring

	0	2192	4384	6576	8768
Matched counterparts	26	159	6318	25160	45280
Type 1 diabetes cohort	2	19	652	2525	4483

Time (days) since the index record

Cumulative number of events

	0	2192	4384	6576	8768
Matched counterparts	1	49	139	254	280
Type 1 diabetes cohort	0	10	43	69	73

Time (days) since the index record

The curves in the plot depict the cumulative event probability per exposure status, accompanied by error bars expressed as 95% confidence intervals. The tables in the plot refer to the number of at risk individuals, cumulative number of censored individuals, and cumulative number of individuals who had the outcome per exposure status, respectively.

Supplementary Figure 16 Cumulative Event Plot on Developing Other Mood Disorders in Children with T1D

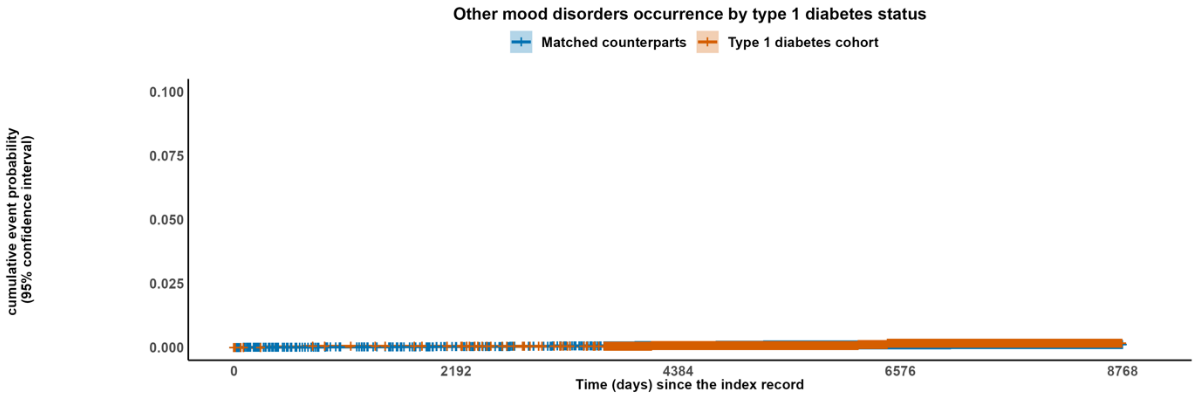

Number at risk

Time (days) since the index record	0	2192	4384	6576	8768
Matched counterparts	45560	45386	39203	20270	0
Type 1 diabetes cohort	4556	4535	3892	1995	0

Cumulative number of censoring

Time (days) since the index record	0	2192	4384	6576	8768
Matched counterparts	26	159	6330	25252	45516
Type 1 diabetes cohort	2	19	662	2558	4551

Cumulative number of events

Time (days) since the index record	0	2192	4384	6576	8768
Matched counterparts	0	15	34	42	44
Type 1 diabetes cohort	0	2	3	5	5

The curves in the plot depict the cumulative event probability per exposure status, accompanied by error bars expressed as 95% confidence intervals. The tables in the plot refer to the number of at risk individuals, cumulative number of censored individuals, and cumulative number of individuals who had the outcome per exposure status, respectively.

Supplementary Figure 17 Cumulative Event Plot on Developing Anxiety Disorders in Children with T1D

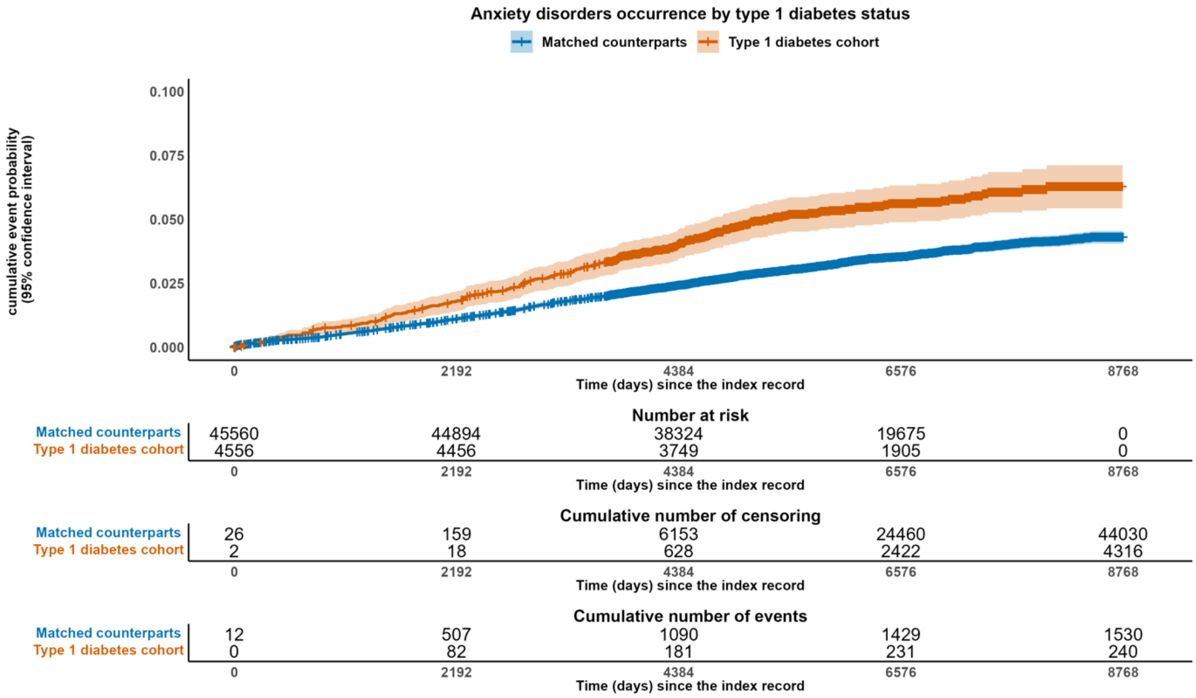

The curves in the plot depict the cumulative event probability per exposure status, accompanied by error bars expressed as 95% confidence intervals. The tables in the plot refer to the number of at risk individuals, cumulative number of censored individuals, and cumulative number of individuals who had the outcome per exposure status, respectively.

Supplementary Figure 18 Cumulative Event Plot on Developing Other Anxiety Disorders in Children with T1D

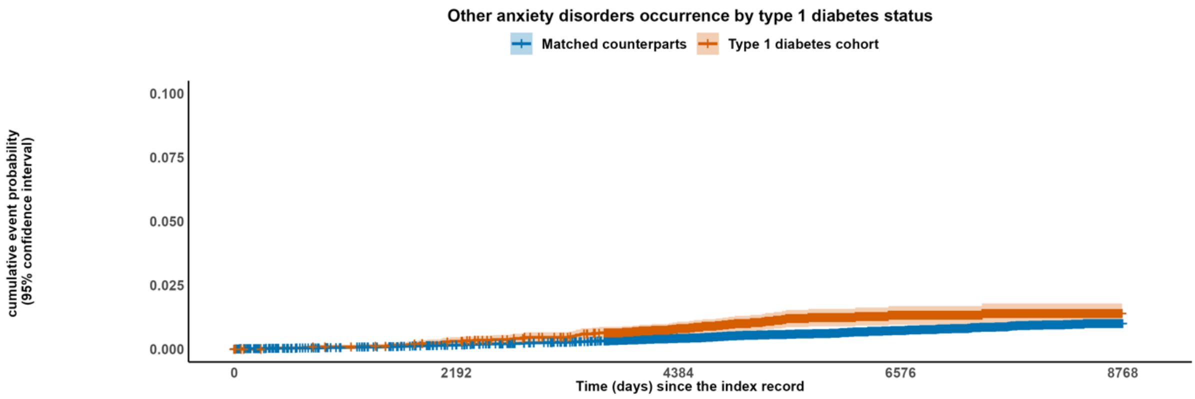

Number at risk

	0	2192	4384	6576	8768
Matched counterparts	45560	45329	39079	20167	0
Type 1 diabetes cohort	4556	4523	3869	1981	0

Time (days) since the index record

Cumulative number of censoring

	0	2192	4384	6576	8768
Matched counterparts	26	159	6300	25116	45245
Type 1 diabetes cohort	2	19	652	2525	4503

Time (days) since the index record

Cumulative number of events

	0	2192	4384	6576	8768
Matched counterparts	0	72	188	281	315
Type 1 diabetes cohort	0	14	36	52	53

Time (days) since the index record

The curves in the plot depict the cumulative event probability per exposure status, accompanied by error bars expressed as 95% confidence intervals. The tables in the plot refer to the number of at risk individuals, cumulative number of censored individuals, and cumulative number of individuals who had the outcome per exposure status, respectively.

Supplementary Figure 19 Cumulative Event Plot on Developing Panic Disorder in Children with T1D

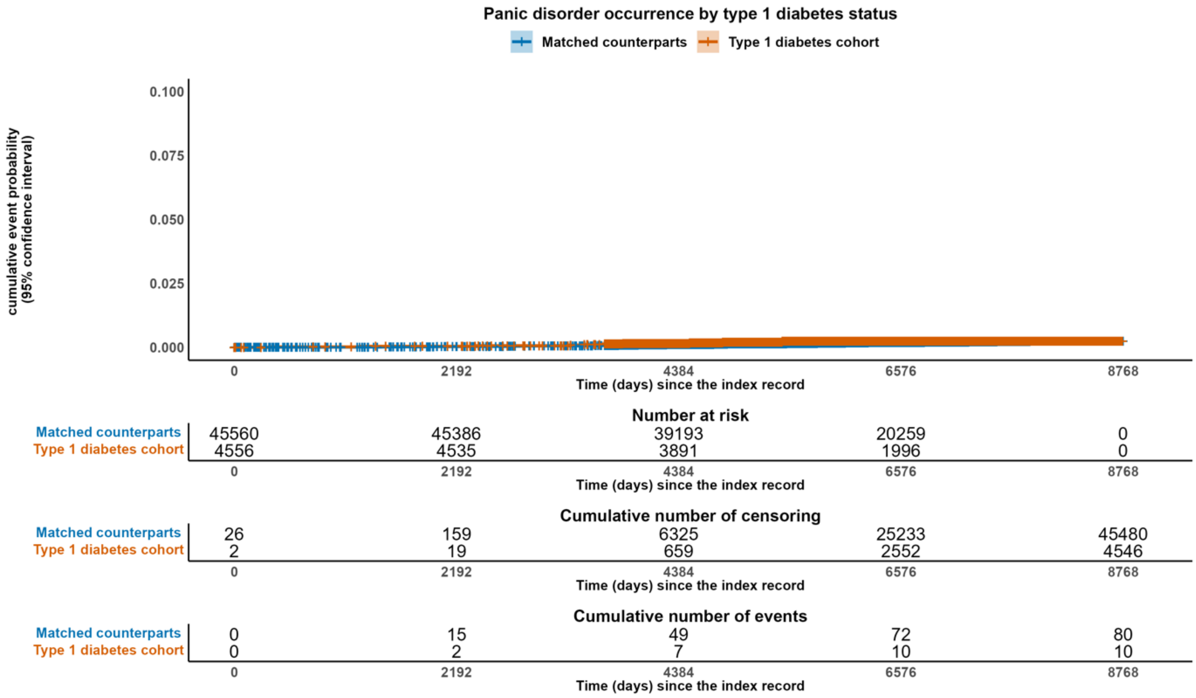

The curves in the plot depict the cumulative event probability per exposure status, accompanied by error bars expressed as 95% confidence intervals. The tables in the plot refer to the number of at risk individuals, cumulative number of censored individuals, and cumulative number of individuals who had the outcome per exposure status, respectively.

Supplementary Figure 20 Cumulative Event Plot on Developing Reaction to Severe Stress and Adjustment Disorders in Children with T1D

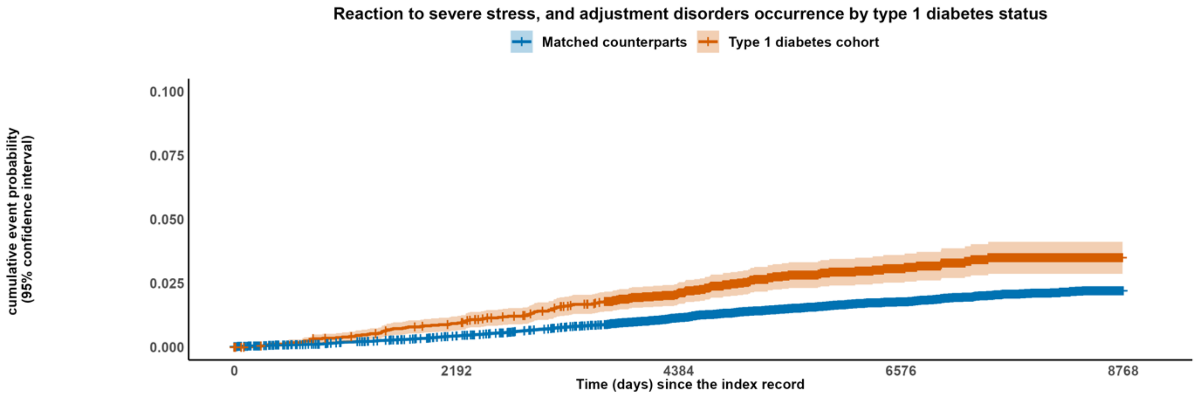

Number at risk

	0	2192	4384	6576	8768
Matched counterparts	45560	45201	38800	19981	0
Type 1 diabetes cohort	4556	4496	3818	1952	0

Time (days) since the index record

Cumulative number of censoring

	0	2192	4384	6576	8768
Matched counterparts	26	159	6253	24878	44795
Type 1 diabetes cohort	2	18	645	2481	4424

Time (days) since the index record

Cumulative number of events

	0	2192	4384	6576	8768
Matched counterparts	8	200	514	705	765
Type 1 diabetes cohort	0	42	95	125	132

Time (days) since the index record

The curves in the plot depict the cumulative event probability per exposure status, accompanied by error bars expressed as 95% confidence intervals. The tables in the plot refer to the number of at risk individuals, cumulative number of censored individuals, and cumulative number of individuals who had the outcome per exposure status, respectively.

Supplementary Figure 21 Cumulative Event Plot on Developing All Other Anxiety Disorders in Children with T1D

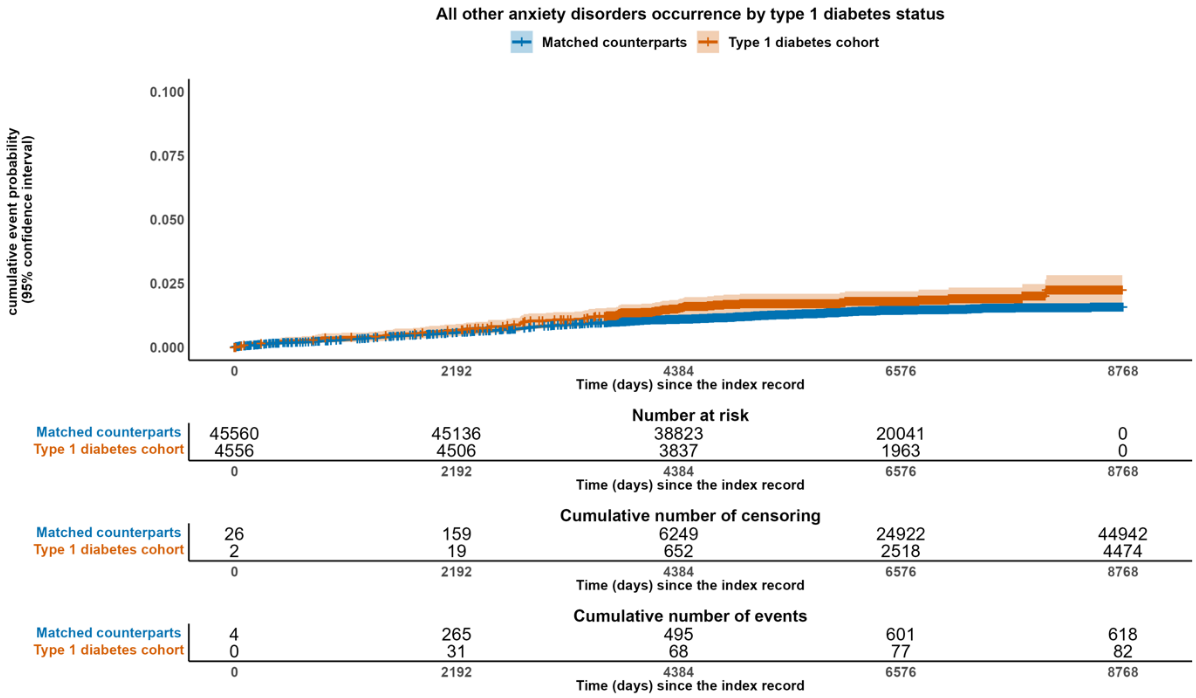

The curves in the plot depict the cumulative event probability per exposure status, accompanied by error bars expressed as 95% confidence intervals. The tables in the plot refer to the number of at risk individuals, cumulative number of censored individuals, and cumulative number of individuals who had the outcome per exposure status, respectively.

Supplementary Figure 22 Cumulative Event Plot on Developing Behavioural Syndromes in Children with T1D

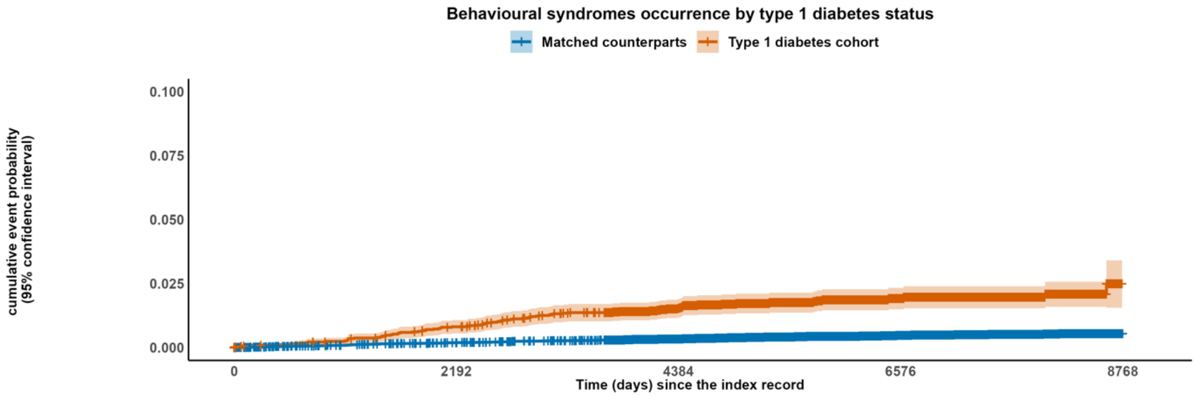

Number at risk

	0	2192	4384	6576	8768
Matched counterparts	45560	45312	39109	20219	0
Type 1 diabetes cohort	4556	4500	3832	1964	0

Time (days) since the index record

Cumulative number of censoring

	0	2192	4384	6576	8768
Matched counterparts	26	159	6306	25151	45356
Type 1 diabetes cohort	2	19	656	2513	4472

Time (days) since the index record

Cumulative number of events

	0	2192	4384	6576	8768
Matched counterparts	0	89	152	194	204
Type 1 diabetes cohort	0	37	69	81	84

Time (days) since the index record

The curves in the plot depict the cumulative event probability per exposure status, accompanied by error bars expressed as 95% confidence intervals. The tables in the plot refer to the number of at risk individuals, cumulative number of censored individuals, and cumulative number of individuals who had the outcome per exposure status, respectively.

Supplementary Figure 23 Cumulative Event Plot on Developing Eating Disorders in Children with T1D

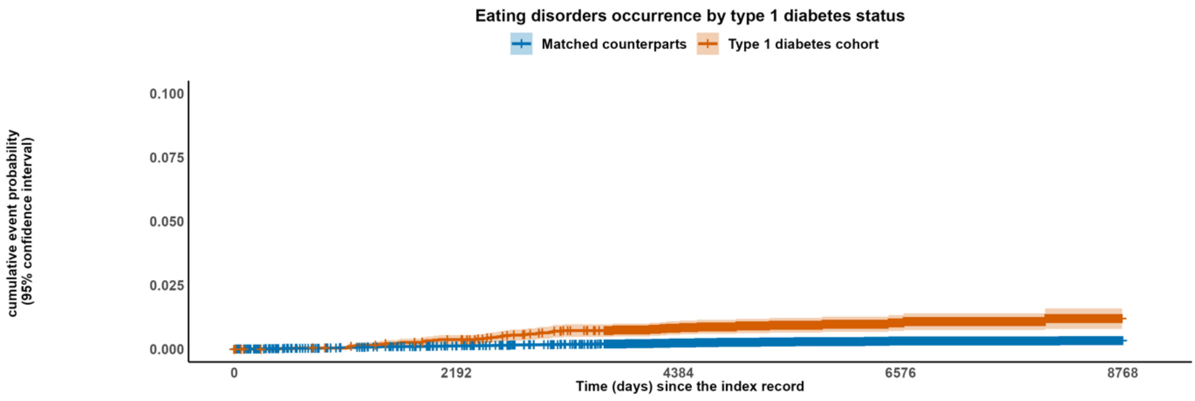

Number at risk

	0	2192	4384	6576	8768
Matched counterparts	45560	45342	39143	20236	0
Type 1 diabetes cohort	4556	4520	3864	1982	0

Time (days) since the index record

Cumulative number of censoring

	0	2192	4384	6576	8768
Matched counterparts	26	159	6317	25197	45428
Type 1 diabetes cohort	2	19	656	2533	4511

Time (days) since the index record

Cumulative number of events

	0	2192	4384	6576	8768
Matched counterparts	0	59	107	131	132
Type 1 diabetes cohort	0	17	37	43	45

Time (days) since the index record

The curves in the plot depict the cumulative event probability per exposure status, accompanied by error bars expressed as 95% confidence intervals. The tables in the plot refer to the number of at risk individuals, cumulative number of censored individuals, and cumulative number of individuals who had the outcome per exposure status, respectively.

Supplementary Figure 24 Cumulative Event Plot on Developing Anorexia Nervosa in Children with T1D

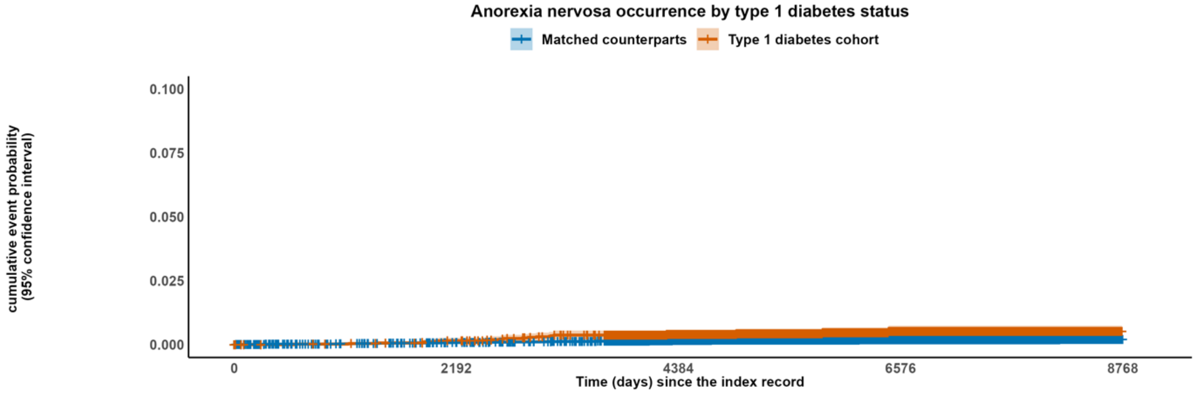

Number at risk

	0	2192	4384	6576	8768
Matched counterparts	45560	45363	39171	20254	0
Type 1 diabetes cohort	4556	4530	3878	1989	0

Time (days) since the index record

Cumulative number of censoring

	0	2192	4384	6576	8768
Matched counterparts	26	159	6325	25227	45476
Type 1 diabetes cohort	2	19	661	2548	4535

Time (days) since the index record

Cumulative number of events

	0	2192	4384	6576	8768
Matched counterparts	0	38	71	83	84
Type 1 diabetes cohort	0	7	18	21	21

Time (days) since the index record

The curves in the plot depict the cumulative event probability per exposure status, accompanied by error bars expressed as 95% confidence intervals. The tables in the plot refer to the number of at risk individuals, cumulative number of censored individuals, and cumulative number of individuals who had the outcome per exposure status, respectively.

Supplementary Figure 25 Cumulative Event Plot on Developing Bulimia Nervosa in Children with T1D

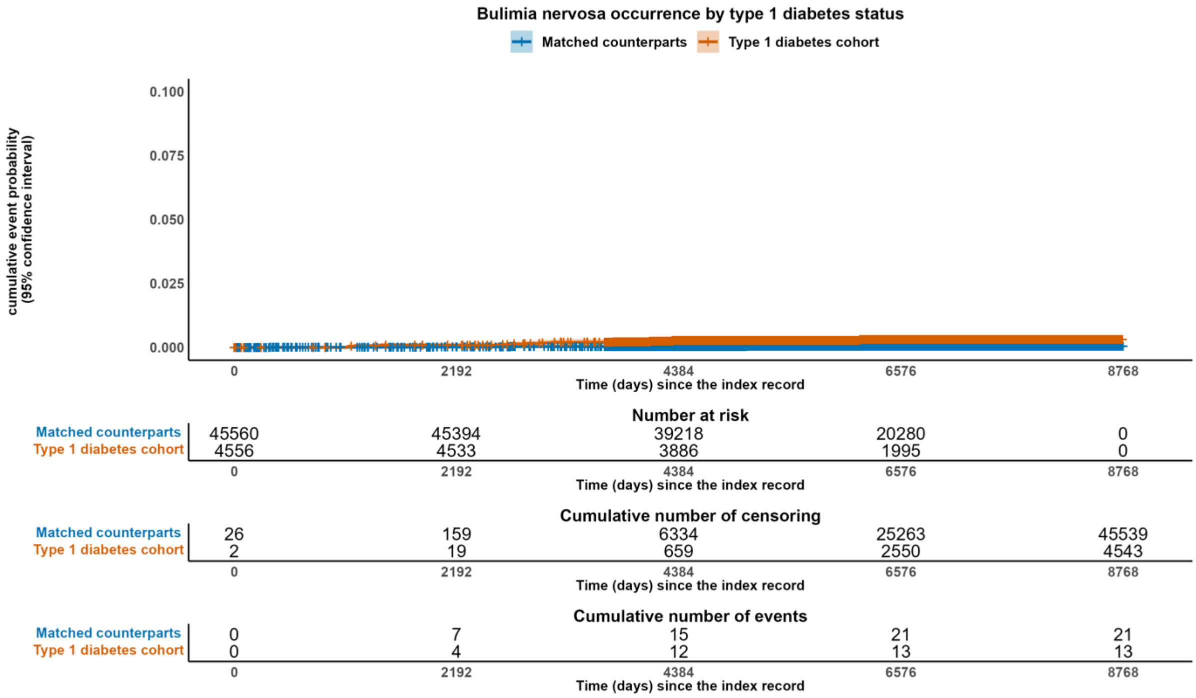

The curves in the plot depict the cumulative event probability per exposure status, accompanied by error bars expressed as 95% confidence intervals. The tables in the plot refer to the number of at risk individuals, cumulative number of censored individuals, and cumulative number of individuals who had the outcome per exposure status, respectively.

Supplementary Figure 26 Cumulative Event Plot on Developing Other Eating Disorders in Children with T1D

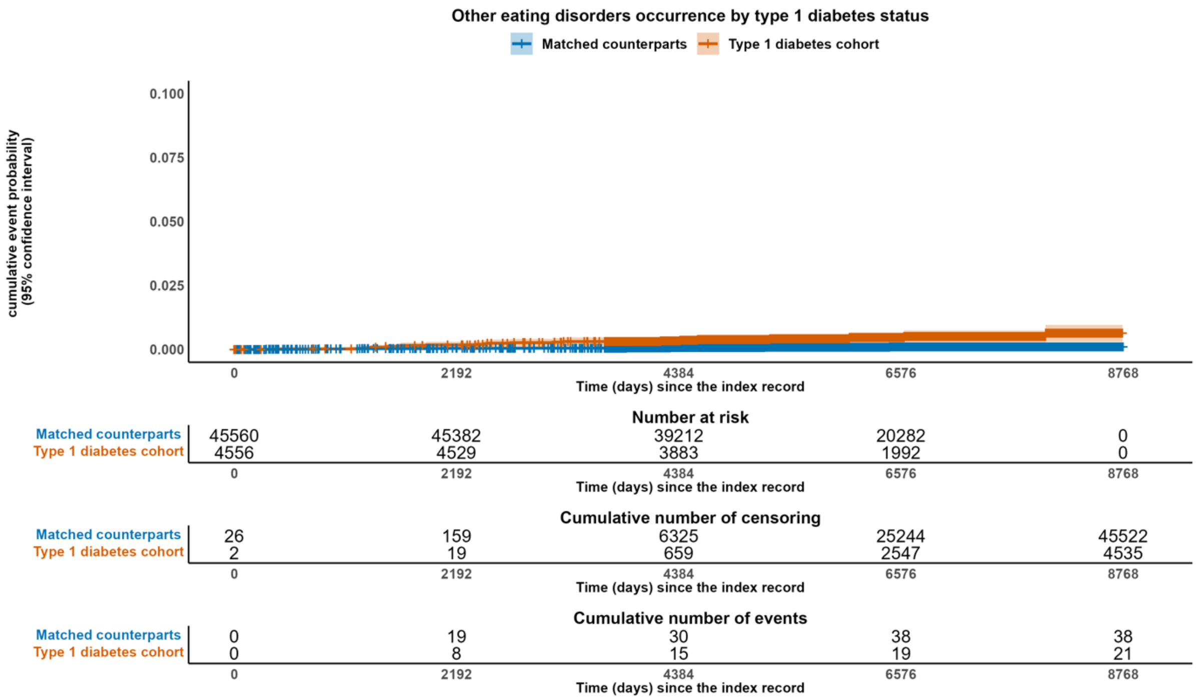

The curves in the plot depict the cumulative event probability per exposure status, accompanied by error bars expressed as 95% confidence intervals. The tables in the plot refer to the number of at risk individuals, cumulative number of censored individuals, and cumulative number of individuals who had the outcome per exposure status, respectively.

Supplementary Figure 27 Cumulative Event Plot on Developing Other Behavioural Syndromes in Children with T1D

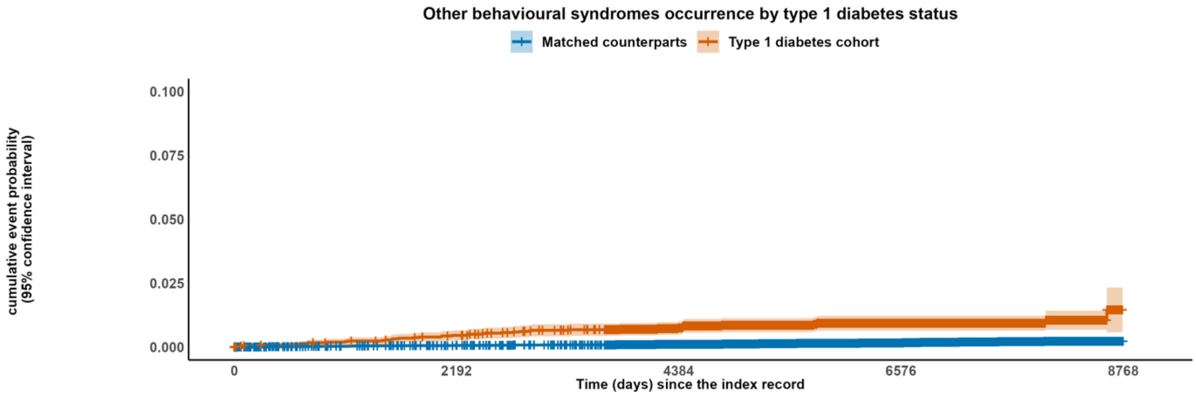

Number at risk

	0	2192	4384	6576	8768
Matched counterparts	45560	45370	39197	20273	0
Type 1 diabetes cohort	4556	4516	3861	1982	0

Time (days) since the index record

Cumulative number of censoring

	0	2192	4384	6576	8768
Matched counterparts	26	159	6322	25224	45484
Type 1 diabetes cohort	2	19	662	2536	4514

Time (days) since the index record

Cumulative number of events

	0	2192	4384	6576	8768
Matched counterparts	0	31	48	67	76
Type 1 diabetes cohort	0	21	34	40	42

Time (days) since the index record

The curves in the plot depict the cumulative event probability per exposure status, accompanied by error bars expressed as 95% confidence intervals. The tables in the plot refer to the number of at risk individuals, cumulative number of censored individuals, and cumulative number of individuals who had the outcome per exposure status, respectively.

Supplementary Figure 28 Cumulative Event Plot on Developing Personality Disorders in Children with T1D

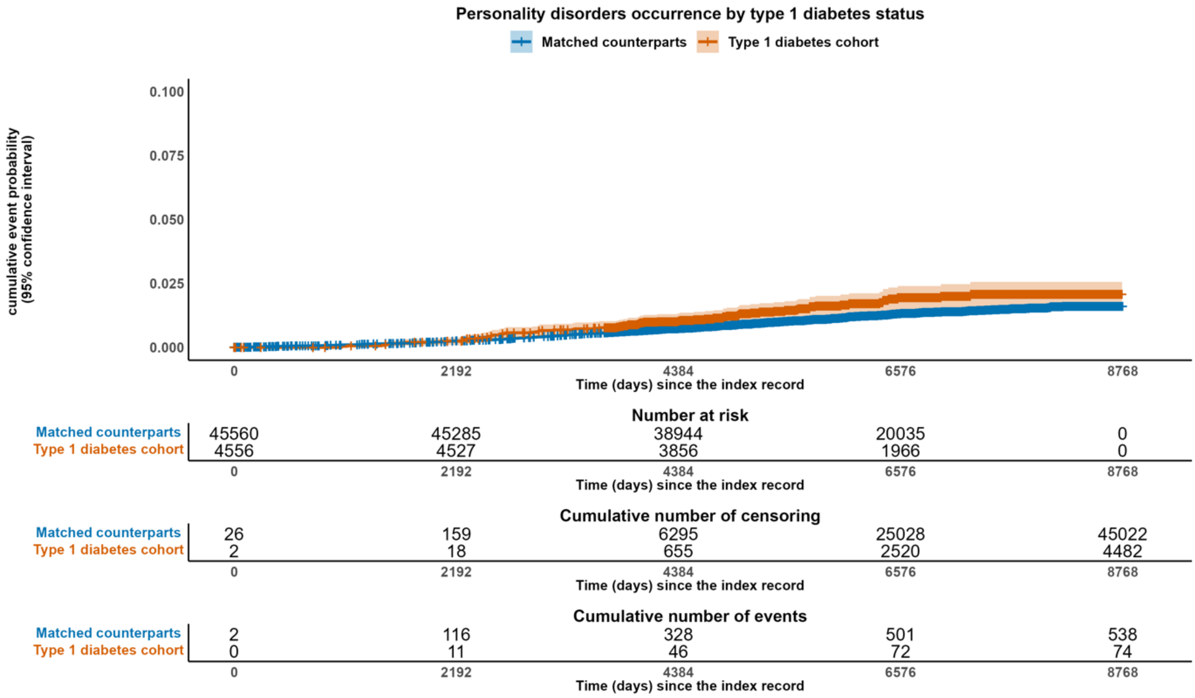

The curves in the plot depict the cumulative event probability per exposure status, accompanied by error bars expressed as 95% confidence intervals. The tables in the plot refer to the number of at risk individuals, cumulative number of censored individuals, and cumulative number of individuals who had the outcome per exposure status, respectively.

Supplementary Figure 29 Cumulative Event Plot on Developing Specific Personality Disorders in Children with T1D

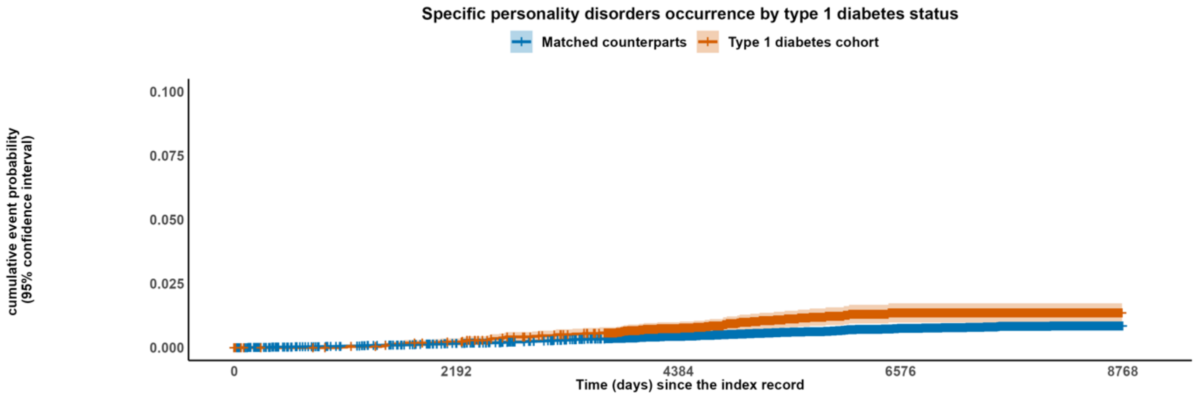

Number at risk

	0	2192	4384	6576	8768
Matched counterparts	45560	45326	39059	20155	0
Type 1 diabetes cohort	4556	4528	3868	1978	0

Time (days) since the index record

Cumulative number of censoring

	0	2192	4384	6576	8768
Matched counterparts	26	159	6311	25120	45258
Type 1 diabetes cohort	2	18	656	2528	4504

Time (days) since the index record

Cumulative number of events

	0	2192	4384	6576	8768
Matched counterparts	0	75	197	289	302
Type 1 diabetes cohort	0	10	33	52	52

Time (days) since the index record

The curves in the plot depict the cumulative event probability per exposure status, accompanied by error bars expressed as 95% confidence intervals. The tables in the plot refer to the number of at risk individuals, cumulative number of censored individuals, and cumulative number of individuals who had the outcome per exposure status, respectively.

Supplementary Figure 30 Cumulative Event Plot on Developing Other Personality Disorders in Children with T1D

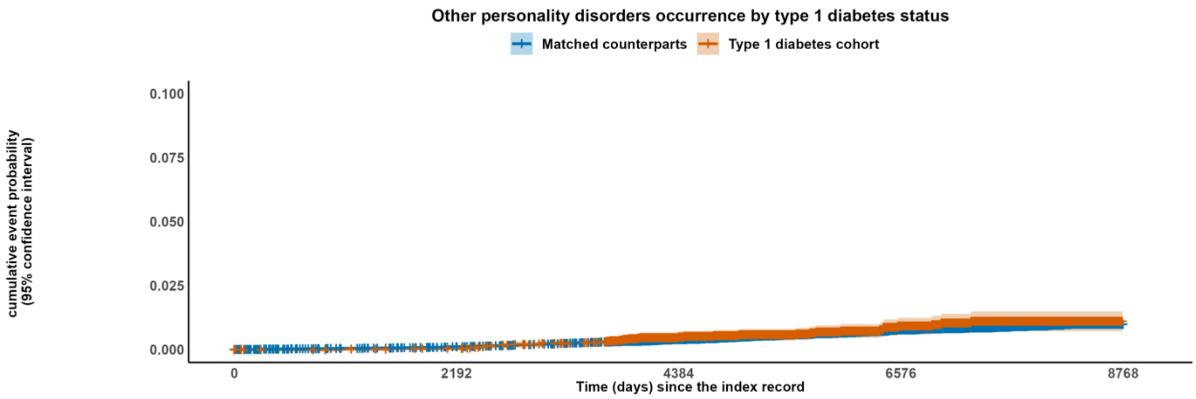

Number at risk

Time (days) since the index record	0	2192	4384	6576	8768
Matched counterparts	45560	45349	39083	20136	0
Type 1 diabetes cohort	4556	4535	3876	1981	0

Cumulative number of censoring

Time (days) since the index record	0	2192	4384	6576	8768
Matched counterparts	26	159	6312	25146	45250
Type 1 diabetes cohort	2	19	659	2544	4520

Cumulative number of events

Time (days) since the index record	0	2192	4384	6576	8768
Matched counterparts	2	52	172	282	310
Type 1 diabetes cohort	0	2	22	33	36

The curves in the plot depict the cumulative event probability per exposure status, accompanied by error bars expressed as 95% confidence intervals. The tables in the plot refer to the number of at risk individuals, cumulative number of censored individuals, and cumulative number of individuals who had the outcome per exposure status, respectively.

Supplementary Figure 31 Risk of Occurrence of Psychiatric Disorders in Individuals with Childhood-onset T1D Sensitivity Analysis, T1D Diagnosed up-to 9 Years of Age

	Matched counterparts n events/n total	Type 1 diabetes cohort n events/n total	Matched counterparts incidence rate per 100,000 person−years	Type 1 diabetes cohort incidence rate per 100,000 person−years	Incidence rate difference per 100,000 person−years	Adjusted hazard ratio (95% confidence interval)
Substance use disorders	812/23,680	111/2,368	204.57	282.06	77.49	1.38 (1.13; 1.68)
Alcohol use disorders	497/23,680	77/2,368	124.64	194.63	70.00	1.57 (1.23; 1.99)
Drug use disorders	390/23,680	42/2,368	97.61	105.59	7.98	1.08 (0.78; 1.48)
Cannabis use disorders	103/23,680	9/2,368	25.67	22.52	−3.15	0.88 (0.45; 1.74)
Other non−alcohol substance use disorders	326/23,680	35/2,368	81.52	87.89	6.37	1.08 (0.76; 1.53)
Mood disorders	113/23,680	30/2,368	28.18	75.31	47.13	2.63 (1.76; 3.94)
Depression	90/23,680	24/2,368	22.43	60.18	37.74	2.62 (1.67; 4.11)
Anxiety disorders	659/23,680	108/2,368	166.05	275.25	109.21	1.65 (1.35; 2.02)
Other anxiety disorders	136/23,680	24/2,368	33.92	60.16	26.23	1.77 (1.15; 2.74)
Panic disorder	36/23,680	5/2,368	8.97	12.50	3.54	1.38 (0.54; 3.51)
Reaction to severe stress, and adjustment disorders	294/23,680	54/2,368	73.52	136.17	62.65	1.84 (1.38; 2.47)
All other anxiety disorders	295/23,680	44/2,368	73.90	110.92	37.02	1.50 (1.09; 2.05)
Behavioural syndromes	107/23,680	31/2,368	26.69	78.01	51.32	2.87 (1.92; 4.28)
Eating disorders	69/23,680	12/2,368	17.20	30.04	12.85	1.73 (0.93; 3.18)
Other eating disorders	30/23,680	7/2,368	7.47	17.51	10.04	2.33 (1.02; 5.30)
Other behavioural syndromes	40/23,680	21/2,368	9.97	52.76	42.79	5.20 (3.07; 8.82)
Personality disorders	201/23,680	35/2,368	50.18	87.95	37.77	1.72 (1.20; 2.46)
Specific personality disorders	119/23,680	25/2,368	29.67	62.70	33.03	2.07 (1.35; 3.19)
Other personality disorders	103/23,680	15/2,368	25.67	37.57	11.90	1.45 (0.84; 2.49)

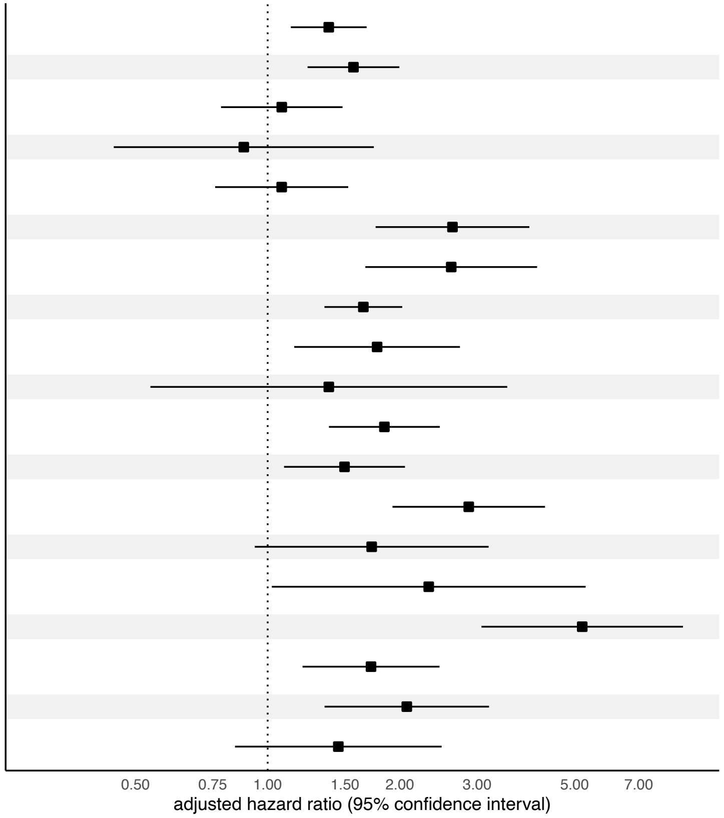

Incidence rates were calculated per 100,000 person-years. Incidence rate differences denote the difference between the incidence rate in individuals with T1D and their matched counterparts, and are expressed per 100,000 person-years. The associations between T1D and psychiatric disorders are expressed as adjusted hazard ratios, accompanied by error bars expressed as 95% confidence intervals.

Supplementary Figure 32 Risk of Occurrence of Psychiatric Disorders in Individuals with Childhood-onset T1D Sensitivity Analysis, Incident Cases of T1D

	Matched counterparts n events/n total	Type 1 diabetes cohort n events/n total	Matched counterparts incidence rate per 100,000 person–years	Type 1 diabetes cohort incidence rate per 100,000 person–years	Incidence rate difference per 100,000 person–years	Adjusted hazard ratio (95% confidence interval)
Substance use disorders	472/17,290	79/1,729	197.15	333.02	135.87	1.68 (1.32; 2.13)
Alcohol use disorders	297/17,290	56/1,729	123.66	235.03	111.37	1.87 (1.40; 2.49)
Drug use disorders	216/17,290	27/1,729	89.76	112.71	22.94	1.26 (0.84; 1.87)
Cannabis use disorders	72/17,290	7/1,729	29.85	29.12	–0.73	0.96 (0.44; 2.08)
Other non–alcohol substance use disorders	174/17,290	22/1,729	72.25	91.73	19.48	1.26 (0.81; 1.97)
Mood disorders	72/17,290	16/1,729	29.85	66.65	36.80	2.21 (1.28; 3.79)
Depression	52/17,290	14/1,729	21.55	58.30	36.76	2.67 (1.48; 4.81)
Anxiety disorders	518/17,290	85/1,729	217.22	361.26	144.04	1.67 (1.33; 2.10)
Other anxiety disorders	115/17,290	22/1,729	47.73	91.87	44.15	1.89 (1.20; 2.99)
Reaction to severe stress, and adjustment disorders	207/17,290	45/1,729	86.07	189.09	103.02	2.19 (1.59; 3.03)
All other anxiety disorders	258/17,290	30/1,729	107.57	125.72	18.14	1.18 (0.81; 1.72)
Behavioural syndromes	79/17,290	19/1,729	32.78	79.39	46.62	2.38 (1.44; 3.93)
Eating disorders	46/17,290	10/1,729	19.06	41.65	22.58	2.17 (1.09; 4.30)
Bulimia nervosa	6/17,290	5/1,729	2.48	20.79	18.30	8.25 (2.52; 27.05)
Other eating disorders	15/17,290	7/1,729	6.21	29.14	22.93	4.64 (1.89; 11.39)
Other behavioural syndromes	35/17,290	11/1,729	14.51	45.88	31.37	3.10 (1.57; 6.10)
Personality disorders	115/17,290	24/1,729	47.71	100.18	52.47	2.07 (1.33; 3.21)
Specific personality disorders	61/17,290	17/1,729	25.28	70.86	45.58	2.76 (1.61; 4.73)
Other personality disorders	63/17,290	8/1,729	26.11	33.28	7.17	1.26 (0.60; 2.63)

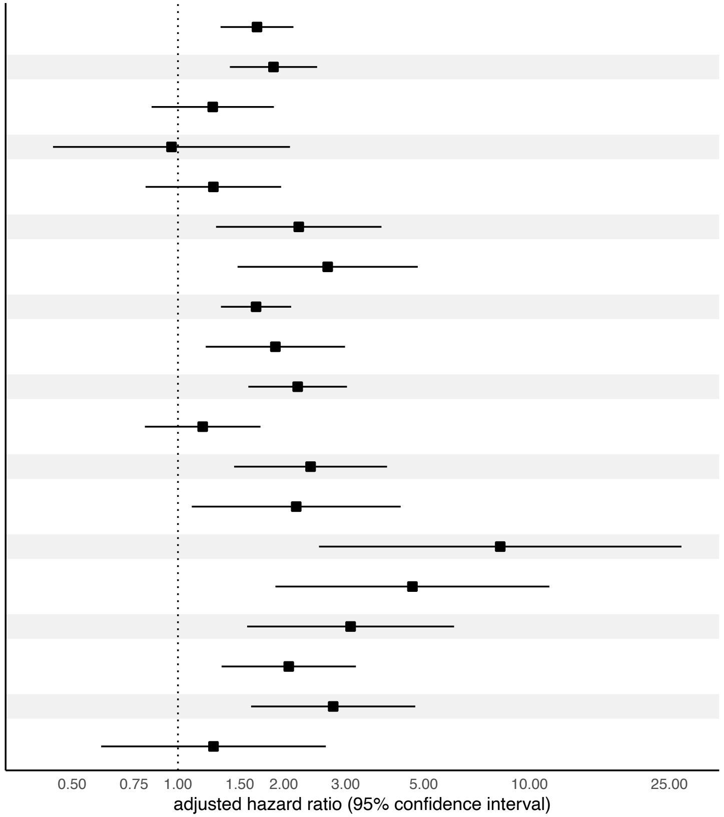

Incidence rates were calculated per 100,000 person-years. Incidence rate differences denote the difference between the incidence rate in individuals with T1D and their matched counterparts, and are expressed per 100,000 person-years. The associations between T1D and psychiatric disorders are expressed as adjusted hazard ratios, accompanied by error bars expressed as 95% confidence intervals.

Supplementary Figure 33 Risk of Occurrence of Psychiatric Disorders in Individuals with Childhood-onset T1D Sensitivity Analysis, Adjustment for the Number of Previous Hospitalizations

	Matched counterparts n events/n total	Type 1 diabetes cohort n events/n total	Matched counterparts incidence rate per 100,000 person−years	Type 1 diabetes cohort incidence rate per 100,000 person−years	Incidence rate difference per 100,000 person−years	Adjusted hazard ratio (95% confidence interval)
Substance use disorders	973/19,341	256/3,878	326.69	431.54	104.85	1.32 (1.15; 1.52)
Alcohol use disorders	475/19,341	162/3,878	157.66	270.29	112.63	1.70 (1.42; 2.04)
Drug use disorders	587/19,341	119/3,878	195.27	197.32	2.05	1.01 (0.83; 1.23)
Opioid use disorders	57/19,341	12/3,878	18.74	19.68	0.93	1.04 (0.56; 1.93)
Cannabis use disorders	127/19,341	27/3,878	41.80	44.36	2.56	1.09 (0.72; 1.65)
Other non−alcohol substance use disorders	527/19,341	105/3,878	175.04	173.74	−1.30	0.98 (0.80; 1.22)
Psychotic disorders	144/19,341	15/3,878	47.40	24.61	−22.79	0.52 (0.30; 0.88)
Schizophrenia	72/19,341	10/3,878	23.67	16.39	−7.27	0.67 (0.34; 1.30)
Other psychotic disorders	113/19,341	10/3,878	37.17	16.40	−20.77	0.43 (0.23; 0.83)
Mood disorders	174/19,341	73/3,878	57.34	120.56	63.22	2.10 (1.59; 2.77)
Bipolar disorder	28/19,341	7/3,878	9.19	11.47	2.28	1.25 (0.55; 2.86)
Depression	137/19,341	65/3,878	45.10	107.21	62.11	2.39 (1.77; 3.22)
Other mood disorders	17/19,341	5/3,878	5.58	8.20	2.61	1.35 (0.49; 3.67)
Anxiety disorders	714/19,341	218/3,878	239.02	367.80	128.78	1.50 (1.29; 1.75)
Other anxiety disorders	170/19,341	48/3,878	56.03	79.01	22.98	1.39 (1.01; 1.91)
Panic disorder	32/19,341	9/3,878	10.51	14.75	4.24	1.40 (0.67; 2.93)
Reaction to severe stress, and adjustment disorders	315/19,341	124/3,878	104.16	206.37	102.21	1.97 (1.60; 2.43)
All other anxiety disorders	324/19,341	70/3,878	107.43	115.87	8.44	1.05 (0.81; 1.36)
Behavioural syndromes	119/19,341	76/3,878	39.18	126.01	86.83	3.20 (2.39; 4.27)
Eating disorders	73/19,341	40/3,878	24.00	65.88	41.88	2.70 (1.83; 3.98)
Anorexia nervosa	45/19,341	18/3,878	14.79	29.57	14.78	1.97 (1.14; 3.40)
Bulimia nervosa	12/19,341	11/3,878	3.94	18.04	14.10	4.54 (2.00; 10.29)
Other eating disorders	20/19,341	20/3,878	6.57	32.84	26.27	4.77 (2.56; 8.87)
Other behavioural syndromes	48/19,341	39/3,878	15.78	64.32	48.54	3.99 (2.61; 6.09)
Personality disorders	244/19,341	65/3,878	80.53	107.23	26.70	1.28 (0.97; 1.69)
Specific personality disorders	160/19,341	48/3,878	52.71	79.04	26.32	1.44 (1.04; 1.99)
Other personality disorders	116/19,341	31/3,878	38.17	50.94	12.77	1.29 (0.86; 1.92)

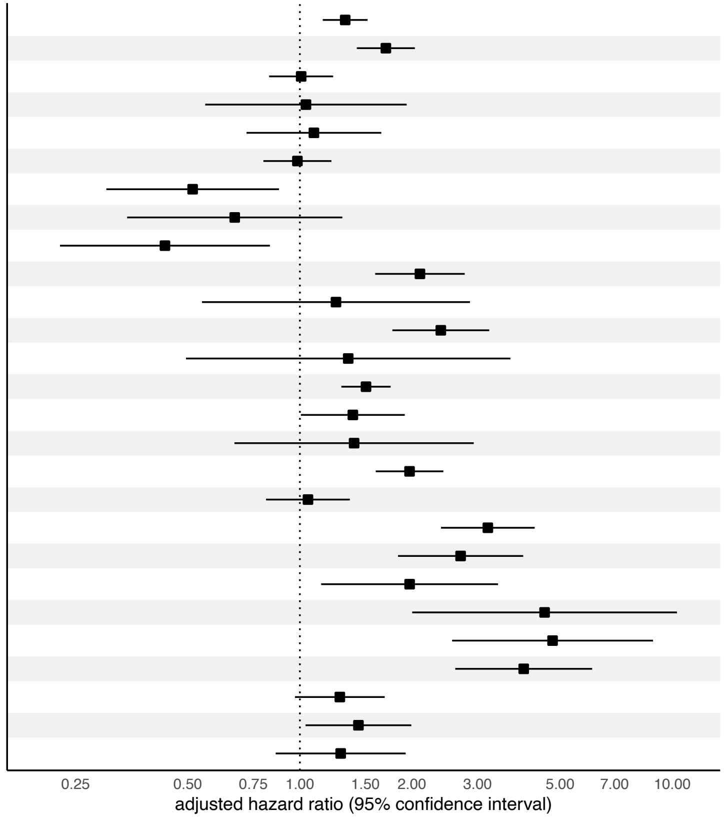

Incidence rates were calculated per 100,000 person-years. Incidence rate differences denote the difference between the incidence rate in individuals with T1D and their matched counterparts, and are expressed per 100,000 person-years. The associations between T1D and psychiatric disorders are expressed as adjusted hazard ratios, accompanied by error bars expressed as 95% confidence intervals.

Supplementary Figure 34 Risk of Occurrence of Psychiatric Disorders in Individuals with Childhood-onset T1D Sensitivity Analysis, Comparison with Individuals with Asthma

	Matched counterparts n events/n total	Type 1 diabetes cohort n events/n total	Matched counterparts incidence rate per 100,000 person−years	Type 1 diabetes cohort incidence rate per 100,000 person−years	Incidence rate difference per 100,000 person−years	Adjusted hazard ratio (95% confidence interval)
Substance use disorders	765/13,365	286/4,458	344.65	388.44	43.79	1.13 (0.99; 1.30)
Alcohol use disorders	412/13,365	177/4,458	183.49	238.03	54.54	1.31 (1.10; 1.57)
Drug use disorders	459/13,365	138/4,458	204.57	184.64	−19.93	0.91 (0.75; 1.10)
Opioid use disorders	30/13,365	15/4,458	13.20	19.85	6.66	1.56 (0.82; 2.97)
Cannabis use disorders	84/13,365	30/4,458	37.00	39.77	2.78	1.09 (0.71; 1.65)
Other non−alcohol substance use disorders	408/13,365	121/4,458	181.52	161.58	−19.94	0.90 (0.73; 1.10)
Psychotic disorders	103/13,365	16/4,458	45.40	21.19	−24.22	0.47 (0.28; 0.80)
Schizophrenia	59/13,365	11/4,458	25.97	14.56	−11.42	0.50 (0.26; 0.98)
Other psychotic disorders	71/13,365	11/4,458	31.27	14.56	−16.71	0.48 (0.25; 0.91)
Mood disorders	134/13,365	79/4,458	59.14	105.22	46.08	1.79 (1.35; 2.37)
Bipolar disorder	27/13,365	7/4,458	11.87	9.26	−2.61	0.82 (0.35; 1.89)
Depression	102/13,365	71/4,458	44.96	94.47	49.51	2.12 (1.56; 2.88)
Other mood disorders	19/13,365	5/4,458	8.36	6.61	−1.74	0.80 (0.30; 2.16)
Anxiety disorders	584/13,365	234/4,458	262.37	317.63	55.26	1.19 (1.03; 1.39)
Other anxiety disorders	131/13,365	52/4,458	57.81	69.05	11.25	1.18 (0.86; 1.63)
Panic disorder	27/13,365	10/4,458	11.87	13.23	1.36	1.08 (0.52; 2.24)
Reaction to severe stress, and adjustment disorders	276/13,365	127/4,458	122.37	170.15	47.78	1.37 (1.11; 1.70)
All other anxiety disorders	261/13,365	79/4,458	115.96	105.51	−10.46	0.90 (0.70; 1.16)
Behavioural syndromes	105/13,365	80/4,458	46.35	106.87	60.52	2.33 (1.73; 3.14)
Eating disorders	61/13,365	41/4,458	26.88	54.46	27.58	2.07 (1.38; 3.10)
Anorexia nervosa	39/13,365	20/4,458	17.17	26.51	9.34	1.66 (0.95; 2.89)
Bulimia nervosa	15/13,365	12/4,458	6.59	15.88	9.29	2.37 (1.11; 5.06)
Other eating disorders	21/13,365	18/4,458	9.24	23.84	14.61	2.68 (1.40; 5.11)
Other behavioural syndromes	47/13,365	41/4,458	20.69	54.52	33.83	2.59 (1.69; 3.97)
Personality disorders	205/13,365	72/4,458	90.67	95.84	5.17	1.05 (0.80; 1.37)
Specific personality disorders	124/13,365	51/4,458	54.71	67.75	13.04	1.24 (0.89; 1.72)
Other personality disorders	112/13,365	35/4,458	49.38	46.42	−2.96	0.94 (0.64; 1.37)

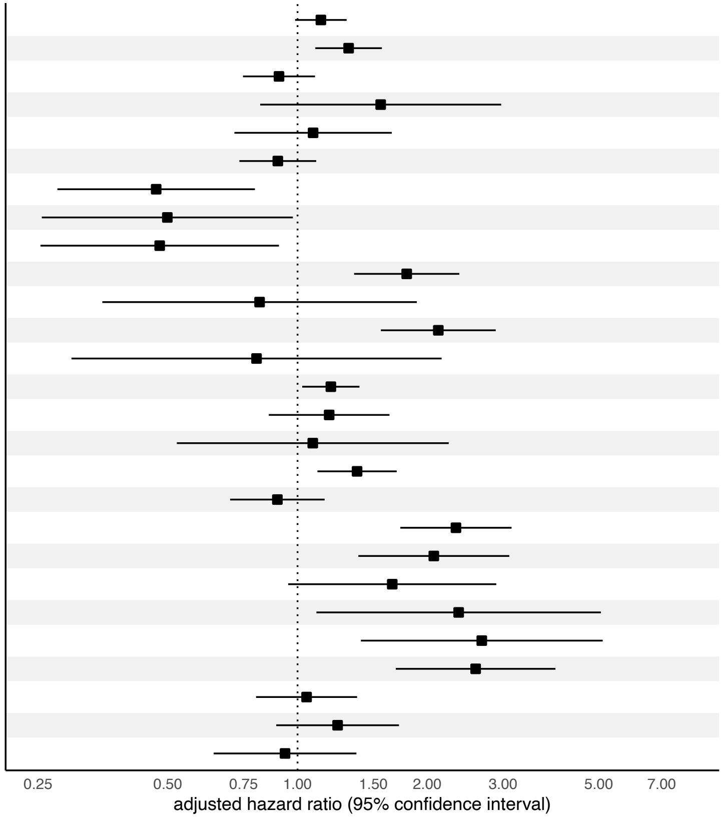

Incidence rates were calculated per 100,000 person-years. Incidence rate differences denote the difference between the incidence rate in individuals with T1D and their matched counterparts, and are expressed per 100,000 person-years. The associations between T1D and psychiatric disorders are expressed as adjusted hazard ratios, accompanied by error bars expressed as 95% confidence intervals.

Supplementary Figure 35 Scatterplots for MR Analyses with Evidence for an Association of T1D with Subsequent Mental Disorders, *Trans* Instrument with Schizophrenia

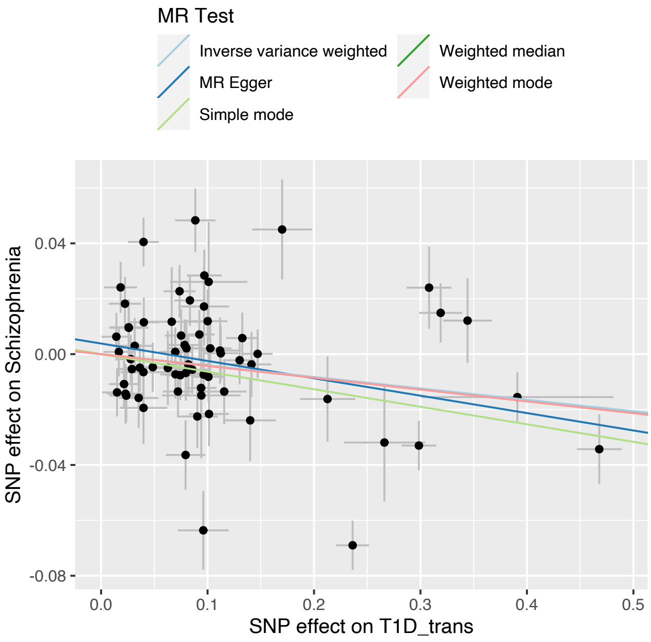

The scatterplot depicts single-nucleotide polymorphism (SNP)–exposure and SNP–outcome associations for each SNP with fitted lines estimated using five Mendelian randomization methods [24]. The point estimates are accompanied by error bars, denoting the standard error of the estimated association between the SNP and the exposure and the outcome, respectively [24].

Supplementary Figure 36 Scatterplots for MR Analyses with Evidence for an Association of T1D with Subsequent Mental Disorders, *GLIS3 Cis* Instrument with Schizophrenia

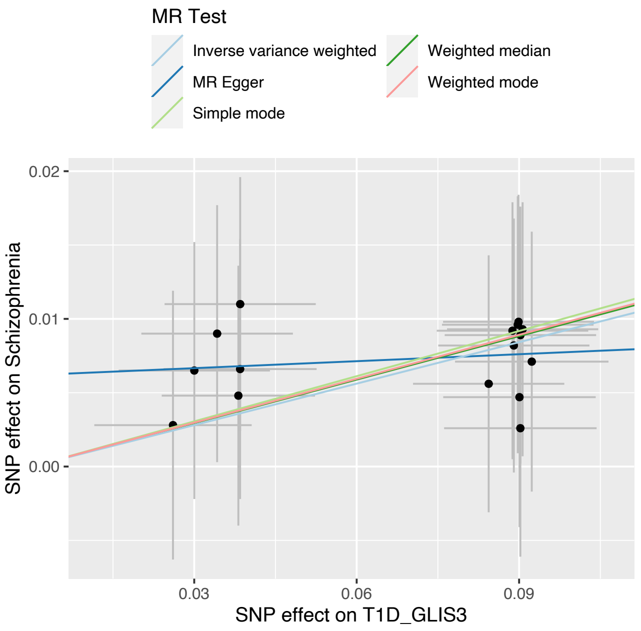

The scatterplot depicts single-nucleotide polymorphism (SNP)–exposure and SNP–outcome associations for each SNP with fitted lines estimated using five Mendelian randomization methods [24]. The point estimates are accompanied by error bars, denoting the standard error of the estimated association between the SNP and the exposure and the outcome, respectively [24].

Supplementary Figure 37 Scatterplots for MR Analyses with Evidence for an Association of T1D with Subsequent Mental Disorders, *CTSH Cis* Instrument with Schizophrenia

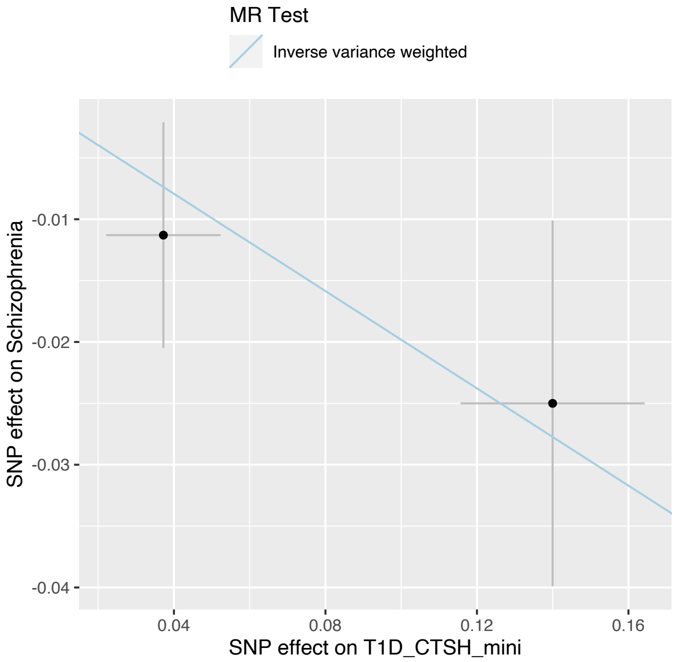

The scatterplot depicts single-nucleotide polymorphism (SNP)–exposure and SNP–outcome associations for each SNP with fitted lines estimated using five Mendelian randomization methods [24]. The point estimates are accompanied by error bars, denoting the standard error of the estimated association between the SNP and the exposure and the outcome, respectively [24].

Supplementary Figure 38 Scatterplots for MR Analyses with Evidence for an Association of T1D with Subsequent Mental Disorders, *GLIS3 Cis* Instrument with Anxiety Disorders

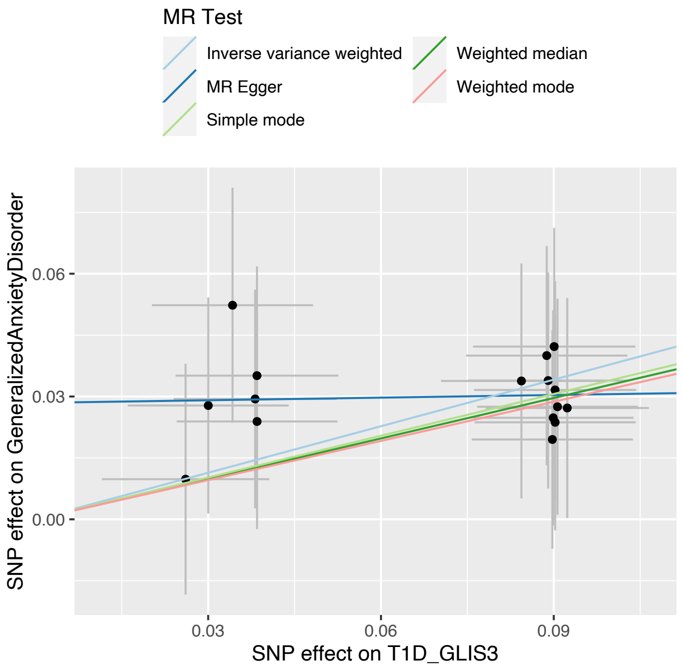

The scatterplot depicts single-nucleotide polymorphism (SNP)–exposure and SNP–outcome associations for each SNP with fitted lines estimated using five Mendelian randomization methods [24]. The point estimates are accompanied by error bars, denoting the standard error of the estimated association between the SNP and the exposure and the outcome, respectively [24].

Supplementary Figure 39 Scatterplots for MR Analyses with Evidence for an Association of T1D with Subsequent Mental Disorders, *GLIS3* Cis instrument with Major Depressive Disorder

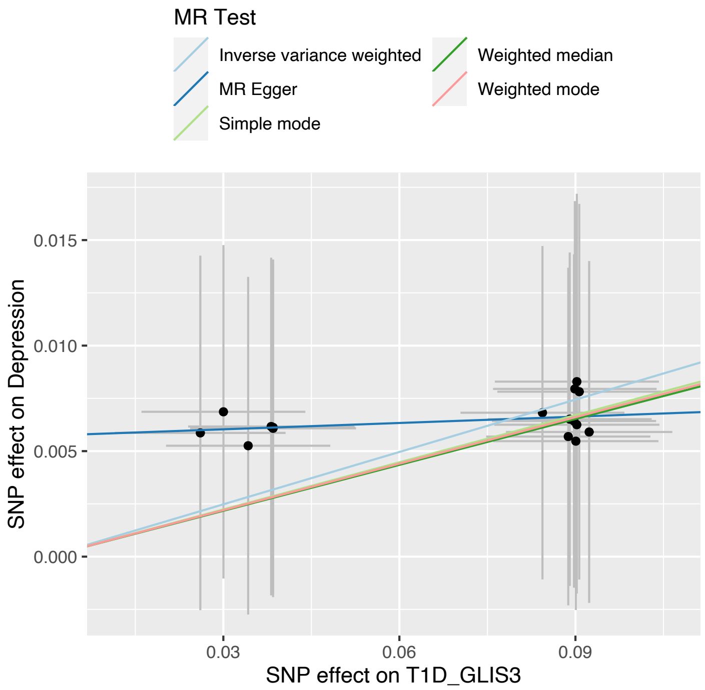

The scatterplot depicts single-nucleotide polymorphism (SNP)–exposure and SNP–outcome associations for each SNP with fitted lines estimated using five Mendelian randomization methods [24]. The point estimates are accompanied by error bars, denoting the standard error of the estimated association between the SNP and the exposure and the outcome, respectively [24].

Supplementary Figure 40 Scatterplots for MR Analyses with Evidence for an Association of T1D with Subsequent Mental Disorders, *GLIS3 Cis* Instrument with Alcohol Dependence

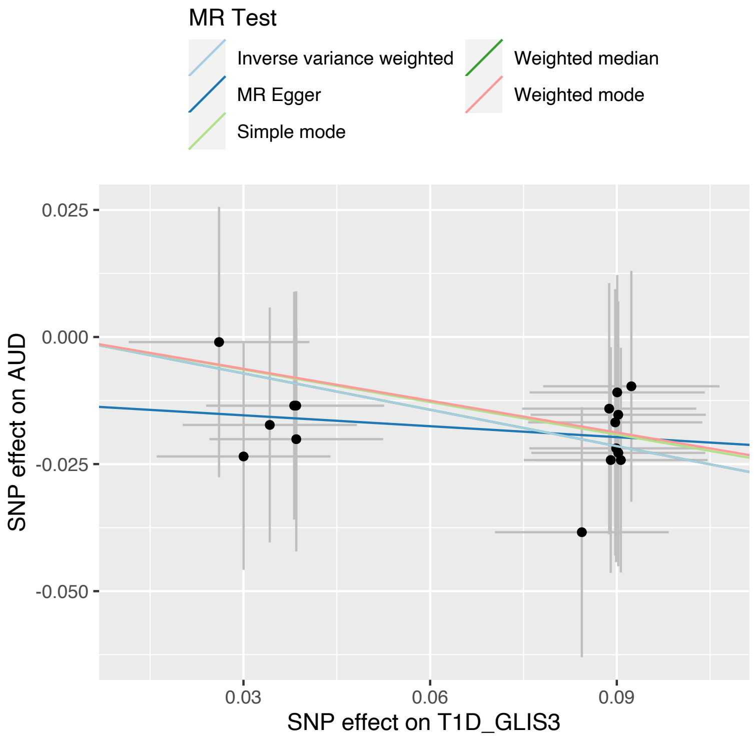

The scatterplot depicts single-nucleotide polymorphism (SNP)–exposure and SNP–outcome associations for each SNP with fitted lines estimated using five Mendelian randomization methods [24]. The point estimates are accompanied by error bars, denoting the standard error of the estimated association between the SNP and the exposure and the outcome, respectively [24].

Supplementary Figure 41 Scatterplots for MR Analyses with Evidence for an Association of T1D with Subsequent Mental Disorders, *IL-2RA Cis* Instrument with Anorexia Nervosa

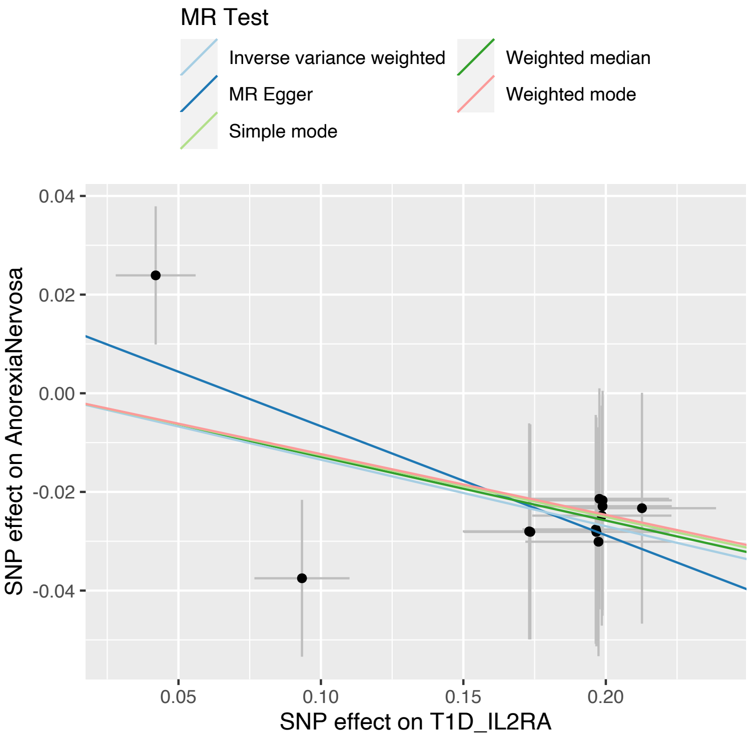

The scatterplot depicts single-nucleotide polymorphism (SNP)–exposure and SNP–outcome associations for each SNP with fitted lines estimated using five Mendelian randomization methods [24]. The point estimates are accompanied by error bars, denoting the standard error of the estimated association between the SNP and the exposure and the outcome, respectively [24].

Supplementary Figure 42 Single SNP Plots for MR Analyses with Evidence for an Association of T1D with Subsequent Mental Disorders, *Trans* Instrument with Schizophrenia

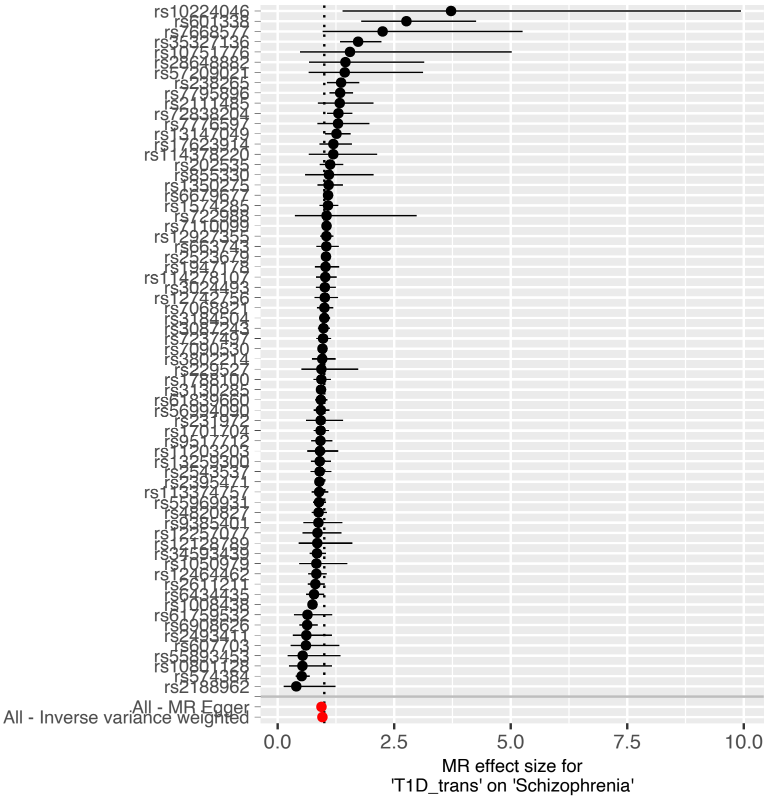

The forest plot depicts individual and combined single-nucleotide polymorphism (SNP) Mendelian randomization-estimated effects. The estimated effects represent the change in odds of outcome per standard deviation increase in genetically-predicted type 1 diabetes risk, and are accompanied by errors bars expressed as 95% confidence intervals.

Supplementary Figure 43 Single SNP Plots for MR Analyses with Evidence for an Association of T1D with Subsequent Mental Disorders, *GLIS3 Cis* Instrument with Schizophrenia

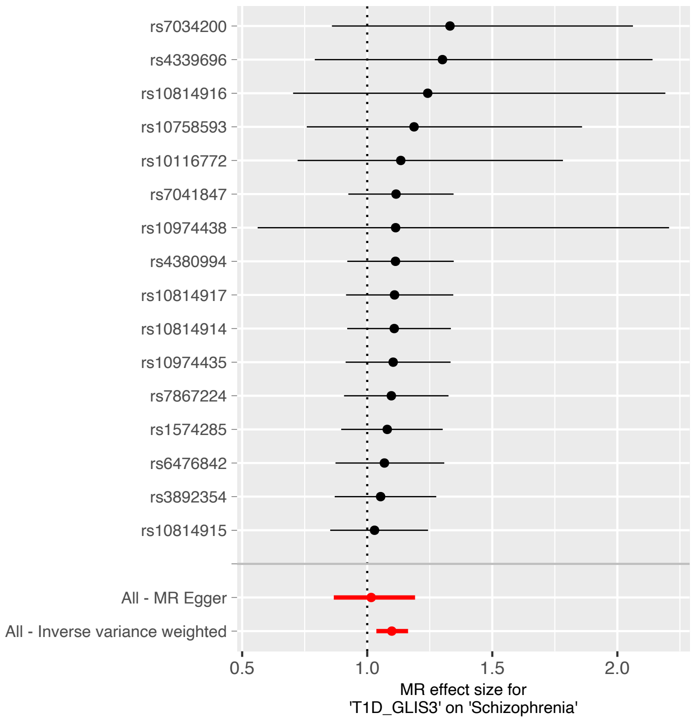

The forest plot depicts individual and combined single-nucleotide polymorphism (SNP) Mendelian randomization-estimated effects. The estimated effects represent the change in odds of outcome per standard deviation increase in genetically-predicted type 1 diabetes risk, and are accompanied by errors bars expressed as 95% confidence intervals.

Supplementary Figure 44 Single SNP Plots for MR Analyses with Evidence for an Association of T1D with Subsequent Mental Disorders, *CTSH Cis* Instrument with Schizophrenia

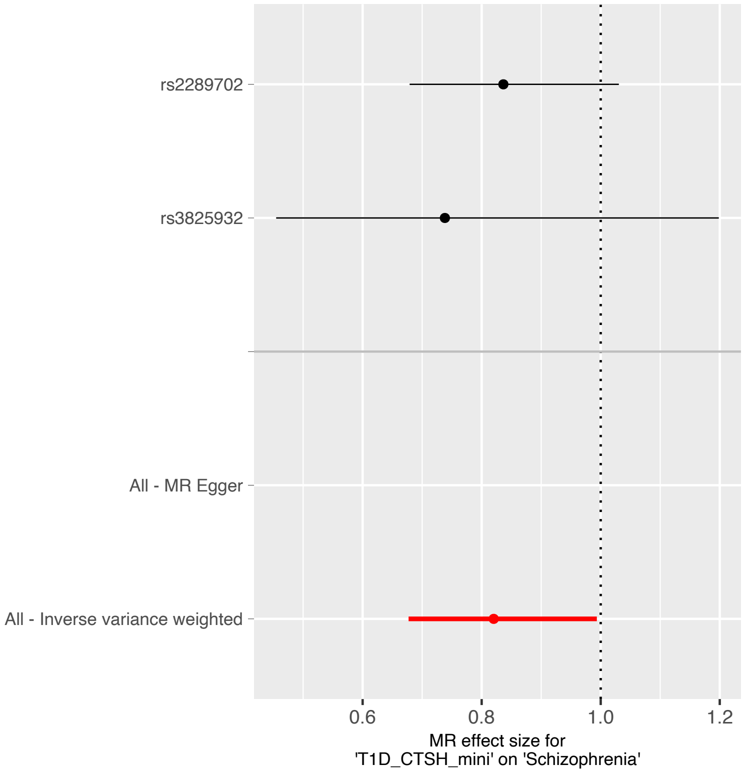

The forest plot depicts individual and combined single-nucleotide polymorphism (SNP) Mendelian randomization-estimated effects. The estimated effects represent the change in odds of outcome per standard deviation increase in genetically-predicted type 1 diabetes risk, and are accompanied by errors bars expressed as 95% confidence intervals.

Supplementary Figure 45 Single SNP Plots for MR Analyses with Evidence for an Association of T1D with Subsequent Mental Disorders, *GLIS3 Cis* Instrument with Anxiety Disorders

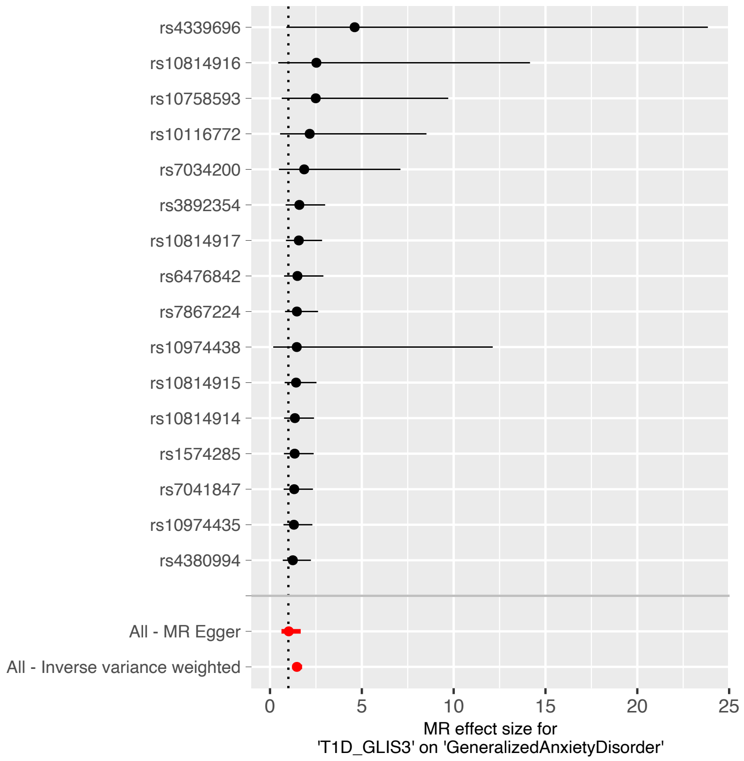

The forest plot depicts individual and combined single-nucleotide polymorphism (SNP) Mendelian randomization-estimated effects. The estimated effects represent the change in odds of outcome per standard deviation increase in genetically-predicted type 1 diabetes risk, and are accompanied by errors bars expressed as 95% confidence intervals.

Supplementary Figure 46 Single SNP Plots for MR Analyses with Evidence for an Association of T1D with Subsequent Mental Disorders, *GLIS3 Cis* Instrument with Major Depressive Disorder

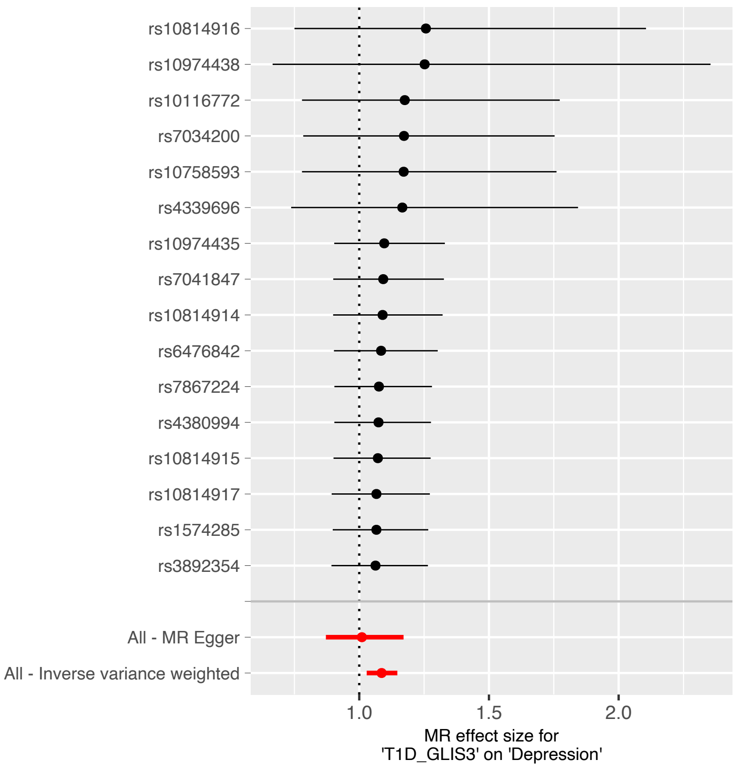

The forest plot depicts individual and combined single-nucleotide polymorphism (SNP) Mendelian randomization-estimated effects. The estimated effects represent the change in odds of outcome per standard deviation increase in genetically-predicted type 1 diabetes risk, and are accompanied by errors bars expressed as 95% confidence intervals.

References

1 Krupchanka, D., Mladá, K., Winkler, P., Khazaal, Y. & Albanese, E. Mortality in people with mental disorders in the Czech Republic: a nationwide, register-based cohort study. *Lancet Public Health* **3**, doi:10.1016/S2468-2667(18)30077-X (2018).

2 Dalsgaard, S. *et al.* Incidence Rates and Cumulative Incidences of the Full Spectrum of Diagnosed Mental Disorders in Childhood and Adolescence. *JAMA Psychiatry* **77**, 155-164, doi:10.1001/jamapsychiatry.2019.3523 (2020).

3 Chubak, J. *et al.* Informative Presence in Electronic Health Record Data: A Challenge in Implementing Study Exclusion Criteria. *Epidemiology* **34**, 29-32, doi:10.1097/EDE.0000000000001542 (2023).

4 VanderWeele, T. J. & Ding, P. Sensitivity Analysis in Observational Research: Introducing the E-Value. *Ann. Intern. Med.* **167**, 268-274, doi:10.7326/M16-2607 (2017).

5 Schneeweiss, S. Sensitivity analysis and external adjustment for unmeasured confounders in epidemiologic database studies of therapeutics. *Pharmacoepidemiology and Drug Safety* **15**, 291-303, doi:10.1002/pds.1200 (2006).

6 Inshaw, J. R. J., Cutler, A. J., Crouch, D. J. M., Wicker, L. S. & Todd, J. A. Genetic Variants Predisposing Most Strongly to Type 1 Diabetes Diagnosed Under Age 7 Years Lie Near Candidate Genes That Function in the Immune System and in Pancreatic beta-Cells. *Diabetes Care* **43**, 169-177, doi:10.2337/dc19-0803 (2020).

7 Kim, Y. S., Nakanishi, G., Lewandoski, M. & Jetten, A. M. GLIS3, a novel member of the GLIS subfamily of Kruppel-like zinc finger proteins with repressor and activation functions. *Nucleic Acids Res* **31**, 5513-5525, doi:10.1093/nar/gkg776 (2003).

8 Yang, Y., Chang, B. H. & Chan, L. Sustained expression of the transcription factor GLIS3 is required for normal beta cell function in adults. *EMBO Mol Med* **5**, 92-104, doi:10.1002/emmm.201201398 (2013).

9 Qi, R., Singh, D. & Kao, C. C. Proteolytic processing regulates Toll-like receptor 3 stability and endosomal localization. *J Biol Chem* **287**, 32617-32629, doi:10.1074/jbc.M112.387803 (2012).

10 Rasschaert, J. *et al.* Toll-like receptor 3 and STAT-1 contribute to double-stranded RNA+ interferon-gamma-induced apoptosis in primary pancreatic beta-cells. *J Biol Chem* **280**, 33984-33991, doi:10.1074/jbc.M502213200 (2005).

11 Floyel, T. *et al.* CTSH regulates beta-cell function and disease progression in newly diagnosed type 1 diabetes patients. *Proc Natl Acad Sci U S A* **111**, 10305-10310, doi:10.1073/pnas.1402571111 (2014).

12 Wang, J. H. *et al.* Aiolos regulates B cell activation and maturation to effector state. *Immunity* **9**, 543-553, doi:10.1016/s1074-7613(00)80637-8 (1998).

13 Cortes, M. & Georgopoulos, K. Aiolos is required for the generation of high affinity bone marrow plasma cells responsible for long-term immunity. *J Exp Med* **199**, 209-219, doi:10.1084/jem.20031571 (2004).

14 Schmiedel, B. J. *et al.* 17q21 asthma-risk variants switch CTCF binding and regulate IL-2 production by T cells. *Nat Commun* **7**, 13426, doi:10.1038/ncomms13426 (2016).

15 Mehta, M. *et al.* Themis-associated phosphatase activity controls signaling in T cell development. *Proc Natl Acad Sci U S A* **115**, E11331-E11340, doi:10.1073/pnas.1720209115 (2018).

16 Trynka, G. *et al.* Dense genotyping identifies and localizes multiple common and rare variant association signals in celiac disease. *Nat Genet* **43**, 1193-1201, doi:10.1038/ng.998 (2011).
17 Jostins, L. *et al.* Host-microbe interactions have shaped the genetic architecture of inflammatory bowel disease. *Nature* **491**, 119-124, doi:10.1038/nature11582 (2012).
18 Burgess, S., Butterworth, A. & Thompson, S. G. Mendelian randomization analysis with multiple genetic variants using summarized data. *Genet Epidemiol* **37**, 658-665, doi:10.1002/gepi.21758 (2013).
19 Bowden, J., Davey Smith, G., Haycock, P. C. & Burgess, S. Consistent Estimation in Mendelian Randomization with Some Invalid Instruments Using a Weighted Median Estimator. *Genet Epidemiol* **40**, 304-314, doi:10.1002/gepi.21965 (2016).
20 Bowden, J., Davey Smith, G. & Burgess, S. Mendelian randomization with invalid instruments: effect estimation and bias detection through Egger regression. *Int J Epidemiol* **44**, 512-525, doi:10.1093/ije/dyv080 (2015).
21 Bowden, J. *et al.* A framework for the investigation of pleiotropy in two-sample summary data Mendelian randomization. *Stat Med* **36**, 1783-1802, doi:10.1002/sim.7221 (2017).
22 Verbanck, M., Chen, C. Y., Neale, B. & Do, R. Detection of widespread horizontal pleiotropy in causal relationships inferred from Mendelian randomization between complex traits and diseases. *Nat Genet* **50**, 693-698, doi:10.1038/s41588-018-0099-7 (2018).
23 Burgess, S. Sample size and power calculations in Mendelian randomization with a single instrumental variable and a binary outcome. *Int. J. Epidemiol.* **43**, 922-929, doi:10.1093/ije/dyu005 (2014).
24 Sanderson, E. *et al.* Mendelian randomization. *Nature Reviews Methods Primers* **2**, 6, doi:10.1038/s43586-021-00092-5 (2022).
